# Fullerene Desymmetrization as a Means to Achieve Single‐Enantiomer Electron Acceptors with Maximized Chiroptical Responsiveness

**DOI:** 10.1002/adma.202004115

**Published:** 2020-11-23

**Authors:** Wenda Shi, Francesco Salerno, Matthew D. Ward, Alejandro Santana‐Bonilla, Jessica Wade, Xueyan Hou, Tong Liu, T. John S. Dennis, Alasdair J. Campbell, Kim E. Jelfs, Matthew J. Fuchter

**Affiliations:** ^1^ Department of Chemistry and Molecular Sciences Research Hub Imperial College London White City Campus, 82 Wood Lane London W12 0BZ UK; ^2^ Center for Processable Electronics Imperial College London South Kensington Campus London SW7 2AZ UK; ^3^ Department of Physics Imperial College London South Kensington Campus London SW7 2AZ UK; ^4^ School of Physics and Astronomy and Materials Research Institute Queen Mary University of London Mile End Road London E1 4NS UK

**Keywords:** chiral materials, chiroptical response, circularly polarized light, fullerenes, organic field‐effect transistors

## Abstract

Solubilized fullerene derivatives have revolutionized the development of organic photovoltaic devices, acting as excellent electron acceptors. The addition of solubilizing addends to the fullerene cage results in a large number of isomers, which are generally employed as isomeric mixtures. Moreover, a significant number of these isomers are chiral, which further adds to the isomeric complexity. The opportunities presented by single‐isomer, and particularly single‐enantiomer, fullerenes in organic electronic materials and devices are poorly understood however. Here, ten pairs of enantiomers are separated from the 19 structural isomers of bis[60]phenyl‐C61‐butyric acid methyl ester, using them to elucidate important chiroptical relationships and demonstrating their application to a circularly polarized light (CPL)‐detecting device. Larger chiroptical responses are found, occurring through the inherent chirality of the fullerene. When used in a single‐enantiomer organic field‐effect transistor, the potential to discriminate CPL with a fast light response time and with a very high photocurrent dissymmetry factor (*g*_ph_ = 1.27 ± 0.06) is demonstrated. This study thus provides key strategies to design fullerenes with large chiroptical responses for use as chiral components of organic electronic devices. It is anticipated that this data will position chiral fullerenes as an exciting material class for the growing field of chiral electronic technologies.

## Graphical abstract

The isomeric complexity of a common organic fullerene electron acceptor is resolved, giving ten enantiomeric pairs across six bond types with different chiroptical response. Single‐enantiomer fullerene transistor devices discriminate circularly polarized light with a fast light response and very high photocurrent dissymmetry factor (up to *g*_ph_ = 1.2 ± 0.06).

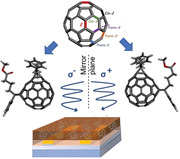

Fullerene derivatives have played a hugely important role in the development of organic electronic devices, particularly organic solar cells. They remain the most widely used electron acceptors in organic/hybrid photovoltaic devices (OPVs),^[^ ^1^, ^2^, ^3^, ^4^ ^]^ while also demonstrating excellent photo responsivity as broadband photodetectors.^[^ ^5^, ^6^, ^7^, ^8^, ^9^, ^10^ ^]^ Parent fullerene structures have extremely challenging solubility, which limits their processability and miscibility with host materials in bulk heterojunction OPVs; an issue which has been addressed through the production of more soluble derivatives using simple cycloaddition chemistry. Bis[60]PCBM (PCBM = phenyl‐C61‐butyric acid methyl ester) (dimethyl 4,4′‐[61,62‐diphenly,3′H,3″H‐dicyclopropa(C_60_—I*_h_*)[5,6]fulleren‐1,9:X,Y‐diyl]dibutanoate) is one such derivative and one of the most utilized solubilized fullerenes, which is easy to synthesize, highly processable, and solar cells based on this material have high power conversion efficiencies.^[^ ^11^, ^12^, ^13^, ^14^, ^15^, ^16^, ^17^, ^18^ ^]^ The addition of two solubilizing addends to C_60_ results in a large number of structural isomers however, each with varying energetic properties (influencing device voltage) and morphological properties (influencing device current). Despite this, bis[60]PCBM is synthesized and used as an isomeric mixture, and the role of individual isomers on morphological, spectroscopic, and device performance is very poorly understood. In a previous study, Dennis and co‐workers separated the 19 structural isomers of bis[60]PCBM and characterized them using a combination of NMR, UV–vis, and retention times by HPLC.^[^ ^19^, ^20^ ^]^ Although the performance of a single structural isomer bis[60]PCBM in organic devices was not investigated, isomerically‐pure fullerene derivatives have generally been reported to exhibit higher power conversion efficiency than the corresponding isomeric mixture.^[^ ^21^, ^22^ ^]^ This implies that the further study of single bis[60]PCBM isomers may hold significant promise.

The complex isomeric composition of bis[60]PCBM is not limited to structural isomers however. Of the synthetically accessible 19 bis[60]PCBM isomers, 13 are chiral, which gives rise to 13 pairs of enantiomers (see **Figure** **1**). It is interesting to consider what opportunities could emerge from access to enantiomerically pure fullerenes. To date, chiral fullerene derivatives have mostly been explored as an academic curiosity and in limited applications, with a notable exception perhaps being enantioselective catalysis.^[^ ^23^ ^]^ Given the importance of this material class to organic electronic devices, it is curious that single‐enantiomer fullerene devices are yet to be explored in this context. This may be because the opportunities provided by chiral organic semiconducting materials are, in general, underexploited in technological applications.^[^ ^24^ ^]^ Nonetheless, it is likely that chiral composition dependent effects on device performance would be observed,^[^ ^25^, ^26^ ^]^ as would the potential to render fullerene devices sensitive to circularly polarized light (CPL), through preferential absorption of left or right‐handed CPL (circular dichroism, CD). Such circularly polarized (CP) functionality would potentially have far‐reaching applications, including in enantioselective sensing, optical communication, and quantum computation.^[^ ^27^, ^28^, ^29^, ^30^, ^31^, ^32^, ^33^, ^34^ ^]^

**Figure 1:**
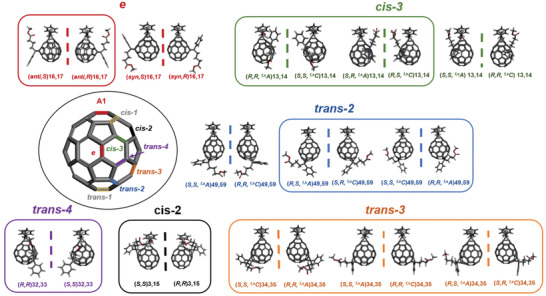
The 13 pairs of bis[60]PCBM enantiomers, categorized into six bond types according to the positioning of the second adduct relative to 1,9 position of the first addend (A1): *cis*‐2 (black), *cis*‐3 (green), *e* (red), *trans*‐2 (blue), *trans*‐3 (orange) and *trans*‐4 (purple) with dotted mirror planes depicted. For each isomer, *R*/*S* descriptors refer to addend stereochemistry and *^f,s^C*/*^f,s^A* to the fullerene stereochemistry using the method defined by Diederich and Thilgen.^[^ ^46^ ^]^ All isomer labels should be proceeded by “bis[60]PCBM”, i.e., (*S*,*S*,*^f,s^C*)13,14 becomes (*S*,*S*,*^f,s^C*)13,14‐bis[60]PCBM. The enantiomeric pairs contained within boxes represent those isolated in this study. The stereochemical nomenclature system is further described in the Supporting Information. Files with rotatable structures for each enantiomer and larger images with more viewpoints are included as Supporting Information.

In particular, chiral conjugated materials with excellent CD have strong potential in the development of CPL detectors, either transistors or diodes.^[^ ^35^, ^36^, ^37^ ^]^ The first CPL sensitive photodiode was developed by Meskers and co‐workers in 2010, utilizing a polyfluorene with chiral side chains.^[^ ^35^ ^]^ In 2013, Fuchter, Campbell, and co‐workers demonstrated the first (and currently only) CPL sensitive phototransistor (CP‐photoFET), using a chiral small molecule helicene.^[^ ^36^ ^]^ Other approaches recently reported for CP detection in devices include self‐assembled small molecules, metamaterials, and perovskite hybrid materials.^[^ ^37^, ^38^, ^39^, ^40^, ^41^, ^42^ ^]^ Despite these emerging approaches that use an array of material classes, organic molecules remain highly attractive for the development of CPL photodetectors, due to their tuneable optical characteristics, mechanical flexibility, low processing cost, and scalability (through printing, etc.).

In this work, we separate and systematically study ten pairs of enantiomers from the 19 structural isomers of bis[60]PCBM. We analyze the chiroptical responses of these isomers and elucidate the origin of larger absorption dissymmetry (*g*_abs_ factors) in some isomers over others. Specifically, we show that larger chiroptical responses occur in cases with greater asymmetry of the electron distribution within the fullerene cage. We showcase the application of our single‐enantiomer bis[60]PCBM materials in the production of single‐enantiomer CP‐photoFET devices, with very high photocurrent dissymmetry factors (up to *g*_ph_ = 1.27 ± 0.06). Thus, our study determines a clear set of design principles to generate chiral fullerene derivatives with maximized chiroptical activity, and with significant potential in the burgeoning field of chiral (nano)technology and devices.

For bridged C_60_ monoadducts, such as PCBM, without considering the configuration of the first addend, there are eight remaining non‐equivalent double bonds susceptible to the addition of a second addend. Formation of the bisadduct therefore gives rise to eight regioisomers: *cis*‐1, *cis*‐2, *cis*‐3, *e*, *trans*‐1, *trans*‐2, *trans*‐3, and *trans*‐4 (Figure 1).^[^ ^43^, ^44^, ^45^ ^]^ Bond type naming is defined by the relative position of the second addend with respect to the first. When both addends are found on the same hemisphere, the isomer is named *cis*, if on the opposite hemisphere, *trans*, and if on the equatorial plane, *e*. This naming system also reflects the electronic properties of the isomers, where structural isomers of the same bond type have similar UV–vis absorption spectra.^[^ ^19^, ^43^ ^]^ Here, we only consider six bond types since the *cis*‐1 isomer is not formed during bis[60]PCBM synthesis in any appreciable amount due to steric effects and the *trans*‐1 isomer is not chiral due to the presence of a mirror plane (Figure 1). Of the structural isomers contained within these six remaining bond type groups, 13 pairs of enantiomers can be theoretically predicted according to their symmetry properties.^[^ ^20^ ^]^

In general terms, fullerene molecules can be chiral by virtue of a chiral carbon cage (for example, in higher fullerenes, such as C_76_, and C_84_), stereogenic elements in the addends, or from the geometric arrangement of the addends via a chiral addition pattern. In the case of the bis[60]PCBM derivative employed in this study – the one principally used in organic electronic devices, as for others C_60_ bisadducts previously reported in literature,^[^ ^46^, ^47^ ^]^ some structural isomers are inherently chiral while others are non‐inherently chiral. In the former case, the isomers are chiral due to the desymmetrization of the C_60_ cage by the particular addition pattern, regardless of the nature of the addends. In the latter case, the dissymmetry arises exclusively from the stereogenic units of the addends.^[^ ^46^ ^]^ In a previous study, Dennis and co‐workers named the structural isomers of bis[60]PCBM based on the location of the second addend and the symmetry, for example, (C_1_)21,40‐bis[60]PCBM.^[^ ^20^ ^]^ Here, we adapt that nomenclature to explicitly include descriptors of fullerene chirality and the stereochemistry of the addends, according to the systematic system devised by Diederich and Thilgen.^[^ ^46^ ^]^ The assigned nomenclature used is given in Figure 1 and our naming system is further described in the Supporting Information.

The 19 structural isomers were isolated using the previously reported method and identified by UV–vis, ^13^C NMR, and retention time (i.e., polarity).^[^ ^19^, ^20^ ^]^ The 13 chiral isomers of bis[60]PCBM, obtained as racemates, were subjected to further purification via peak‐recycling HPLC using chiral columns (Chiralpak IE and IF). Ten out of 13 pairs of enantiomers were successfully resolved and purified to > 99%, which represents at least one enantiomeric pair for every bond type isomer. This isomeric series provides the means to systematically study the chiroptical response of the chiral fullerene isomers as a function of substitution pattern and symmetry for the first time.

To assign the absolute configuration of the isolated bis[60]PCBM stereoisomers, we compared experimentally obtained CD spectra with the simulated spectra. Such an approach is increasingly common for assignment of the absolute configuration and has been applied previously to chiral fullerenes.^[^ ^48^, ^49^ ^]^ The bis[60]PCBM isomeric structures were optimized at the B3LYP/6‐31G(d) level. Under the assumption that the extended butyric acid addend substituent will have little impact on the chiroptical properties of the fullerene, this moiety was truncated to a methyl group for all the computational structures under study. The excellent correlation of experimental and calculated CD spectra later confirmed this assumption. UV–vis and CD spectra were calculated via time‐dependent density functional theory (TD‐DFT) for the first 50 excited states at the B3LYP/6‐31G(d) level of theory and compared to experimental spectra using SpecDis.^[^ ^50^ ^]^ A similarity factor higher than 0.8 was obtained for all of the ten isomers. Therefore, the absolute configuration of the enantiomeric pairs obtained could be assigned with high degree of confidence as shown in Table S1, Supporting Information. All spectra and assignments can be found in the Supporting Information.

Due to the high number of π→π* transitions located in the UV–vis region (see Supporting Information for full set of UV–vis and CD spectra), spectroscopically very rich CD spectra are observed (Figure S4, Supporting Information). As would be expected, all of the isolated pairs of enantiomers show an equal and opposite CD response. Importantly, diastereomers belonging to the same bond type (*cis*‐2, *cis*‐3, *trans*‐2, etc.) display almost identical CD spectra, with only minor differences in amplitude and excitation energy, that can be therefore considered an inherent stereochemical fingerprint of each addition pattern of these bis[60]PCBM isomers. Addend substituent stereochemistry appears therefore to have a negligible impact on the spectra shape, as has been previously observed in specific examples of similar systems.^[^ ^51^ ^]^ However, we also observe that the band peak maxima for isomers of the same bond‐type group can differ in magnitude up to ≈30%. This is likely due to a match/mismatch effect given by the different reciprocal orientation of the two addends on the C_60_ cage. From an application point of view, the variety of CD responses contained within this series allows access to CPL responsivity across a large region of the UV–vis spectrum of potential usage in CPL detecting devices.

To further investigate how the chiroptical properties observed relate to the isomeric structures of this series, the experimental absorption dissymmetry factor *g*_abs_ = Δε/ε was analyzed. This value provides a dimensionless measure of the chiroptical response of a given molecule, normalized by absorption. The *g*_abs_ factors of all the stereoisomers, ordered by bond type, are shown in **Figure** **2**a for representative enantiomers. The magnitude of *g*_abs_ varies by approximately two orders of magnitude as a function of stereoisomeric series, from 10^−4^ to 10^−3^ (typical of chiral organic small molecules) up to the value of 3 × 10^−2^. Values of this order of magnitude are highly uncommon for an isolated molecule in solution.^[^ ^52^ ^]^ Moreover, a particularly large chiroptical response occurs at ≈600 nm, a longer wavelength than the one typically achievable with other conjugated chiral small molecules, such as helicenes, biraryls, and cyclophanes.^[^ ^52^ ^]^ Theoretically calculated *g*_abs_ at the B3LYP/6‐31G(d) level of theory are of the same order magnitude, therefore in good agreement, with the experimental values (both experimental and simulated values at the peaks maxima are listed in Table S3, Supporting Information).

**Figure 2:**
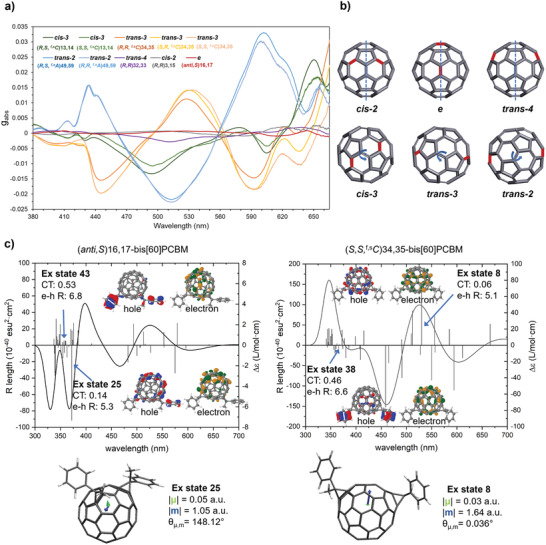
a) Absorption dissymmetry factor *g*_abs_ of one enantiomer per pair of all the isolated bis[60]PCBM enantiomers. b) Six addition patterns on the cage (two types): non‐inherently chiral with mirror symmetry (upper row) and inherently chiral with C_2_ symmetry (bottom row). c) Calculated CD spectra of the *e* isomer (anti,S)16,17 (left) and the *trans*‐3 isomer (*S,S, ^f,s^C*)34,35 (right). The insets highlight the states with highest rotatory strength (state 25 and state 8) and the states with highest charge‐transfer character (state 43 and state 38). CT refers to the degree of charge‐transfer character and *e*‐*h R* indicates the root mean square distance of the electron–hole pair. The molecular pictures represent the distribution of the pair of “hole” (where the electron leaves upon excitation) and “electron” (where the excited electron goes to) for that excited state. On the bottom, electric and magnetic transition dipoles for the excited state 25 and 8 are displayed.

The six bond types can be further divided into two groups according to their *g*_abs_ values. Stereoisomers from *cis*‐2, *e*, and *trans*‐4 show a relatively low *g*_abs_ value (≈10^−3^), while stereoisomers from *cis*‐3, *trans*‐2, and *trans*‐3 show a *g*_abs_ value of up to one order of magnitude higher (≈10^−2^). This significant difference in chiroptical response can be qualitatively rationalized using symmetry‐based arguments. In the group of isomers with lower *g*_abs_, the addition pattern on the C_60_ cage (without considering the nature of the addends) contains a mirror plane as shown in Figure 2b, upper row. As described above, bis[60]PCBM isomers of these bond type groups (*cis*‐2, *e*, and *trans*‐4) are non‐inherently chiral; that is the chirality arises only by virtue of the stereogenic centers on the addends. This is equivalent to stating that the chromophore is fundamentally achiral and the chiroptical response derives only from perturbation of the substituents. The group of isomers with higher *g*_abs_, instead, has addition patterns with C_2_ symmetry (*cis*‐3, *trans*‐2, and *trans*‐3); these bis[60]PCBM isomers are therefore said to be inherently chiral. This means the fullerene chromophore is inherently chiral and therefore the chiroptical response would be expected to increase. Ultimately, this difference in symmetry determines the distortion of π‐electron distribution in the cage, with achiral addition patterns leading to more uniform electron distributions than those that are chiral.

TD‐DFT results were further analyzed using the software TheoDORE, which allows the study of the nature of the excited states in greater detail by employing the natural transition orbital (NTO) formalism.^[^ ^53^ ^]^ For a given excitation, NTOs can be associated to a hole–electron pair, making it easier to visualize the movement of charges across different molecular fragments. This enables one to assess the role of charge‐transfer (CT) character in the π→π* transitions of different isomers and how this relates to their chiroptical properties.^[^ ^54^ ^]^ We observe that excited states with high rotatory strength (*R*) are generally states with low CT character. This is more clearly observed for inherently chiral substitution patterns (*cis*‐2, *trans*‐2, *trans*‐3) as shown in Figure S6, Supporting Information. In Figure 2c, we show part of the analysis for the *e* and *trans*‐3 isomers (full analysis can be found in Supporting Information). In both cases, for the states with the highest charge‐transfer character, the transition occurs as a movement of π‐electrons from the C_60_ to the addends. In contrast, the states with highest rotatory strength (state 25 for *e* and state 8 for *trans*‐2) are described by a strong rotational movement of π‐electrons solely on the surface of the cage with very low CT character (weak translation of charges). It is well known that the *g* factor of a chiral chromophore, is defined by Equation (1):^[^ ^55^ ^]^

(1) $$g\;\;=\;\;\frac{4|m|\cdot |\mu |\cdot \;\cos\theta }{|m{|}^{2}+|\mu {|}^{2}}$$

where **m** is the magnetic transition dipole of the chromophore, **μ** is the electric transition dipole, and θ is the angle between **m** and **μ**.

We observe that the magnetic transition dipole moment is significantly higher in magnitude than the electric transition dipole for those excited states with high rotatory strength. Moreover, these states are characterized by a very small angle θ when the chromophore is chiral, implying a strong coupling between **m** and **μ**. This likely account for the higher order of magnitude of *g*_abs_ observed in those isomers with inherently chiral substitution pattern. The distance between the addends seems not to play a crucial role since states with high rotatory strength are mainly local and do not involve charge delocalization onto the addends. Other chiral aromatic molecules with high dissymmetry factors due to the strong magnetic transition dipole are known in the literature.^[^ ^56^ ^]^ The reason is likely related to two factors: the curved surface of the backbone which encourages a circular movement of valence electrons and the aromatic characteristics (locally and globally) arising from the desymmetrization of the chromophore (the latter is analyzed in more depth in the Supporting Information). The modulation of these two factors allows the enhancement of the interaction of the molecule with the magnetic part of the CP excitation light and should be taken into consideration for the chemical design of new aromatic materials with good chiroptical properties.

With access to single‐stereoisomer bis[60]PCBM molecules, we sought to showcase their application in the context of an organic electronic device. Enantiomers of (anti,*R*)16,17‐bis[60]PCBM (*e*) and (*R,R, ^f,s^A*)49,59‐bis[60]PCBM (*trans*‐2) were separately used to build bottom–gate, bottom–contact organic field‐effect transistors (OFETs) on prepatterned substrates. The transfer characteristics of these devices in the dark (**Figure** **3**a,b) showed turn‐on voltages of ≈15–20 V, low off‐currents (1 × 10^−11^ A), and on/off ratios of 10^3^. To the best of our knowledge, these devices represent the first OFETs prepared from a single‐isomer bis[60]PCBM material. The performance of these devices could be further optimized through alternative device architecture, fabrication procedure, etc. The device characteristics of the racemic (anti,*R/S*)16,17‐bis[60]PCBM isomer displays comparable device performance under dark conditions (with the exception of a greater turn‐on voltage) and is included in the Supporting Information.

**Figure 3:**
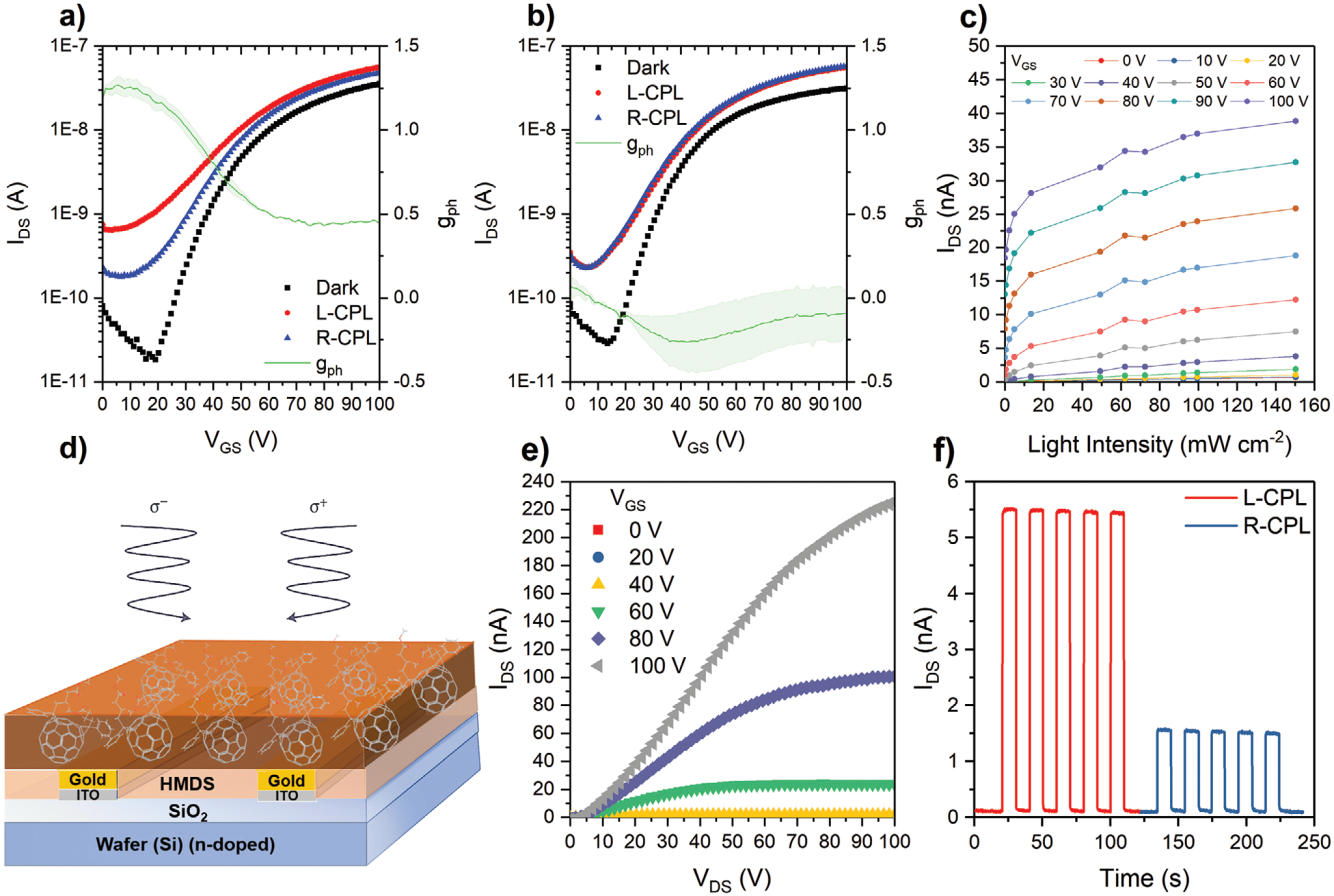
Device characteristics of bis[60]PCBM enantiomer pure photo‐FETs. a,b) Variation of transfer curves of (anti,*R*)16,17‐bis[60]PCBM (a) and (*R,R, ^f,s^A*)49,59‐bis[60]PCBM (b) upon exposure to left‐handed and right‐handed CPL with 95 and 0.34 mW cm^−2^, respectively, compared to curves in the dark. *V*_DS_ = 20 V. Dissymmetry factors for the photocurrent generation (g_ph_) and its associated error are given by the green curve and shaded area, respectively. c) The light‐intensity response of the (anti,*R*)16,17‐bis[60]PCBM photoFET from 0.48 to 150 mW cm^−2^ at different gate voltage from 0 to 100 V, *V*_DS_ = 20 V. d) The device structure of photoFETs. e) Output characteristics of (anti,*R*)16,17‐bis[60]PCBM photoFET at gate voltage between 0 and 100 V. f) Time‐resolved left‐ and right‐handed CPL photoresponse was measured at 405 nm with an intensity of 92.3 mW cm^−2^, a frequency of 0.05 Hz and *V*_DS_ = *V*_GS_ = 40 V, showing a high stability and reversibility.

The use of an enantiomerically pure semiconductor enables the photoresponse of OFETs to be sensitive to the circular polarization of light. While there are a number of emerging material and device approaches to CPL detection, only one CP‐photoFET has been previously reported, by Fuchter, Campbell, and co‐workers, using an enantiomerically pure helicene.^[^ ^36^ ^]^ To study the CPL responsivity of the enantiopure fullerene‐based transistors, single‐enantiomer OFETs were illuminated using a laser diode at 405 nm, which corresponds to the most easily accessible CD peaks at the onset of absorption for these enantiomers. The transfer characteristics of these devices under left handed CPL and right handed CPL are shown in Figure 3a,b. The off currents of the devices increase from 10^−11^ to 10^−10^ A, and the turn‐on voltages shift from 15 to 20 V to ≈10 V when exposed to CPL. From this data, it is possible to extract a *g* factor for the photocurrent generation (*g*_ph_), which is up to −0.26 ± 0.18 for the (*R,R, ^f,s^A*)49,59‐bis[60]PCBM device and an impressive 1.27 ± 0.06 using the (anti,*R*)16,17‐bis[60]PCBM photoFET (Figure 3a,b). This large and selective CP photoresponse is of potential importance for CPL detectors and suggests significant promise for enantiomerically pure fullerene isomers in such an application. We note that for both photoFETs, *g*_ph_ is significantly larger than *g*_abs_ (at 405 nm). This outcome is consistent with the previously reported helicene CP‐photoFET.^[^ ^36^ ^]^ We propose that the enhancement of *g*_ph_ relative to *g*_abs_ arises from two cooperative device mechanisms: the CP selective photogeneration of electrons in the channel, which increases the majority carrier density and the CP selective photogeneration of holes, which accumulate at the source electrode, reducing the barrier to electron injection. This model is described in full in the Supporting Information for the (anti,*R*)16,17‐bis[60]PCBM device.

Owing to the superior CP‐selective properties of the (anti,*R*)16,17‐bis[60]PCBM photoFET, the characteristics of this device were further studied. The output curve of (anti,*R*)16,17‐bis[60]PCBM as an n‐type material produced well‐behaved linear and saturated regions as shown in Figure 3e. The transfer curves of this enantiomer was also measured under the saturation regime (Figure S13, Supporting Information), yielding a saturation electron mobility of 1.44 × 10^−5^ V^−1^ s^−1^. The light intensity and time dependent CP photoresponse of the chiral fullerene‐based devices was also explored. These CP photoFETs were very sensitive to light even at low optical power of 0.48 mW cm^−2^, where an increase of the off current of 1.4 times was observed compared to that in the dark (Figure 3c). The steady state photocurrent gradually increased and did not reach the saturation from 0.48 to 150 mW cm^−2^. The time‐dependent photoresponse shows fast symmetric rise time and decay time with sharp square shape as well as an obvious dissymmetric CP response shown in Figure 3f, a substantial improvement and better reversibility over the long decay seen in the helicene CP‐photoFETs.^[^ ^36^ ^]^ The rise and fall times of these devices are less than 43 ms (Figure S14, Supporting Information), which is much shorter than reported for [60]PCBM single‐crystal photodetector (350–650 ms).^[^ ^8^ ^]^ Therefore, a device with such a fast light response could in principle be applied to detect higher frequency CPL for lower latency information transmission.

We successfully separated and assigned the absolute configurations to ten pairs (six bond types) of bis[60]PCBM enantiomers, one of the most widely used fullerene bisadducts for optoelectronic devices. The broadband UV–visible–NIR chiroptical response indicates great potential in diverse applications and the possibility of tuning device detection wavelength by employing different isomers. We fabricated the first photoFETs based on enantiomerically pure fullerenes and showed they were able to discriminate CPL in a highly effective manner. The devices gave an excellent *g*_ph_ (up to 1.27 ± 0.06) and fast response times of less than 43 ms. Such promising properties can be further applied in CP organic photodiodes and other CP photodetecting technologies. For example, the use of patterning techniques—available for solution‐processable organic semiconductors—should allow the creation of arrays of CPL‐sensitive devices, as well as integration with complementary metal–oxide–semiconductor (CMOS) electronics. Overall, this study further develops chiral organic materials for CPL detection, positioning enantiopure fullerenes as an exciting material class in such chiral technologies.

## Conflict of Interest

The authors declare no conflict of interest.

## Supporting information

Supporting Information

Supporting Information
