# Supplementary material for: Fullerene Desymmetrization as a Means to Achieve Single‐Enantiomer Electron Acceptors with Maximized Chiroptical Responsiveness
Source: Adv Mater. 2020 Nov 23;33(1):2004115. doi: 10.1002/adma.202004115 (PMC11468824; doi:10.1002/adma.202004115)
Supplement: Supplementary file 1 — Supporting Information [file ADMA-33-2004115-s001.pdf]

© 2020 Wiley-VCH GmbH

ADVANCED MATERIALS

Supporting Information

for *Adv. Mater.*, DOI: 10.1002/adma.202004115

Fullerene Desymmetrization as a Means to Achieve Single-Enantiomer Electron Acceptors with Maximized Chiroptical Responsiveness

*Wenda Shi, Francesco Salerno, Matthew D. Ward, Alejandro Santana-Bonilla, Jessica Wade, Xueyan Hou, Tong Liu, T. John S. Dennis, Alasdair J. Campbell, Kim E. Jelfs, and Matthew J. Fuchter**

Fullerene desymmetrization as a means to achieve single enantiomer electron acceptors with maximized chiroptical responsiveness

Wenda Shi[a†], Francesco Salerno[a,b†], Matthew D. Ward[b,c], Alejandro Santana-Bonilla[a], Jessica Wade[a,b,c], Xueyan Hou[d], Tong Liu[d], T. John S. Dennis[d], Alasdair J. Campbell[b,c], Kim E. Jelfs[a,b] and Matthew J. Fuchter[a,b]*

*[a]Department of Chemistry and Molecular Sciences Research Hub, Imperial College London, White City Campus, 82 Wood Lane, London W12 0BZ, UK, [b]Centre for Processable Electronics, Imperial College London, South Kensington Campus, London SW7 2AZ, UK, [c]Department of Physics, Imperial College London, South Kensington Campus, London SW7 2AZ, UK, [d]School of Physics and Astronomy and Materials Research Institute, Queen Mary University of London, Mile End Road, London E1 4NS, UK, *e-mail: m.fuchter@imperial.ac.uk*

Contents

Methods....3
HPLC chromatograms during purification process....5
Absolute configuration assignment....17
Spectroscopic Characterization....18
Description of the nomenclature system....25
Simulated UV-Vis and CD spectra....28
Excited States analysis....33
Excited States analysis discussion....51
Different viewpoints of all the enantiomers....56
Thin film characterisation....69
Device Measurements....72
References....79

Methods

Synthesis and purification

The isomeric bis[60]PCBM mixture was synthesized in three steps according to the reference method.[1] NMR spectra of all the structural isomers can be found in the previous work.[2] The racemic structural isomers were separated following the previously reported method, via silica column (Waters Sunfire 19 mm I.D × 150 mm), 5PBB column (Nakalai Tesque Cosmosil 20 mm × 250 mm) and 5PYE column (Nakalai Tesque Cosmosil 20 mm I.D. × 250 mm) with flow rate 18 mL min^{-1} and 312 nm operating wavelength of the UV detector of peak-recycling HPLC.[3,4] Following isolation of all 19 structural isomers in >99% purity, 10 pairs of enantiomers were purified using a ChiralPak IE column (10 mm I.D. × 250 mm) and ChiralPak IF (10 mm I.D. × 250 mm) with 5mL min^{-1} flow rate on the same peak-recycling HPLC. All HPLC work were conducted with Japan Analytical Industry LC-908 HPLC with toluene mobile phase.

CD and UV-Vis

CD spectra of each enantiomer were recorded in toluene around 0.2 mg/ml on a JASCO J-810 CD spectrophotometer under ambient temperature. The scanning range selected was from 360 nm to 700 nm with a 200 nm min^{-1} rate and a bandwidth of 1 nm. Data were collected with three times accumulation, which means the machine samples three times and generates a spectrum. Thin film CD spectra were acquired using a Chirascan from Applied Photophysics. UV-Vis were measured using an Agilent Cary 5000 UV-Vis spectrophotometer.

Microscopy

Topographic atomic force micrographs were acquired using a Park NX10 in non-contact mode on both Si/SiO_2 (OFET) and quartz substrates. The scanning speed was 1 Hz and the image size was 512 px × 512 px. Cross-polarised microscope images were acquired using a Zeiss microscope and digital camera at 10 x optical magnification.

Calculations

All DFT calculations were carried out employing Gaussian'16.[4] Under the assumption that the alkyl chains have negligible impact on the chiroptical properties of the whole molecule, the addends of all the structures were first 'trimmed' to leave a methyl group. The phenyl group was preserved. The geometries were relaxed including a solvent model IFPCM for toluene using B3LYP/6-31G(d) method. TD-DFT calculations (50 excited states with singlet symmetry) were then performed at the same level of theory. The simulated CD spectra were compared to the experimental ones by means of the software SpecDis. Half-height peak-widths of the gaussian distributions were chosen by the software in order to best match the experimental and simulated spectra. An appropriate broadening factor σ for Gaussian curves and appropriate shift was chosen by a similarity algorithm in order to maximize the accordance between calculated and experimental spectra. All the simulated UV-Vis and CD spectra are shown in **Figure S5**.

The TD-DFT results were further analysed by means of the software TheoDORE using the Natural Transition Orbitals formalism.[5–7] The output data are shown in SI below.

Device fabrication and characterisation

Prepatterned Fraunhofer standard wafer substrates with 30nm thick gold electrodes and a 230nm thick SiO_2 dielectric layer were used to prepare chiral fullerene phototransistors with channel lengths from 2.5 to 20 μm and widths of 2 mm. The substrates were modified with HMDS through dynamic spin coating (500 rpm × 5s, 4000rpm × 55s) and baked at 120°C for 5 min. Bis[60]PCBM enantiomers at 15 mg ml^{-1} in chlorobenzene were spin coated (500 rpm x 60 s, 3000rpm × 40 s) onto the transistor substrates, followed with annealing at 120 °C for 1 hour and then annealing at 90°C under nitrogen for 3 hours. Output and transfer characteristics were measured under nitrogen using an Agilent B2900A Series Source/Measure Unit (SMU). Sensitivity of the phototransistors to unpolarised and CP light was determined using a 405 nm 50 mW diode laser (T220AX1670GD-P405150, Anford), which was polarised using a wire grid linear polariser (WP25M-VIS, Thorlabs) and a 405 nm quarter-wave plate (WPMQ05M-405, Thorlabs). A diagram of the setup is included in **Figure S12**. For continuous illumination, the intensity of this light source was controlled by varying the current supplied by a Keithley 2400 Series SMU and for temporal measurements, the laser was pulsed with an Agilent 33210A Function/Arbitrary Waveform Generator. The intensity of the light source measured using a laser power meter (LaserCheck, Coherent) at the position of the phototransistor under test.

HPLC chromatograms during purification process

The whole purification procedure for all 10 pairs of Enantiomers of bisPCBM, in total 20 enantiomers are fully listed below. All the structural isomers were fully isolated through multi-columns, including silica, 5PBB and 5PYE, which can be found in reference.[2,3] The racemates of those pure isomers were further purified by Chiralpak IE and Chiralpak IF columns here to give >99% purity.

trans-2

F2.1.2.1 & F2.1.2.2

(*S,R,* ^{f,s}*C*)49,59-bis[60]PCBM, (*R,S,* ^{f,s}*A*)49,59-bis[60]PCBM

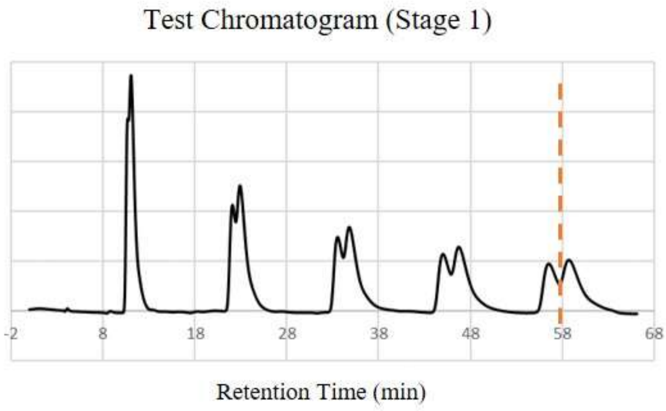

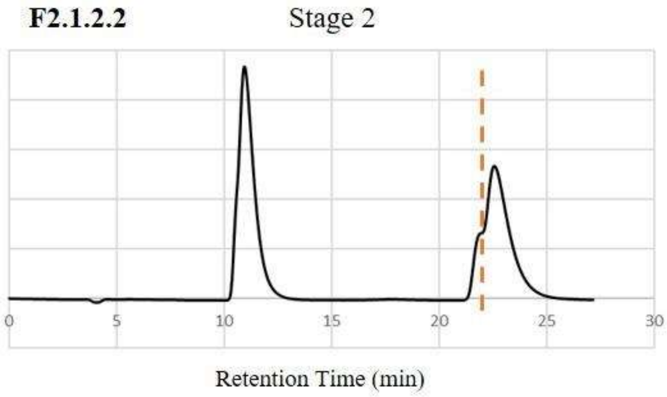

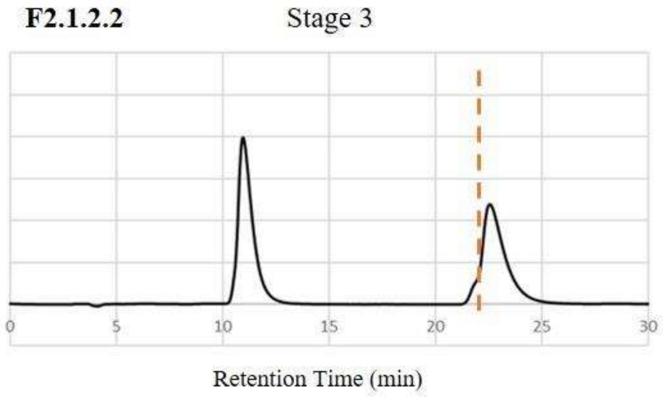

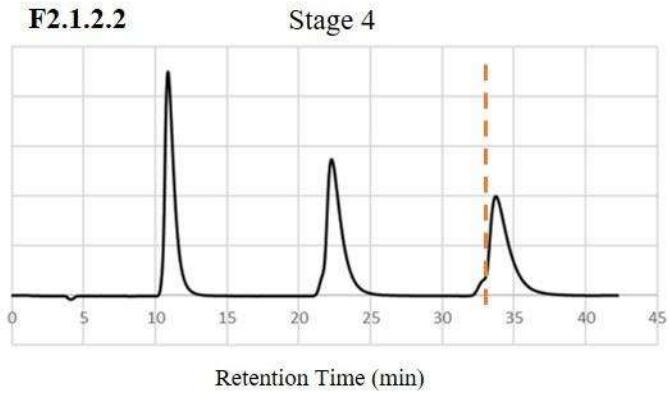

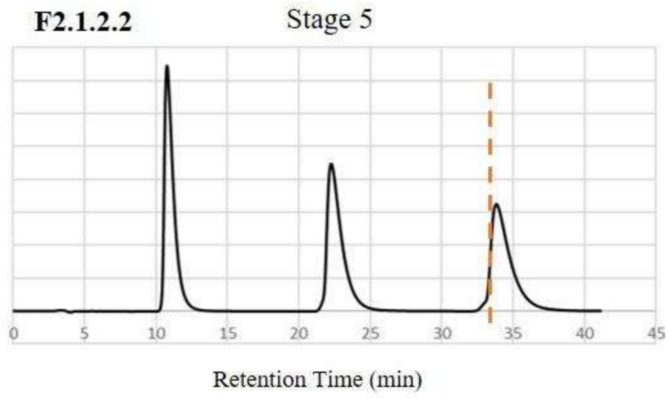

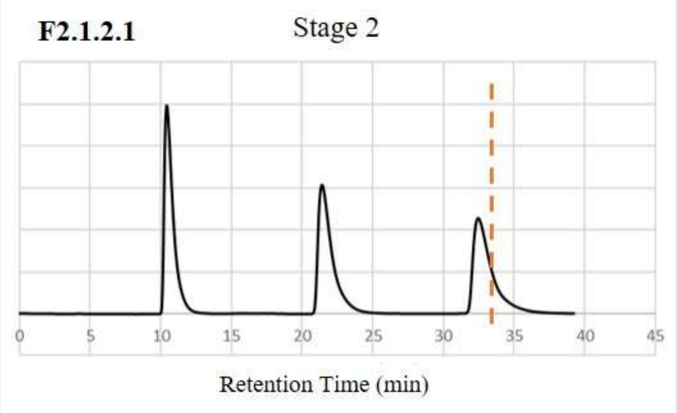

trans-2

F3.1.1 & F3.1.2

(*S*,*S*, ^{f,s}*C*)49,59-bis[60]PCBM, (*R*,*R*, ^{f,s}*A*)49,59-bis[60]PCBM

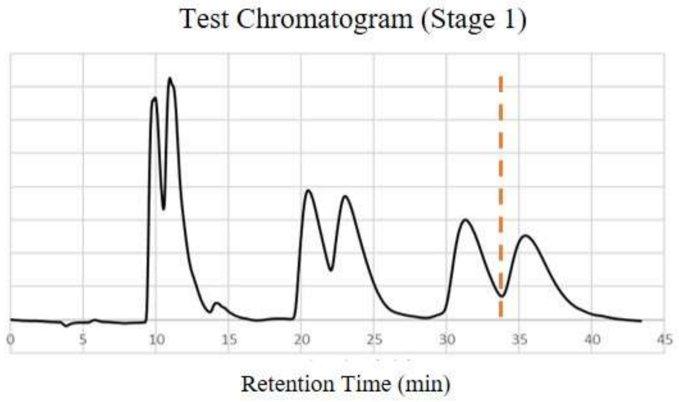

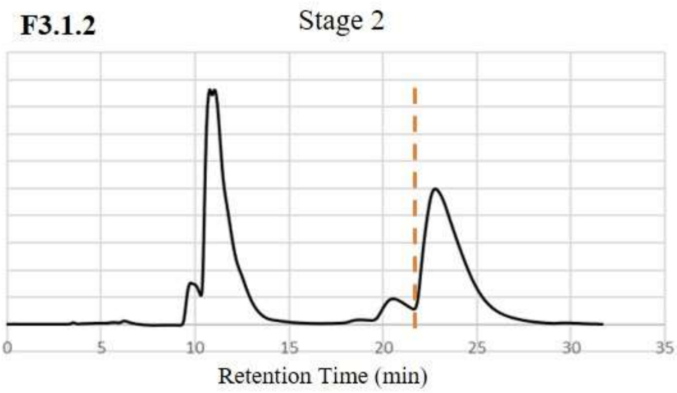

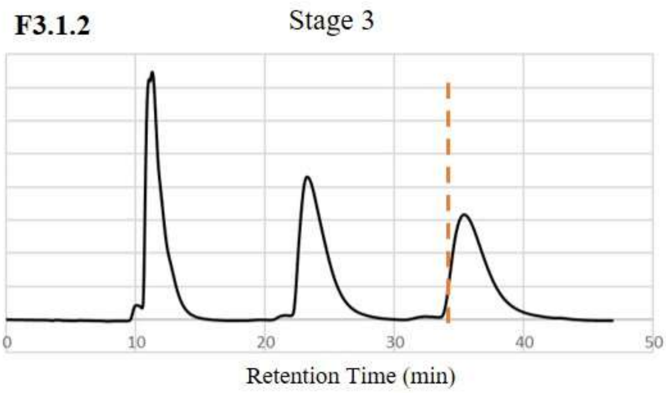

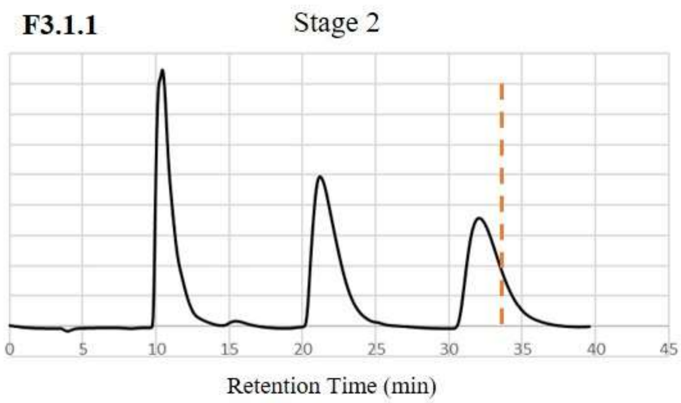

trans-3

F3.2.1.1 & F3.2.1.2

(*R*,*S*, ^{f,s}*A*)34,35-bis[60]PCBM, (*S*,*R*, ^{f,s}*C*)34,35-bis[60]PCBM

Test Chromatogram (Stage 1)

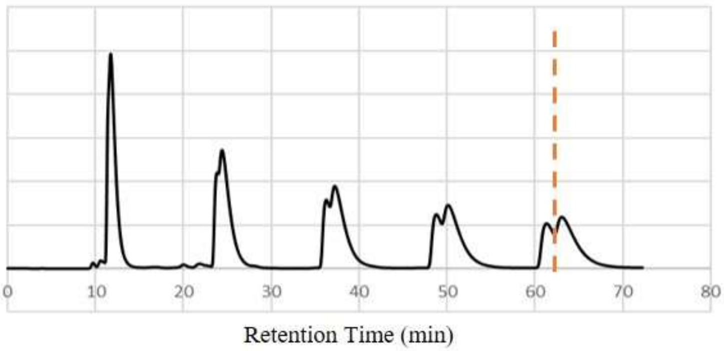

F3.2.1.2 Stage 2

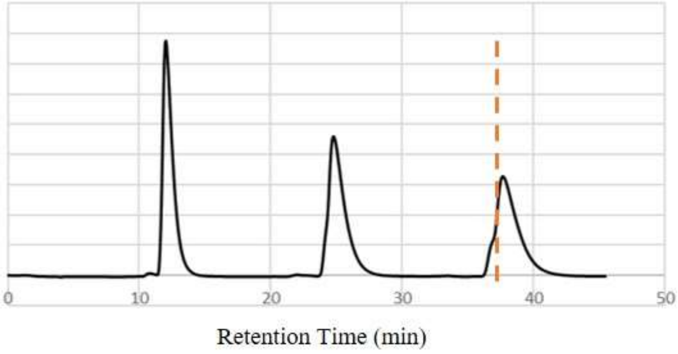

F3.2.1.2 Stage 3

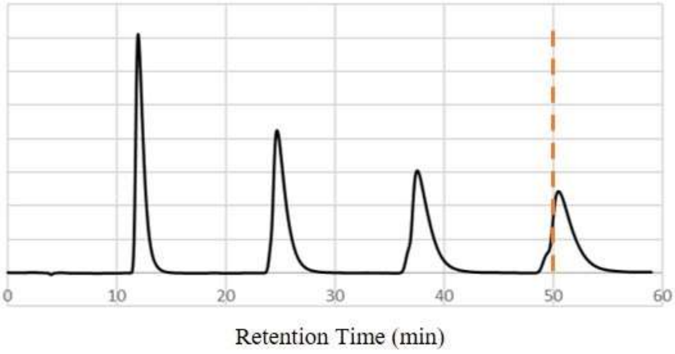

F3.2.1.2 Stage 4

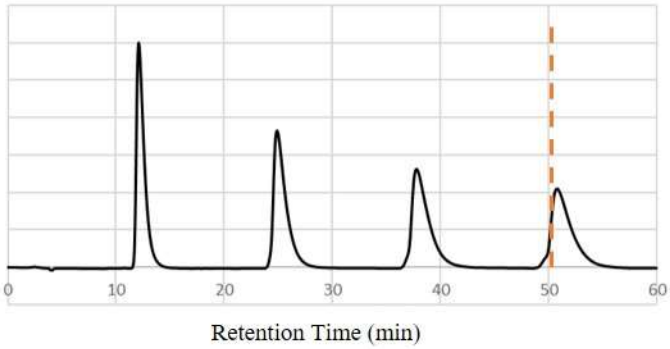

F3.2.1.2 Stage 5

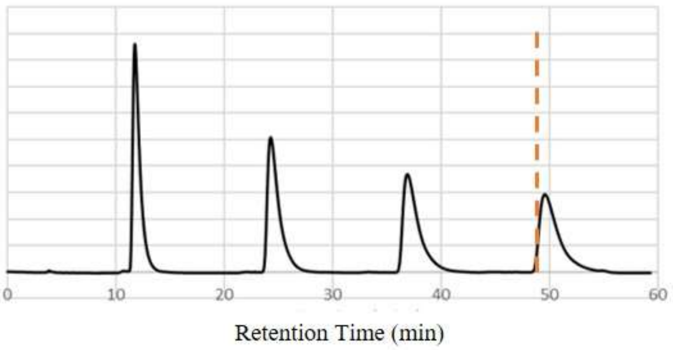

F3.2.1.1 Stage 2

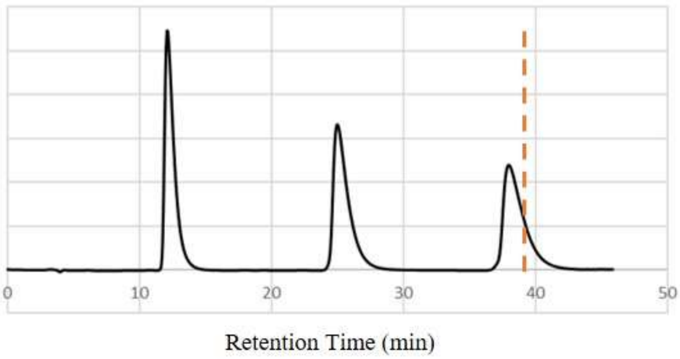

trans-3

F3.3.1.1 & F3.3.1.2

($R,R,{}^{f,s}C$)34,35 -bis[60]PCBM, ($S,S,{}^{f,s}A$)34,35-bis[60]PCBM

Test Chromatogram (Stage 1)

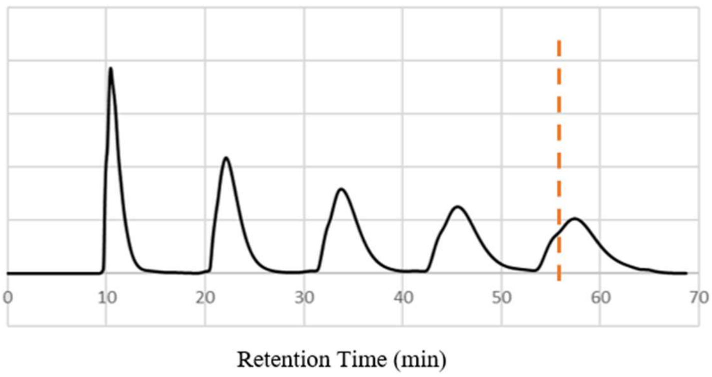

F3.3.1.2 Stage 2

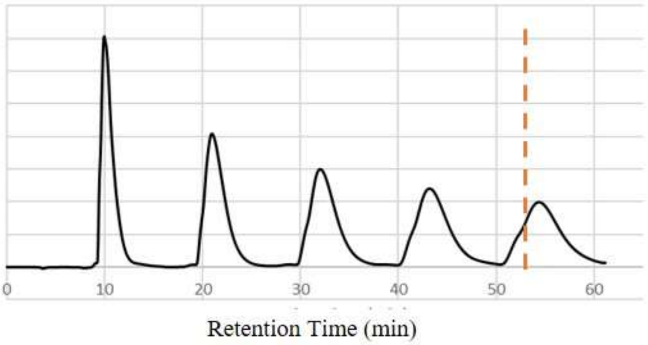

F3.3.1.2 Stage 2

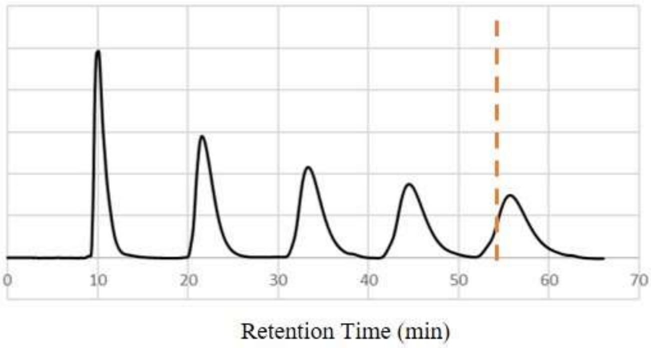

F3.3.1.2 Stage 4

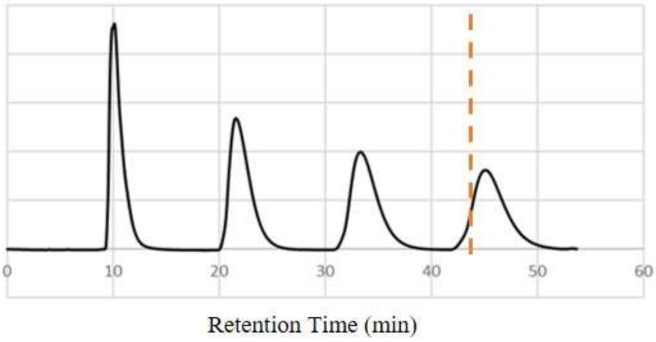

F3.3.1.2 Stage 5

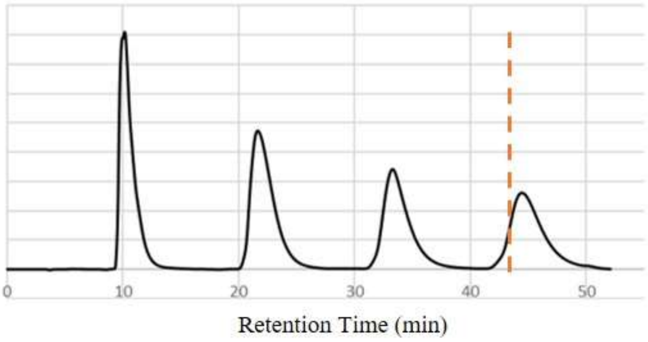

F3.3.1.1 Stage 2

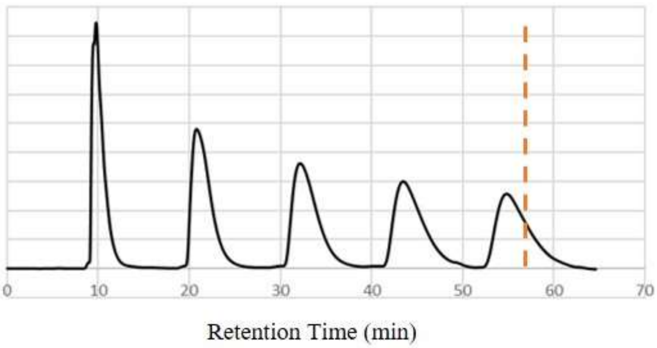

F3.3.1.1 Stage 3

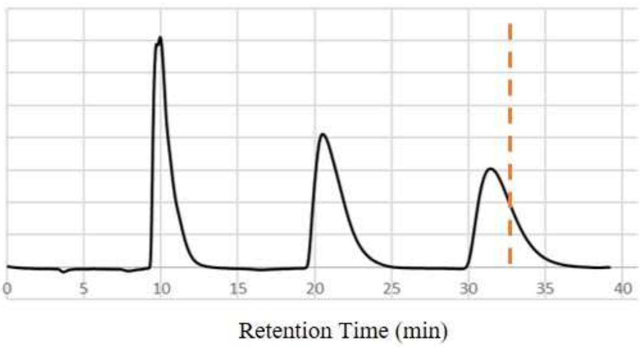

trans-3

F2.3.1 & F2.3.2

(R,R, $^{f,s}A$)34,35-bis[60]PCBM, (S,S, $^{f,s}C$)34,35-bis[60]PCBM

Test Chromatogram (Stage 1)

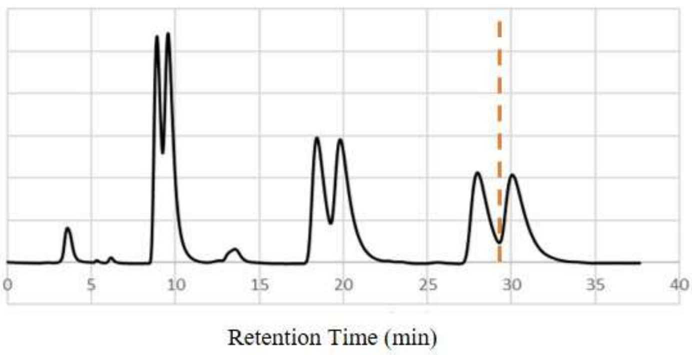

F2.3.2 Stage 2

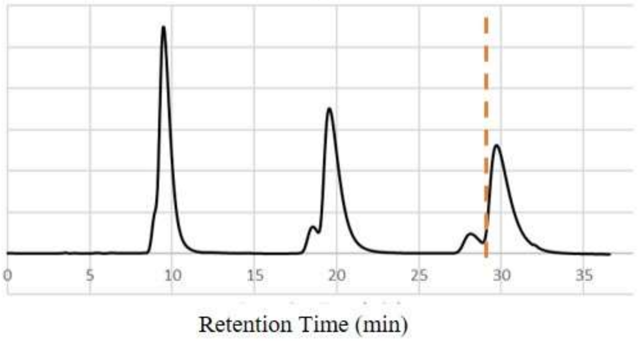

F2.3.2 Stage 3

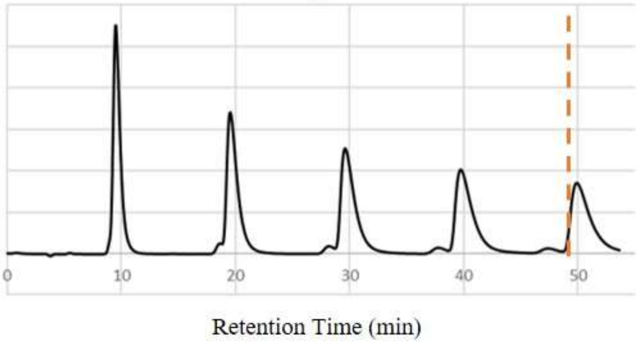

F2.3.1 Stage 2

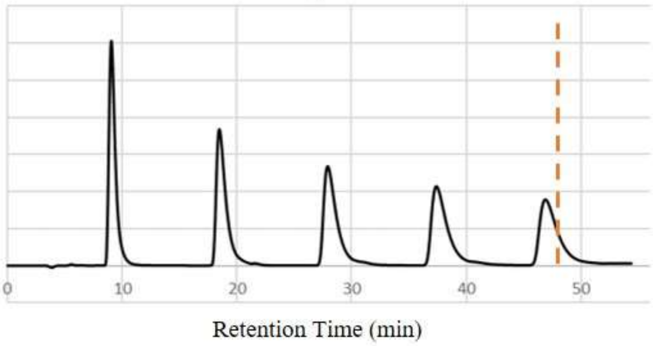

trans-4

F3.2.2.1 & F3.2.2.2

(*S,S*)32,33-bis[60]PCBM, (*R,R*)32,33-bis[60]PCBM

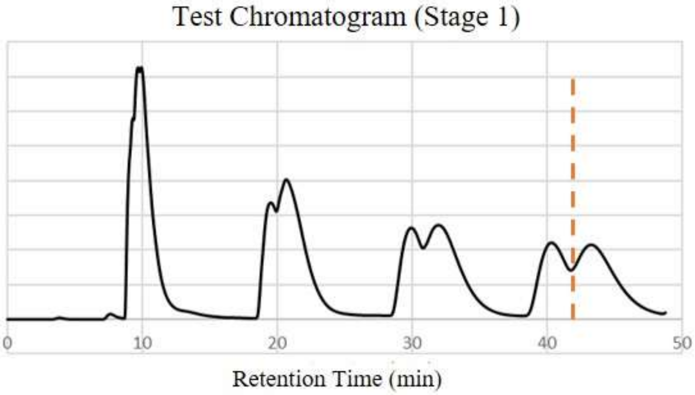

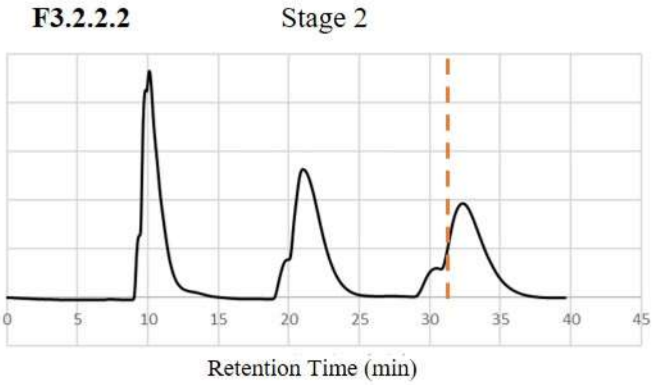

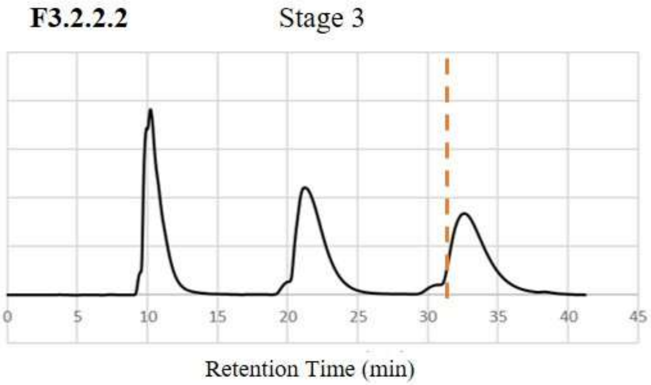

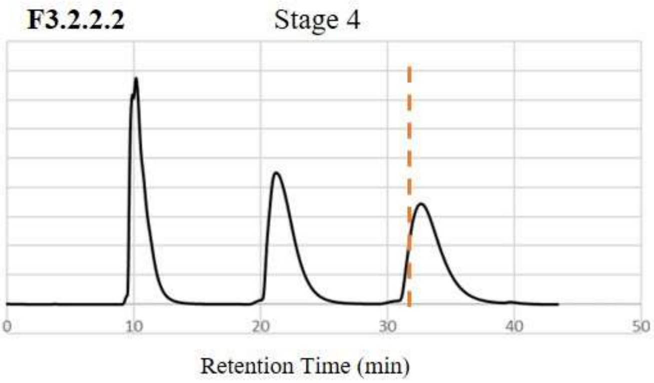

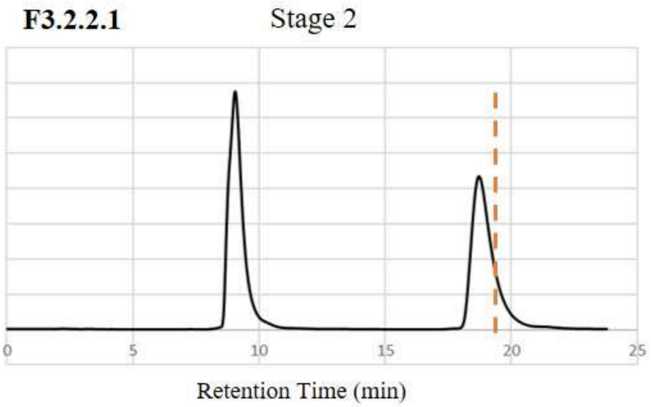

cis-3

F3.4.1 & F3.4.2

(*S,S,*^{f,s}*C*)13,14-bis[60]PCBM, (*R,R,*^{f,s}*A*)13,14-bis[60]PCBM

Test Chromatogram (Stage 1)

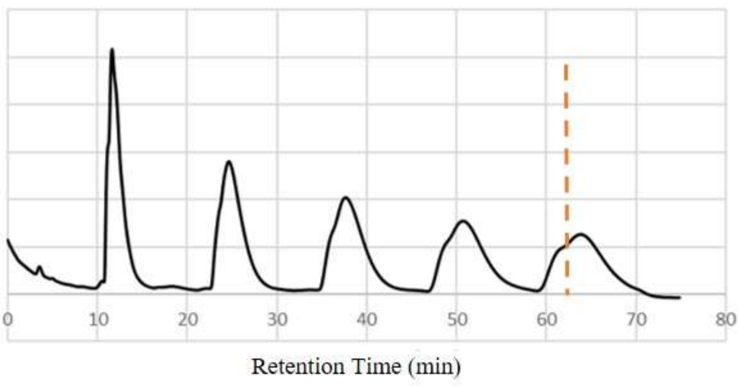

F3.4.2 Stage 2

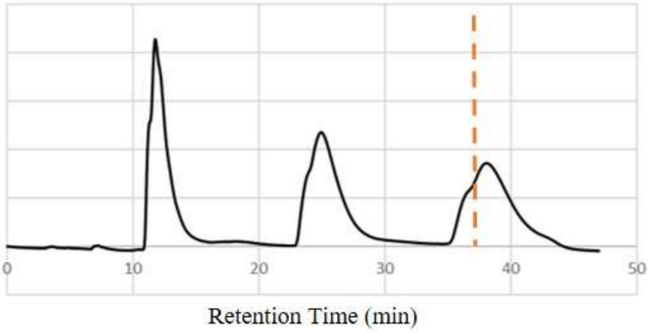

F3.4.2 Stage 3

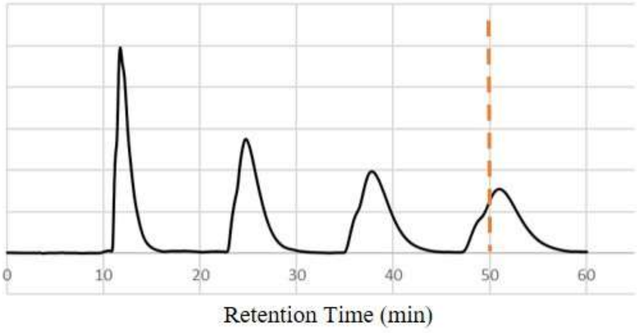

F3.4.2 Stage 4

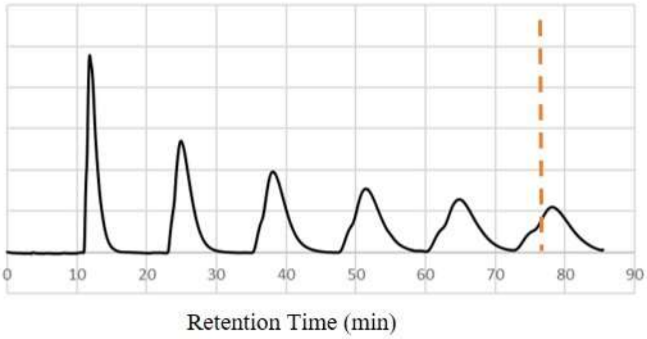

F3.4.2 Stage 5

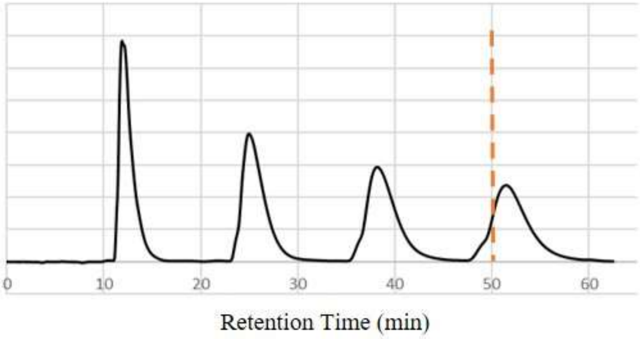

F3.4.2 Stage 6

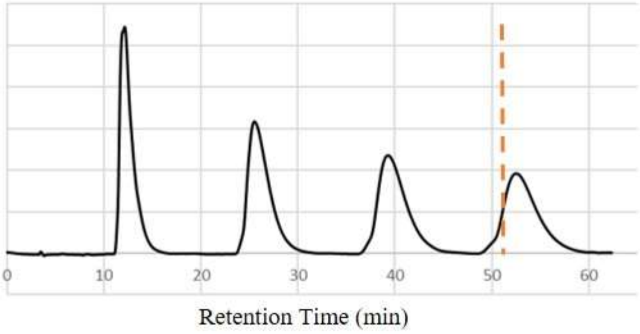

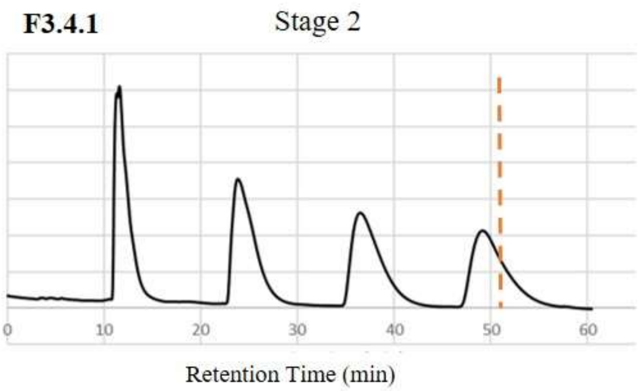

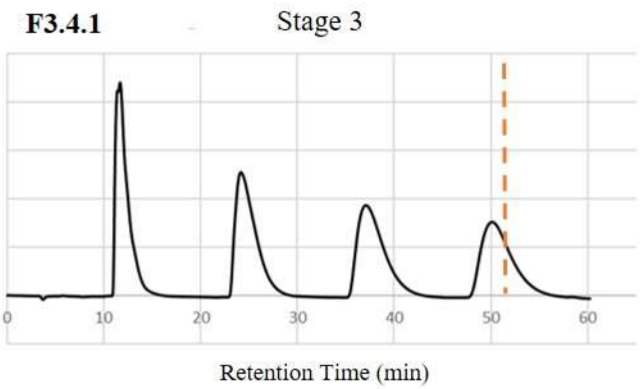

cis-3

F5.2.2.1 & F5.2.2.2

(*S,R,* ^{f,s}*A*)13,14-bis[60]PCBM, (*R,S,* ^{f,s}*C*)13,14-bis[60]PCBM

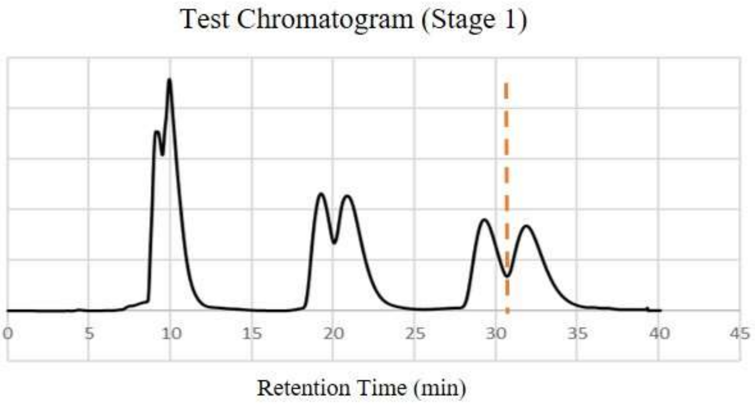

F5.2.2.2 Stage 2

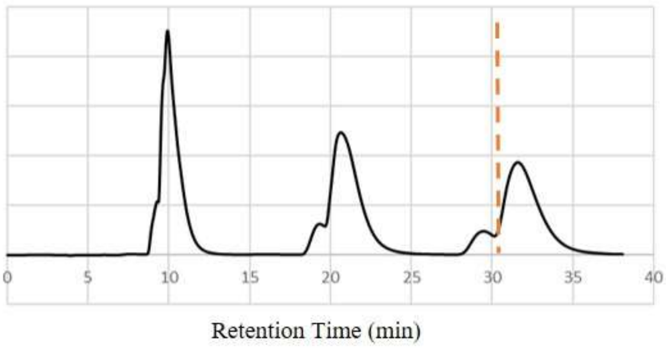

F5.2.2.2 Stage 3

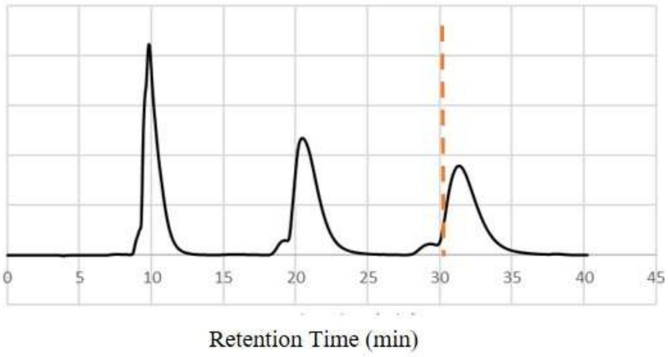

F5.2.2.2 Stage 4

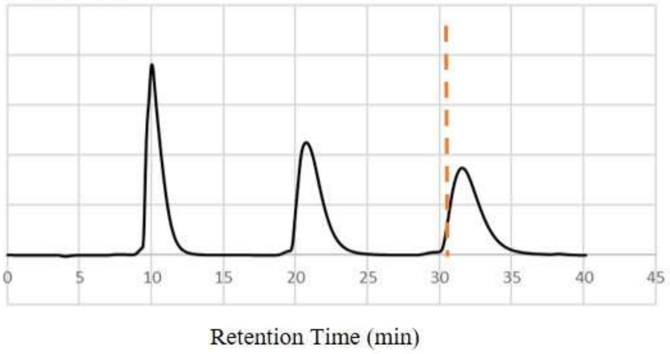

F5.2.2.1 Stage 2

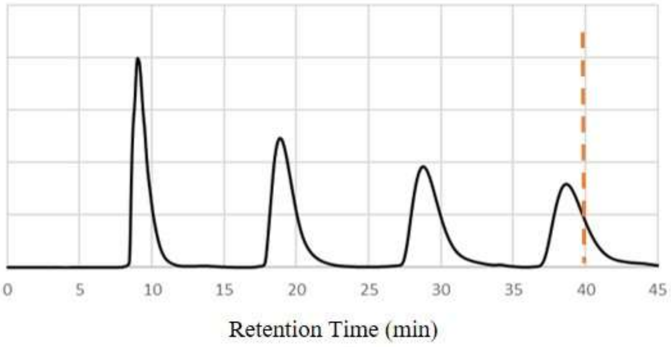

e

F5.1.1 & F5.1.2

(*anti,R*)16,17-bis[60]PCBM, (*anti,S*)16,17-bis[60]PCBM

Test Chromatogram (Stage 1)

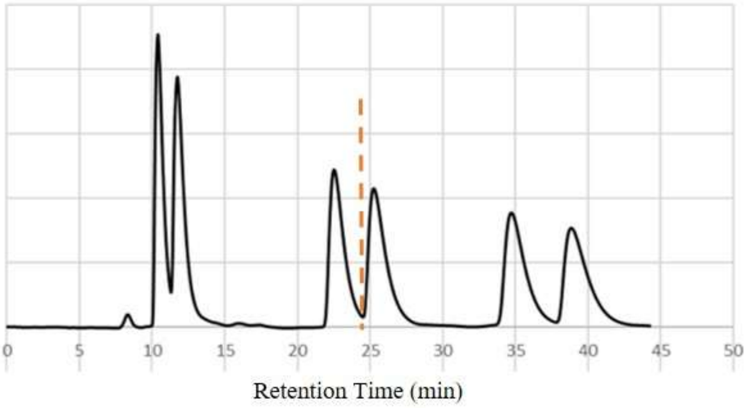

F5.1.2 Stage 2

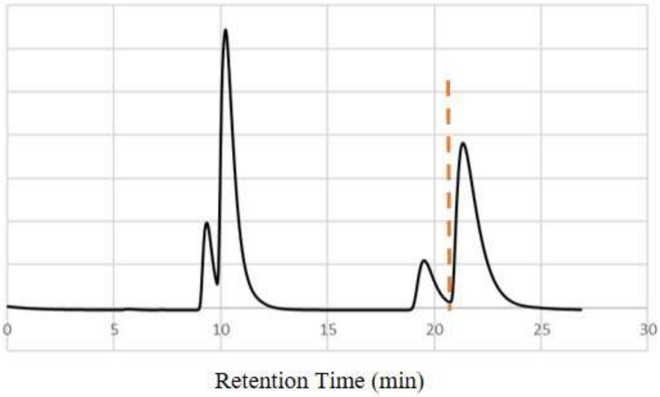

F5.1.2 Stage 3

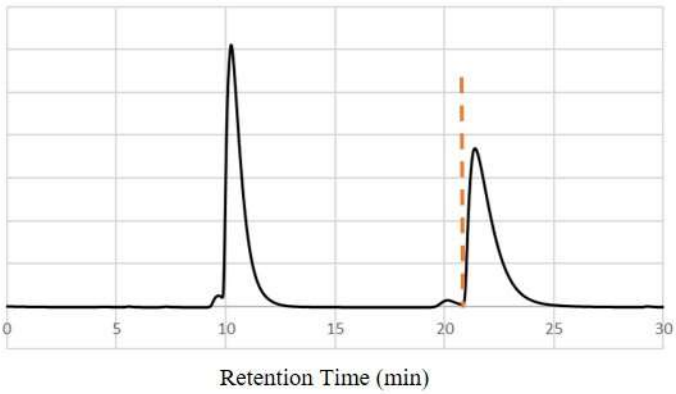

F5.1.2 Stage 4

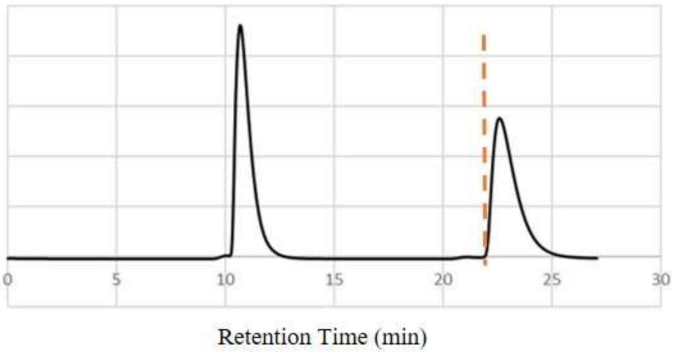

F5.1.1 Stage 2

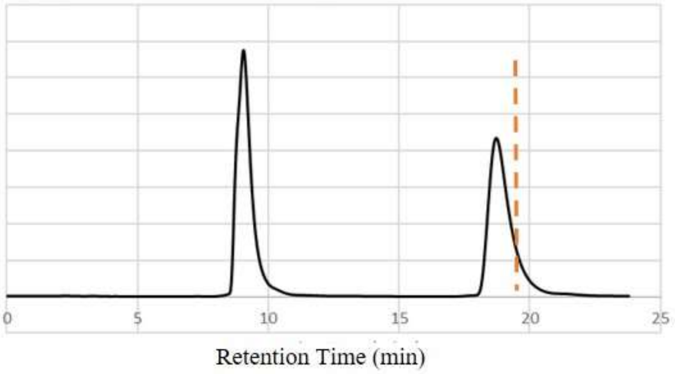

cis-2

F6.1 & F6.2

(*S,S*)3,15-bis[60]PCBM, (*R,R*)3,15-bis[60]PCBM

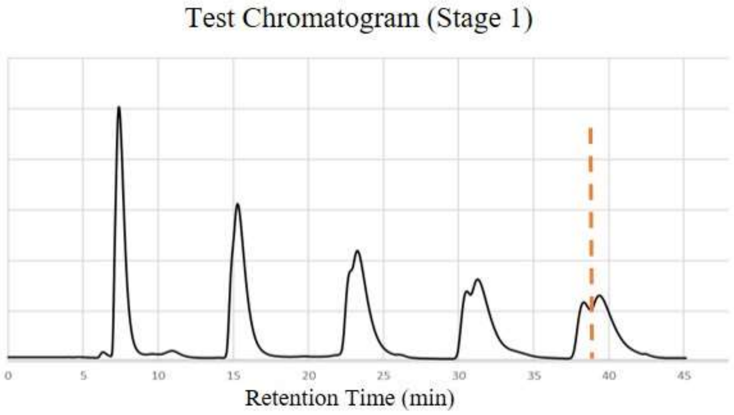

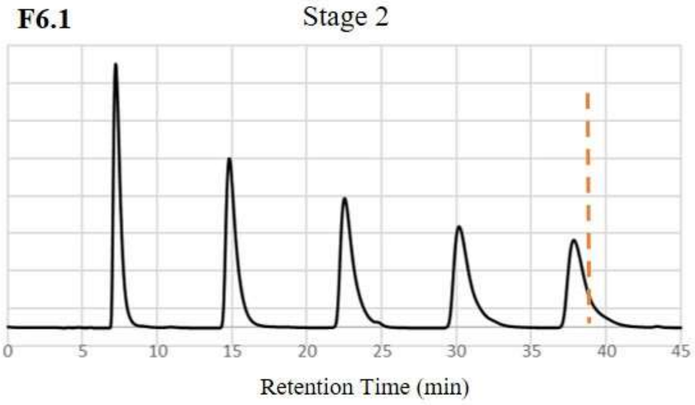

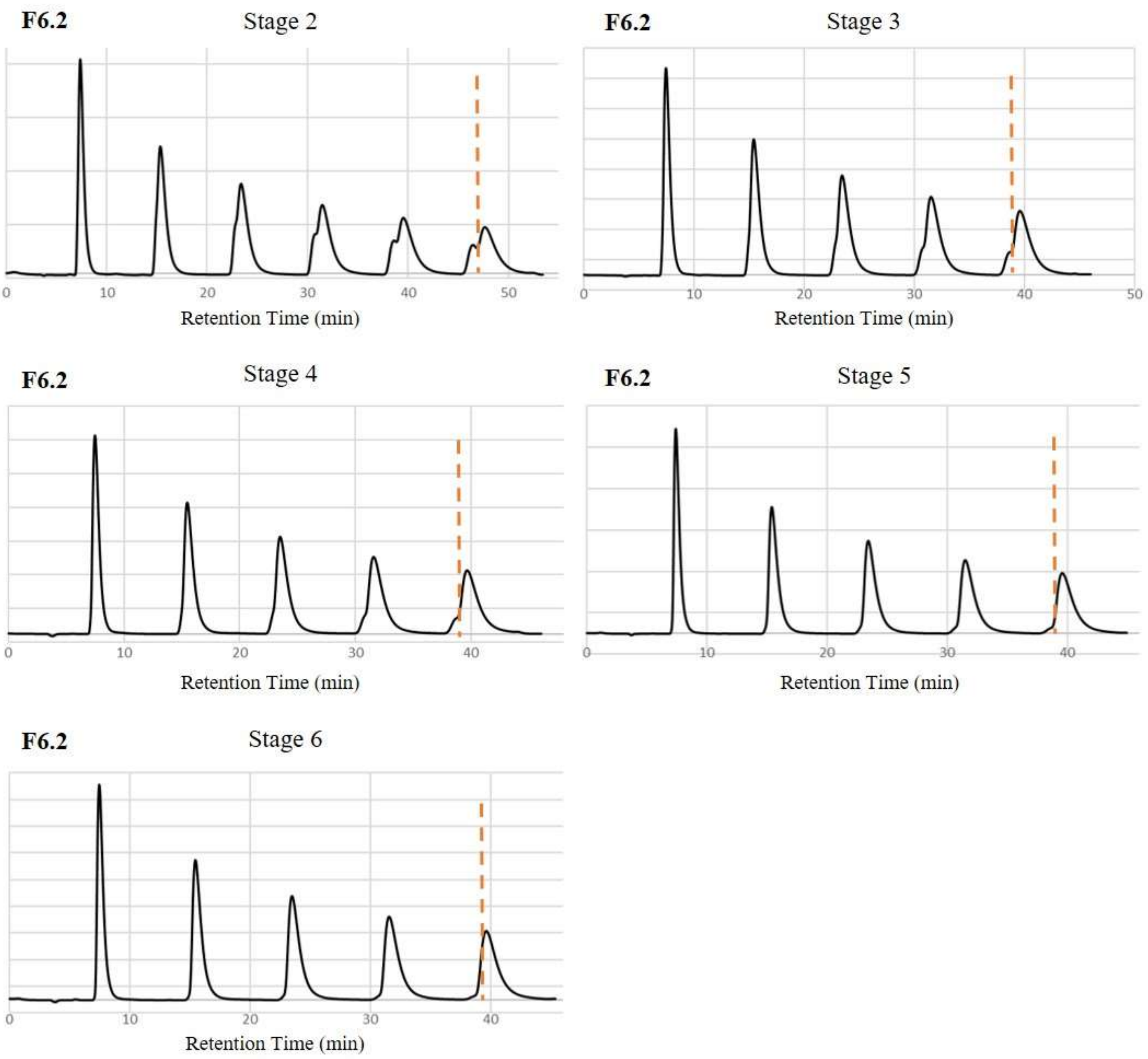

Figure S1 chiral HPLC traces of the 10 resolved pairs of enantiomers.

Absolute configuration assignment

Table S1. Complete structural assignment according to the combined analysis of simulated CD and previous bis[60]PCBM regioisomers assignment. The stereoisomers are ordered by HPLC fractions order, bond type and symmetry.

HPLC Fraction	Enantiomer A	HPLC Fraction	Enantiomer B	Bond Type	Symmetry
F6.1	(*S,S*)3,15-bis[60]PCBM	F6.2	(*R,R*)3,15-bis[60]PCBM	*cis-2*	C_1
F5.2.2.1	(*S,R,* ^{f,s}*A*)13,14-bis[60]PCBM	F5.2.2.2	(*R,S,* ^{f,s}*C*)13,14-bis[60]PCBM	*cis-3*	C_1
F3.4.1	(*S,S,* ^{f,s}*C*)13,14-bis[60]PCBM	F3.4.2	(*R,R,* ^{f,s}*A*)13,14-bis[60]PCBM	*cis-3*	C_2
F5.1.1	(*anti,R*)16,17-bis[60]PCBM	F5.1.2	(*anti,S*)16,17-bis[60]PCBM	*e*	C_1
F3.2.2.1	(*S,S*)32,33-bis[60]PCBM	F3.2.2.2	(*R,R*)32,33-bis[60]PCBM	*trans-4*	C_1
F2.3.1	(*R,R,* ^{f,s}*A*)34,35-bis[60]PCBM	F2.3.2	(*S,S,* ^{f,s}*C*)34,35-bis[60]PCBM	*trans-3*	C_2
F3.3.1.1	(*R,R,* ^{f,s}*C*)34,35 -bis[60]PCBM	F3.3.1.2	(*S,S,* ^{f,s}*A*)34,35-bis[60]PCBM	*trans-3*	C_2
F3.2.1.1	(*R,S,* ^{f,s}*A*)34,35-bis[60]PCBM	F3.2.1.2	(*S,R,* ^{f,s}*C*)34,35-bis[60]PCBM	*trans-3*	C_1
F3.1.1	(*S,S,* ^{f,s}*C*)49,59-bis[60]PCBM	F3.1.2	(*R,R,* ^{f,s}*A*)49,59-bis[60]PCBM	*trans-2*	C_2
F2.1.2.1	(*S,R,* ^{f,s}*C*)49,59-bis[60]PCBM	F2.1.2.2	(*R,S,* ^{f,s}*A*)49,59-bis[60]PCBM	*trans-2*	C_1

Note: Enantiomer A and B in a row are in pair. In total, ten pairs of enantiomers are listed here.

Spectroscopic Characterization

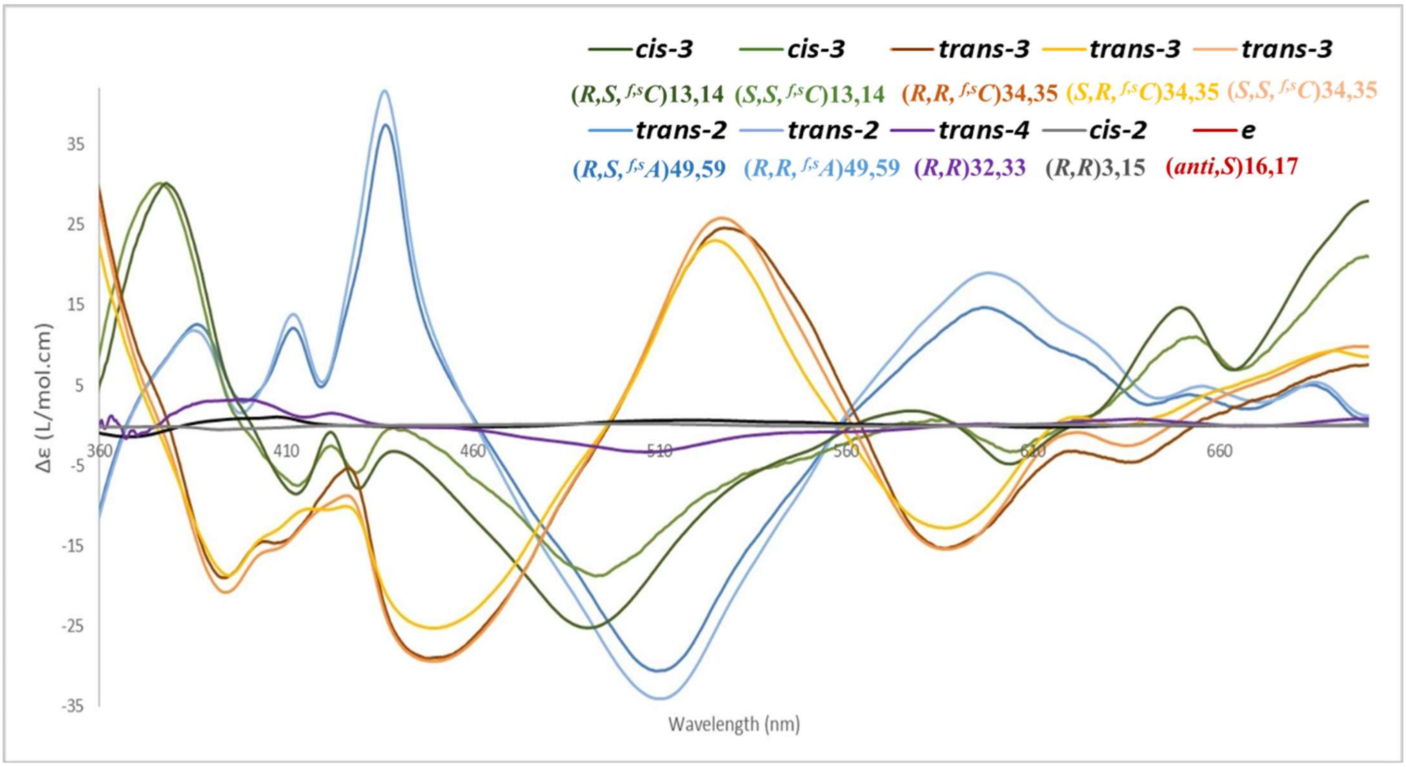

Figure S2 Normalised CD spectra of representative enantiomer for all 10 pairs studied.

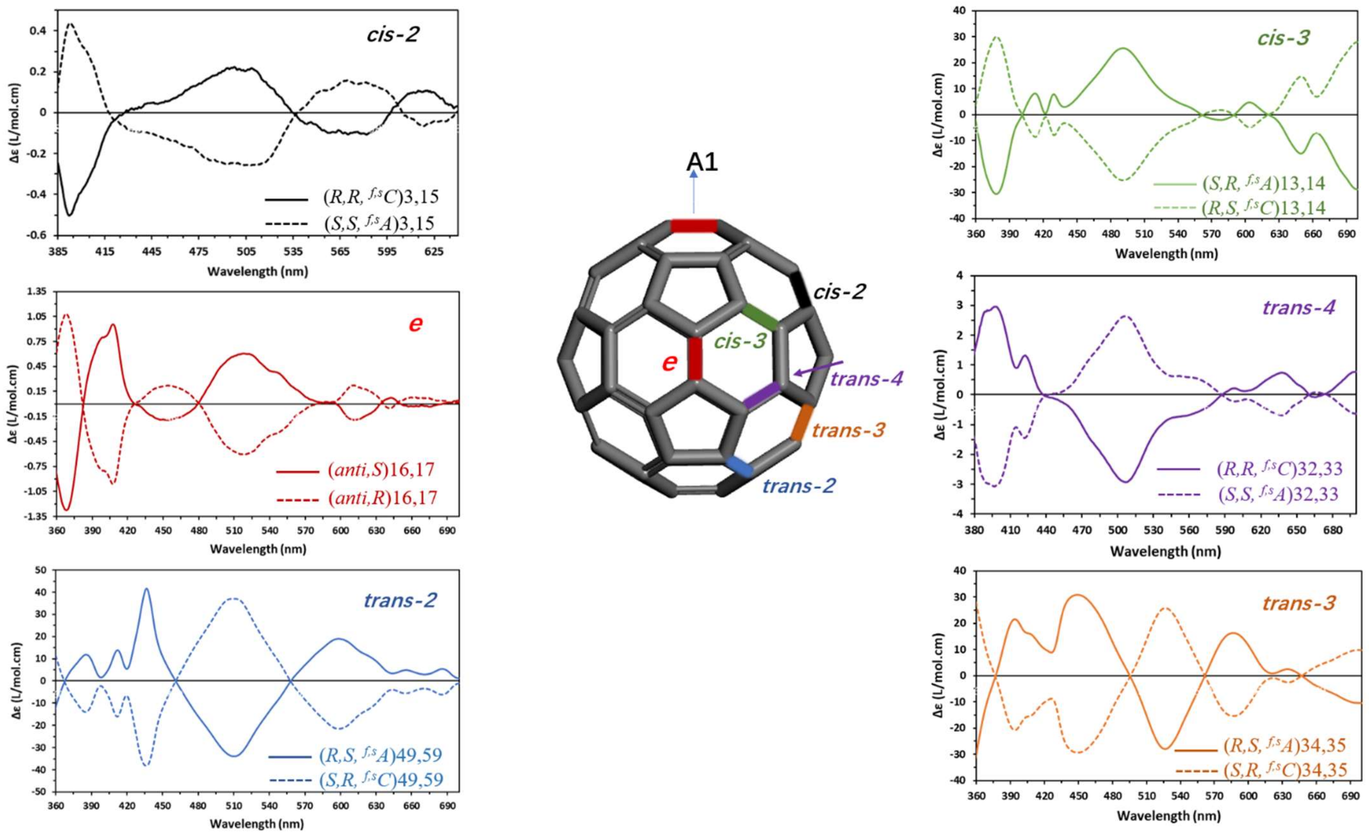

Figure S3 Experimental normalised CD spectra of one representative enantiomeric pair per substitution pattern; continuous line for $^{f,s}A$ and dotted line for $^{f,s}C$ configuration. Common to all bond types seems to be the region of strong absorption between 480 and 540 nm which can be used to establish a general rule for the absolute configuration. A positive band in this region corresponds to the $^{f,s}C$ configuration for the *trans-2* and *trans-3*, the $^{f,s}A$ for the *cis-3* addition patterns, respectively. A positive band in the same region also corresponds to the *S* configuration for the *e* and *trans-4* and the *R* for the *cis-2* addition patterns, respectively.

6.1 blue (*S,S*)3,15-bis[60]PCBM
6.2 orange (*R,R*)3,15-bis[60]PCBM

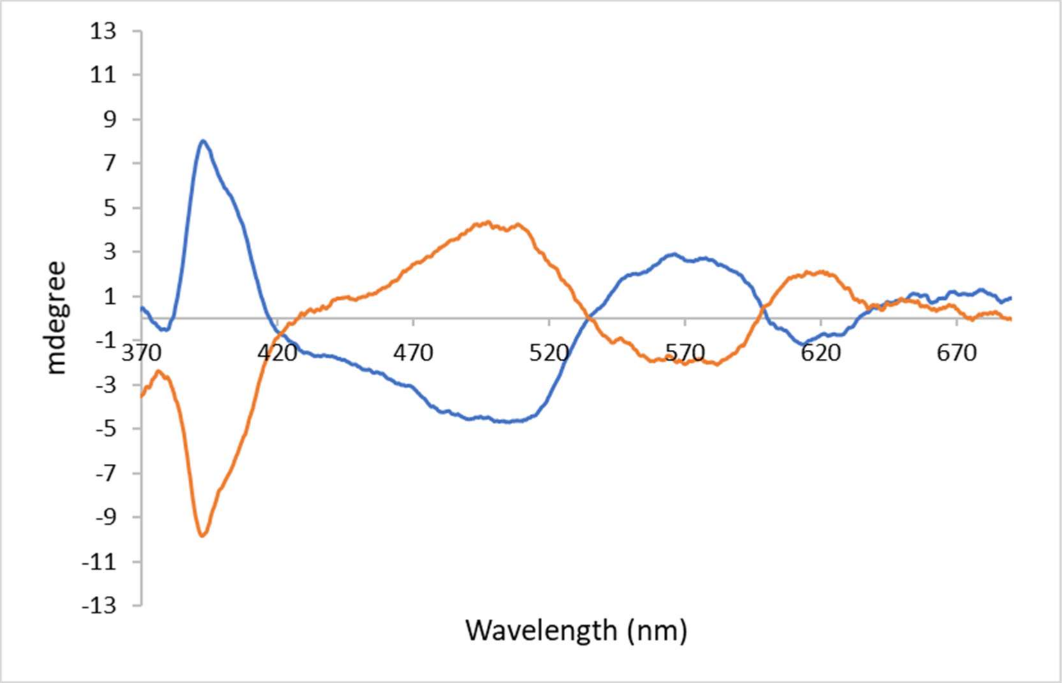

3.4.1 orange (*S*,*S*, *f,sC*)13,14-bis[60]PCBM
3.4.2 blue (*R*,*R*, *f,sA*)13,14-bis[60]PCBM

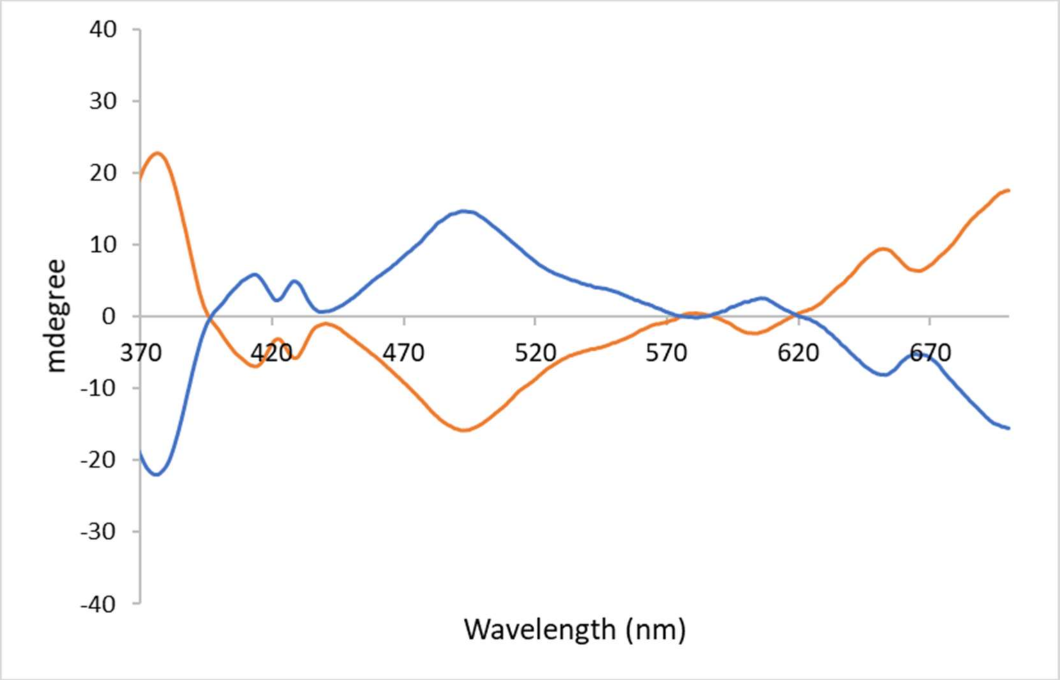

5.2.2.1 blue (*S*,*R*, *f,sA*)13,14-bis[60]PCBM
5.2.2.2 orange (*R*,*S*, *f,sC*)13,14-bis[60]PCBM

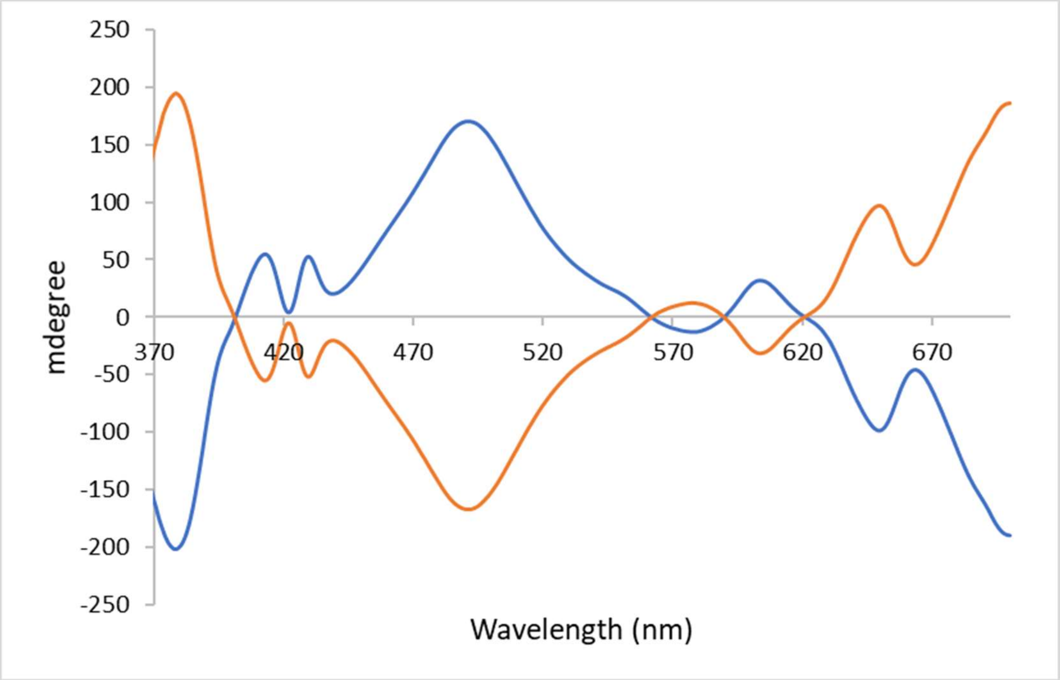

5.1.1 orange (*anti,R*)16,17-bis[60]PCBM
5.1.2 blue (*anti,S*)16,17-bis[60]PCBM

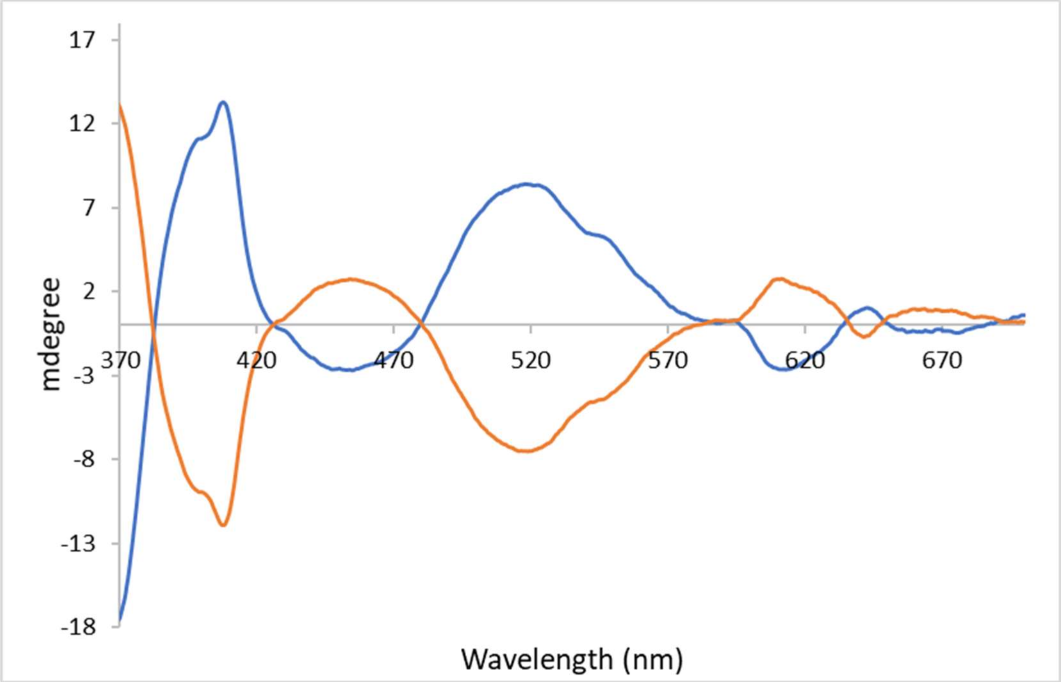

3.2.2.1 blue (*S,S*)32,33-bis[60]PCBM
3.2.2.2 orange (*R,R*)32,33-bis[60]PCBM

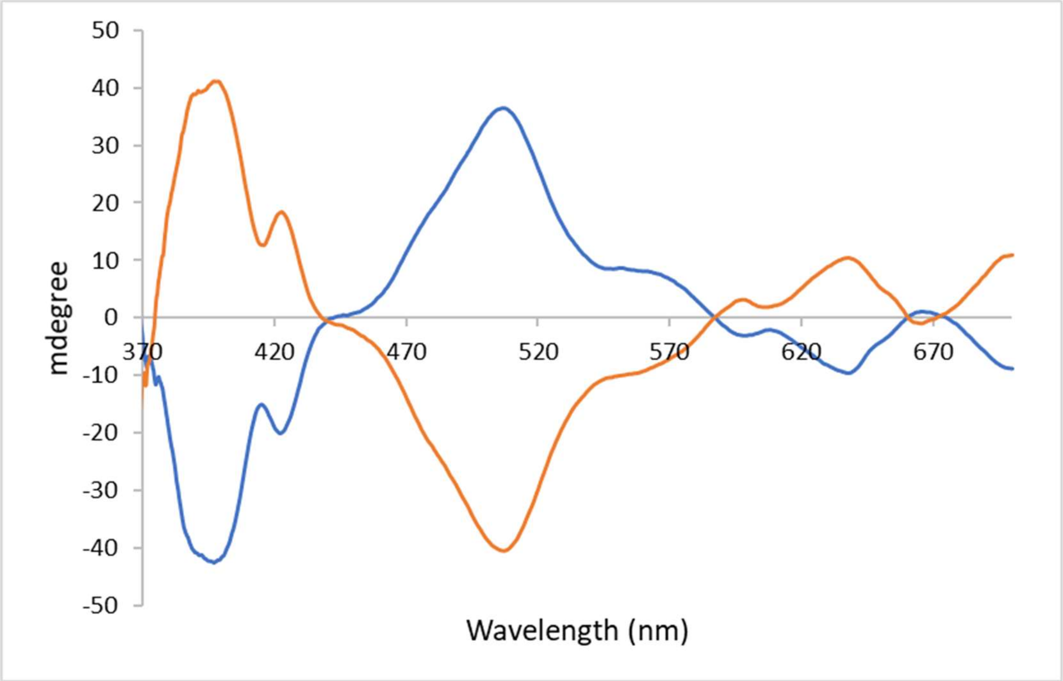

2.3.1 blue (*R*,*R*, ^{f,s}*A*)34,35-bis[60]PCBM
2.3.2 orange (*S*,*S*, ^{f,s}*C*)34,35-bis[60]PCBM

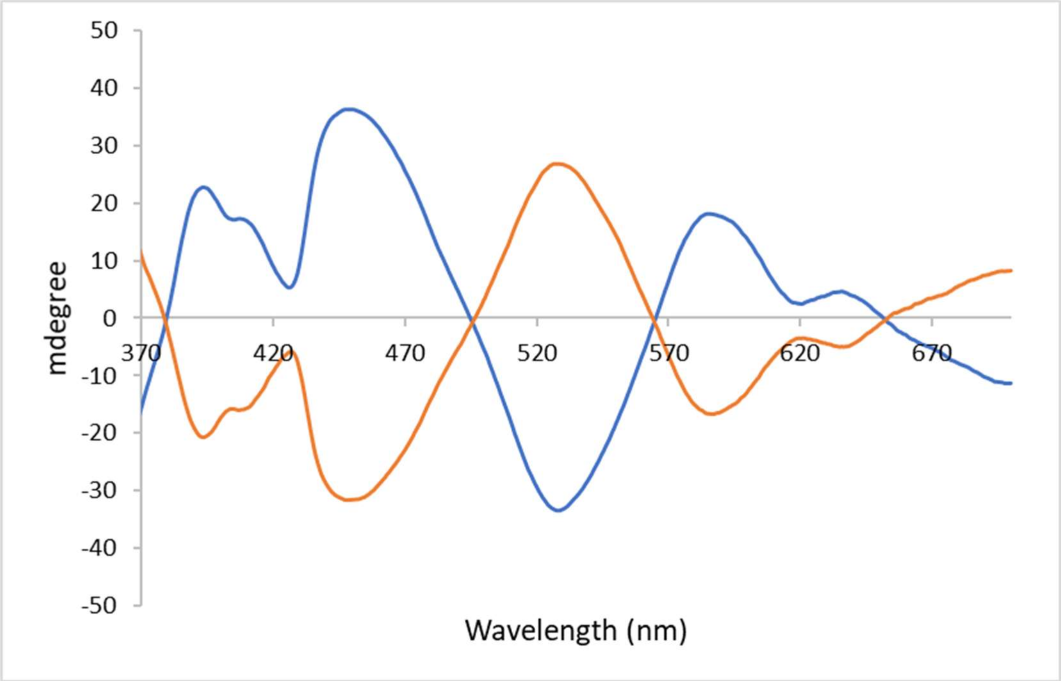

3.2.1.1 blue (*R*,*S*, ^{f,s}*A*)34,35-bis[60]PCBM
3.2.1.2 orange (*S*,*R*, ^{f,s}*C*)34,35-bis[60]PCBM

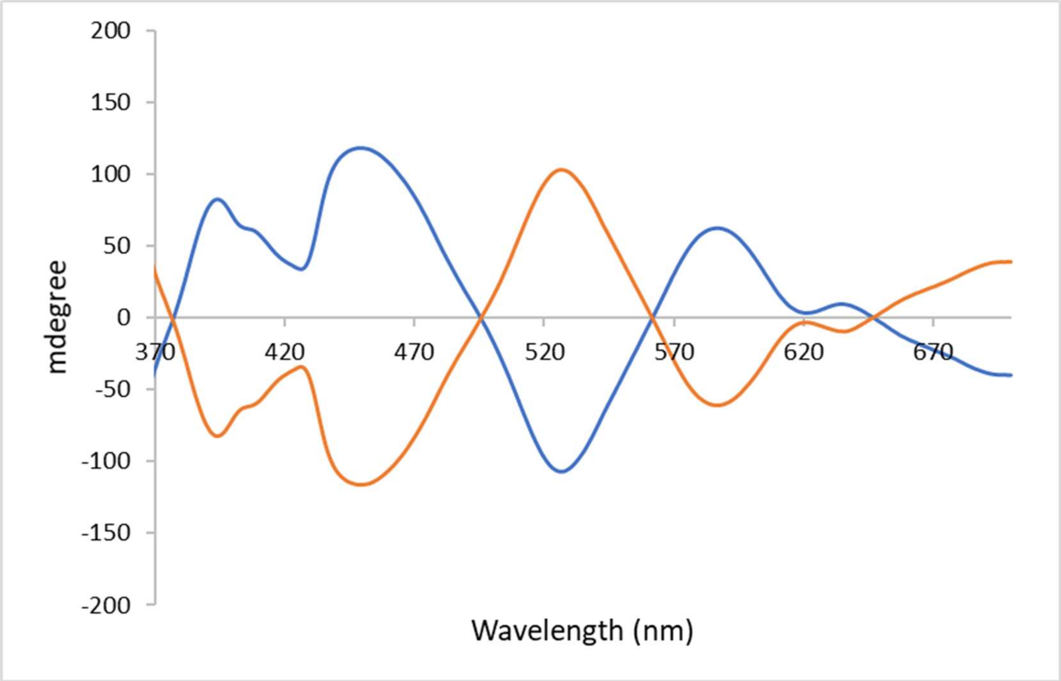

3.3.1.1 orange (*R*,*R*, *f,s*C)34,35-bis[60]PCBM
3.3.1.2 blue (*S*,*S*, *f,s*A)34,35 -bis[60]PCBM

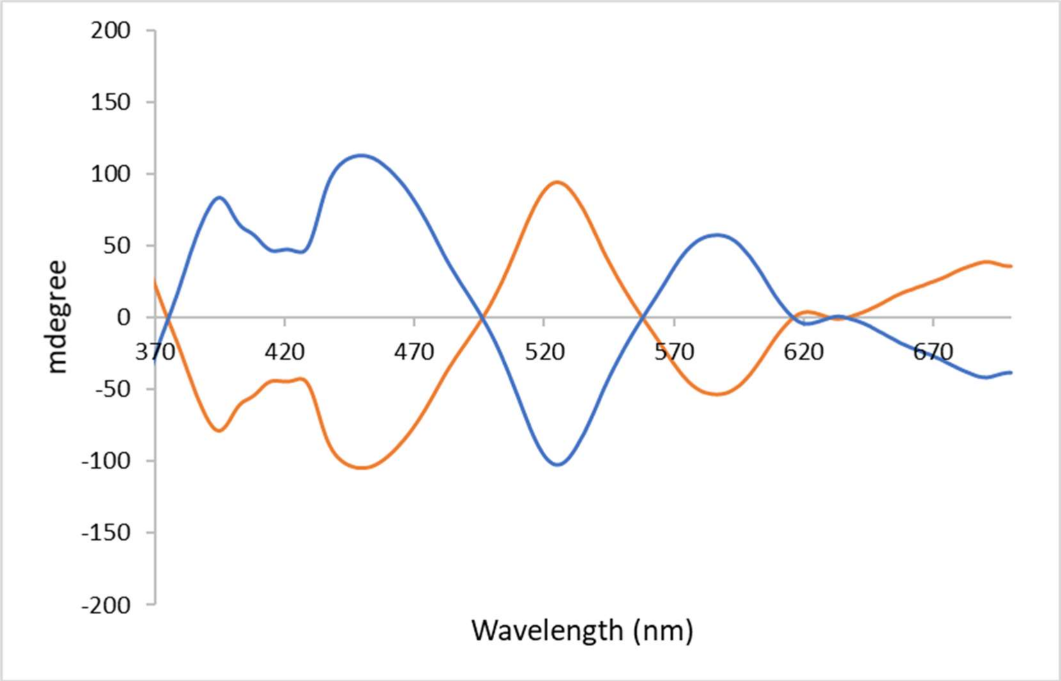

2.1.2.1 orange (*S*,*R*, *f,s*C)49,59-bis[60]PCBM
2.1.2.2 blue (*R*,*S*, *f,s*A)49,59-bis[60]PCBM

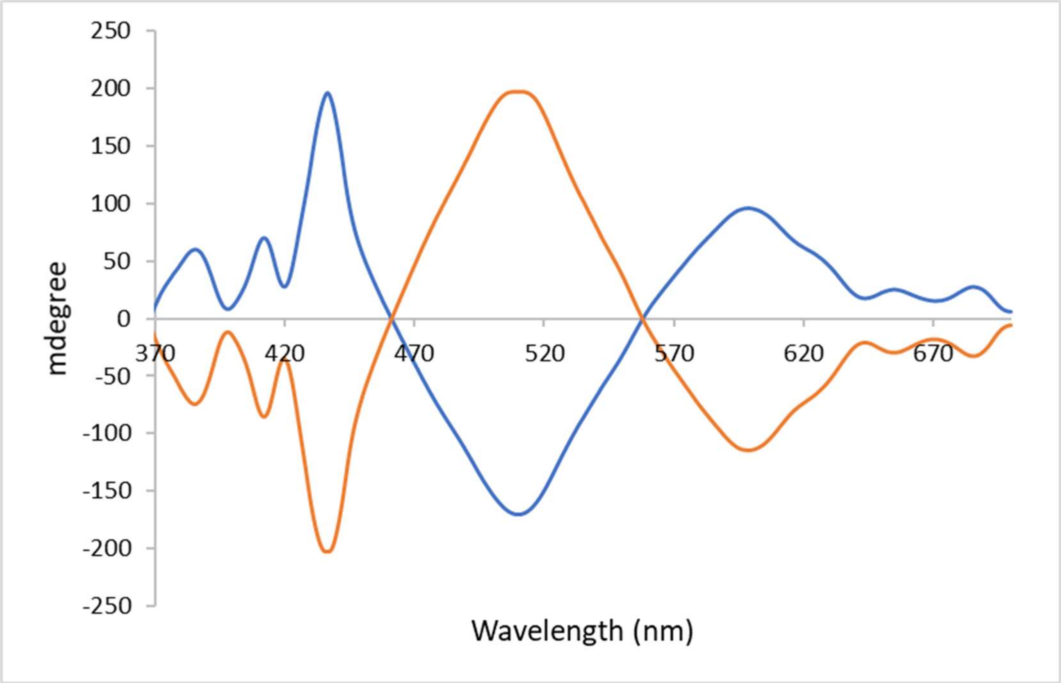

3.1.1 orange (*S*,*S*, ${}^{f,s}C$)49,59-bis[60]PCBM
3.1.2 blue (*R*,*R*, ${}^{f,s}A$)49,59-bis[60]PCBM

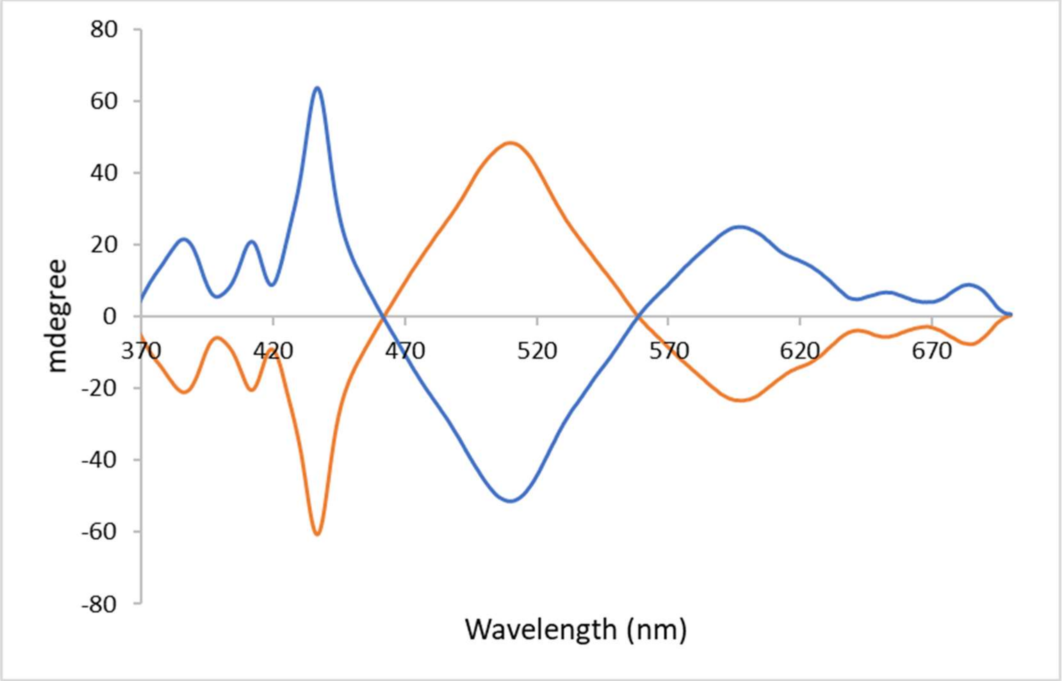

Figure S4 CD spectra measured for all 10 pairs of enantiomers studied.

Description of the nomenclature system

Due to the I_h symmetry, C_{60} has six equivalent C_5 axes passing through opposite pentagons. From the atom of the pentagon cut by the C_5 axis used as reference axis is possible to numerate the whole molecule describing spiral pathways in either direction. This numbering is called *systematic.* The first addend is by definition positioned on the bond 1,9. Therefore, 1,9 in the nomenclature is always omitted. The nomenclature is then chosen giving priority to the lowest possible number to the position of the second addend. If the lowest possible number can be achieved for the numbering, the configuration is $^{f,s}C$, where the apex stand for *fullerene* and *systematic* and *C* for *clockwise*. In case the lowest possible number can be achieved by numbering mirrored to the figure, the configuration is called $^{f,s}A$ (*anti-clockwise*).

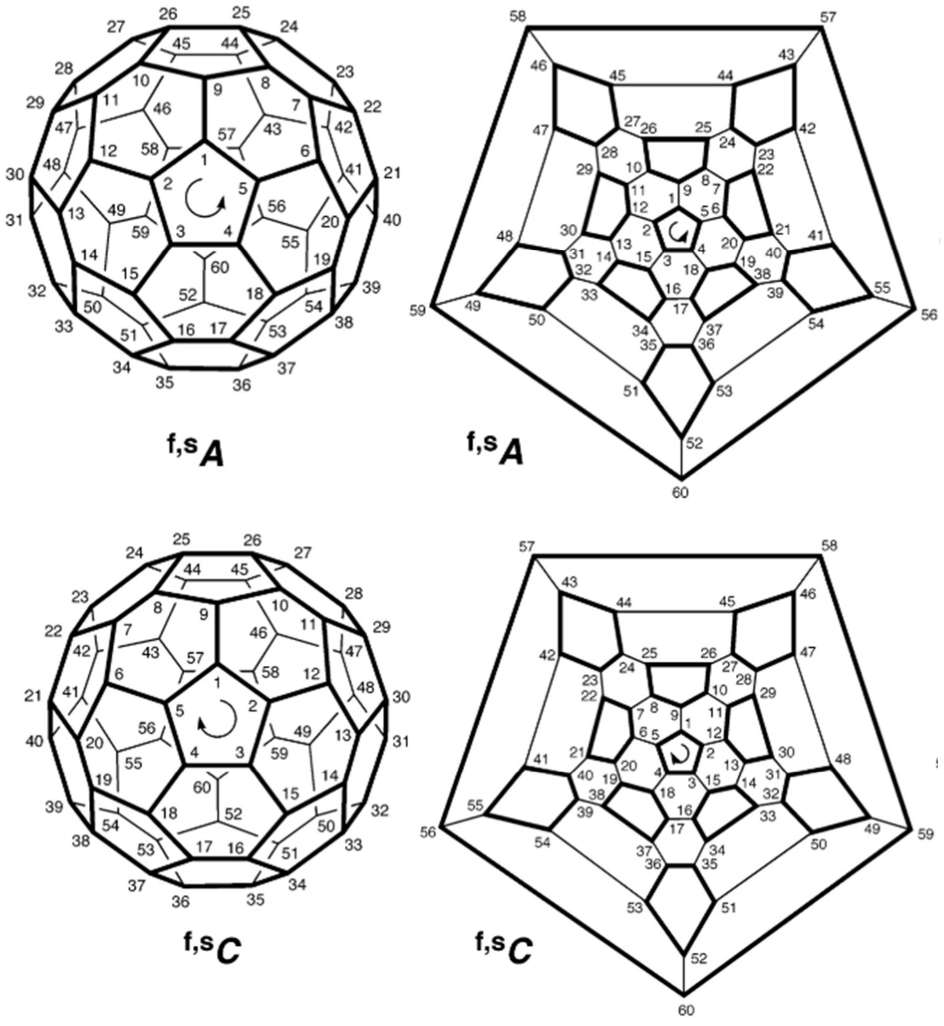

For example, the isomer below shows the lowest numbering for the *clockwise* configuration (notice from the figure above that the *anticlockwise* numbering would be incorrect, since the second added would be on the 19,20 bond, which is higher than 13,14).

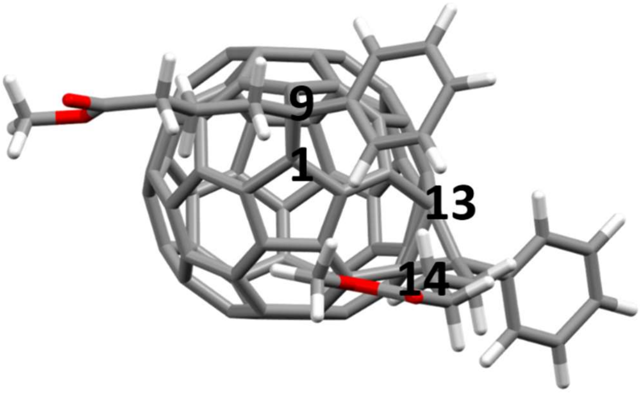

To this, we need to add the descriptors R/S for the addends orientation. These are given to the quaternary carbon of the substituents according to the standard stereochemistry Cahn-Ingold-Prelog (CIP) priority rules.[8] The lowest priority is always given to the methyl butyric ester, and second lowest priority to the phenyl group.

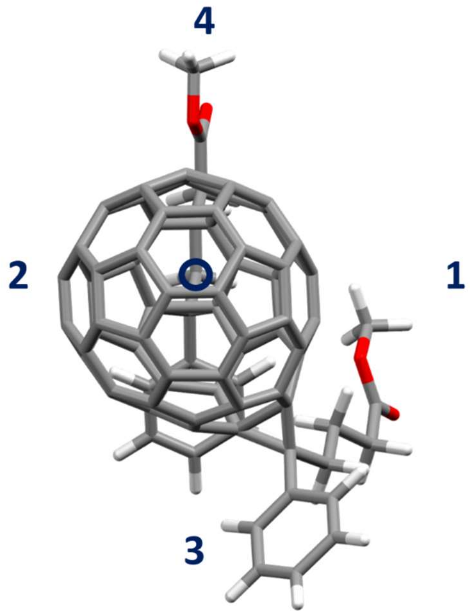

The assignment of priorities 1 and 2 is however tricky and virtually no examples con be found in literature to determine the stereochemistry of the quaternary carbon 61 or 62 part of the cyclopropyl bridge. Following a previous examples and guidelines for similar systems,[9,10] from the Schlegel diagram one can build a digraph to establish which of the two ligands (the one pointing towards the second addend on the cage or the other one) should have priority. Although drawing these digraphs becomes challenging for the *trans* isomers, an example digraph for the *cis-3* isomer (see below) shows that the priority is established when the main branch (1) reaches the cyclopropyl bridge of the second addend (carbon 62). The cyclopropyl group has priority over the double bonds of the C_{60} cage because of the absence of one duplicated atom (indicated as (C)) present instead in the main branch (9). Therefore, the highest priority should be assigned to the ligand pointing towards the second addend.

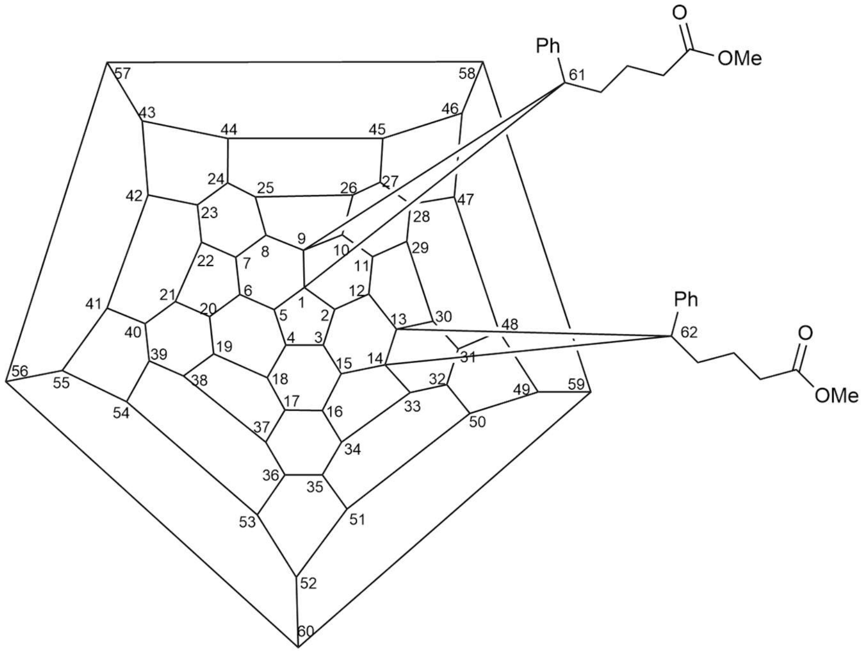

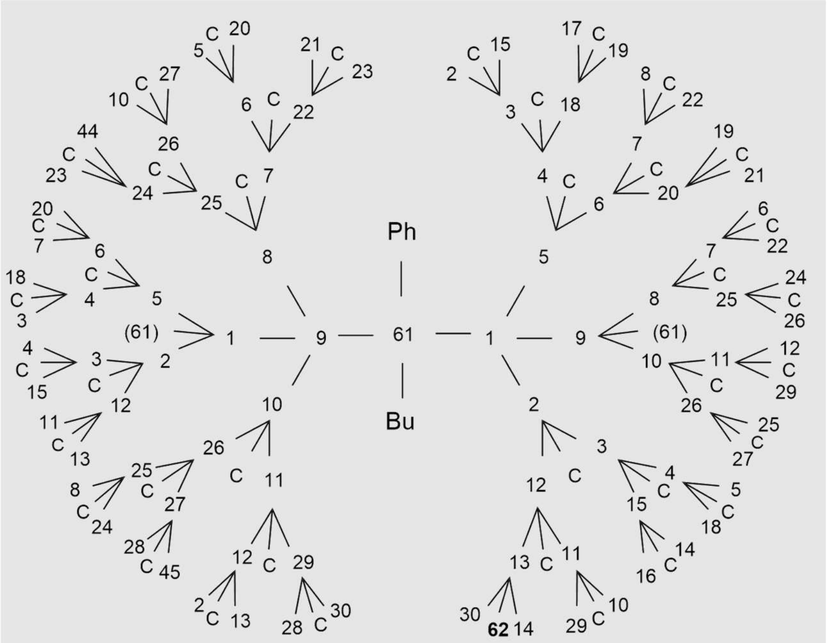

Finally, the two kinds of descriptors have to be combined to give, for the example above, the name (*S,R,* ^{f,s}*C*)13,14-bis[60]PCBM.

There are two exceptions: 1) the *equatorial* isomer has a different descriptor, namely *anti* or *syn*, since CIP priority for one of the two addends cannot be assigned. Therefore, *anti* is used when the phenyl group is pointing away from the second addend and *syn* when pointing towards it. 2) Substitution patterns *non-inherently chiral* (*cis-2* and *trans-4*) do not need the descriptor *C*/*A* where *S,S*/*R,R* is sufficient to describe their stereochemistry. Moreover, notice that any isomer having R,S configuration is equivalent to the S,R isomer by symmetry leaving the descriptor *C*/*A* unchanged.

Simulated UV-Vis and CD spectra

cis-2

(*S,S*)3,15-bis[60]PCBM

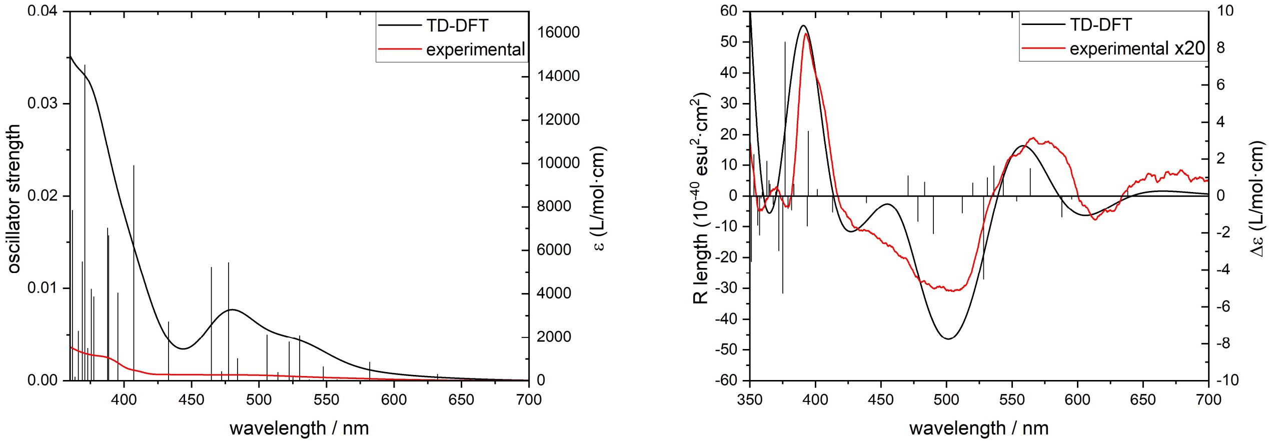

cis-3

(*R,S,* ^{f,s}*C*)13,14-bis[60]PCBM

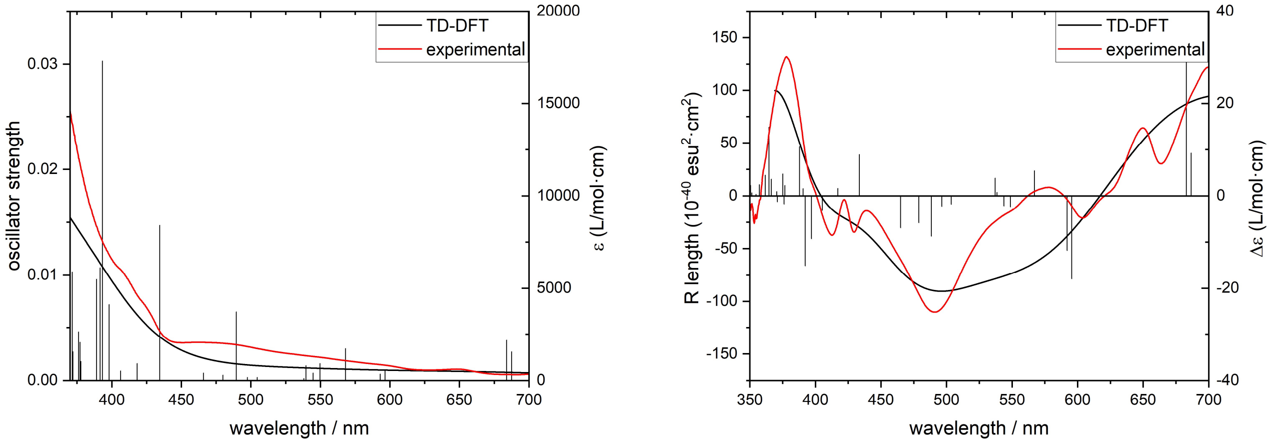

(*S*,*S*, ^{f,s}*C*)13,14-bis[60]PCBM

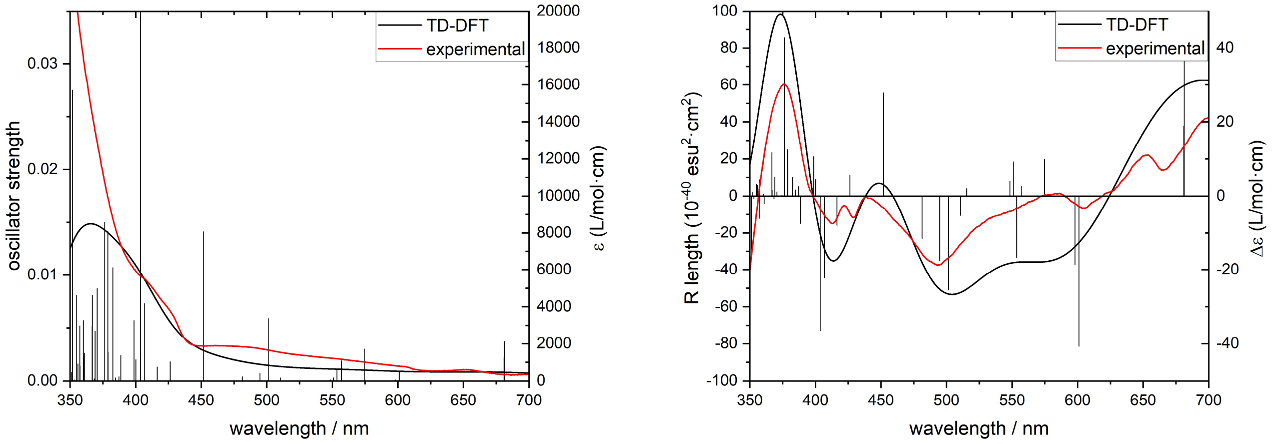

e

(*anti*,*S*)16,17-bis[60]PCBM

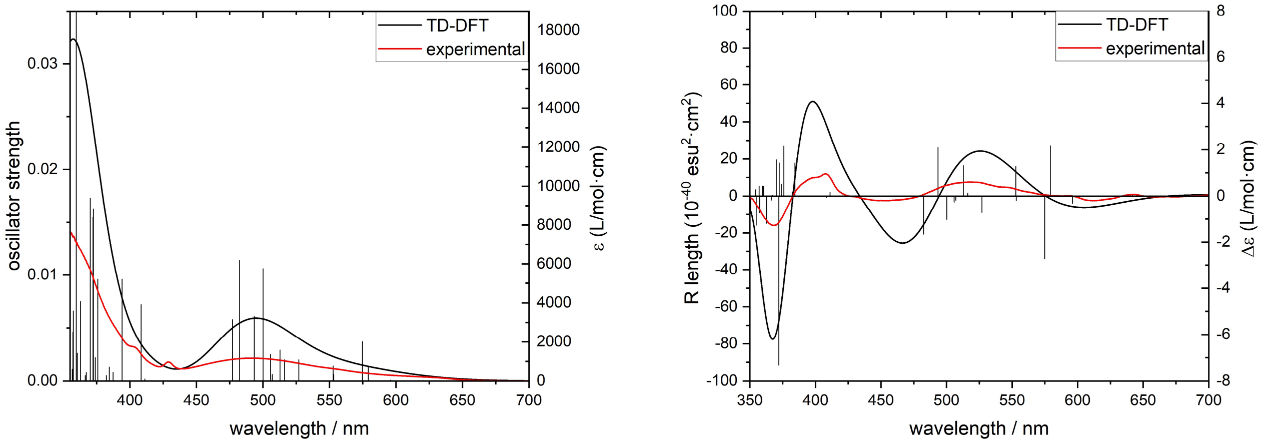

trans-4

(*S*,*S*)32,33-bis[60]PCBM

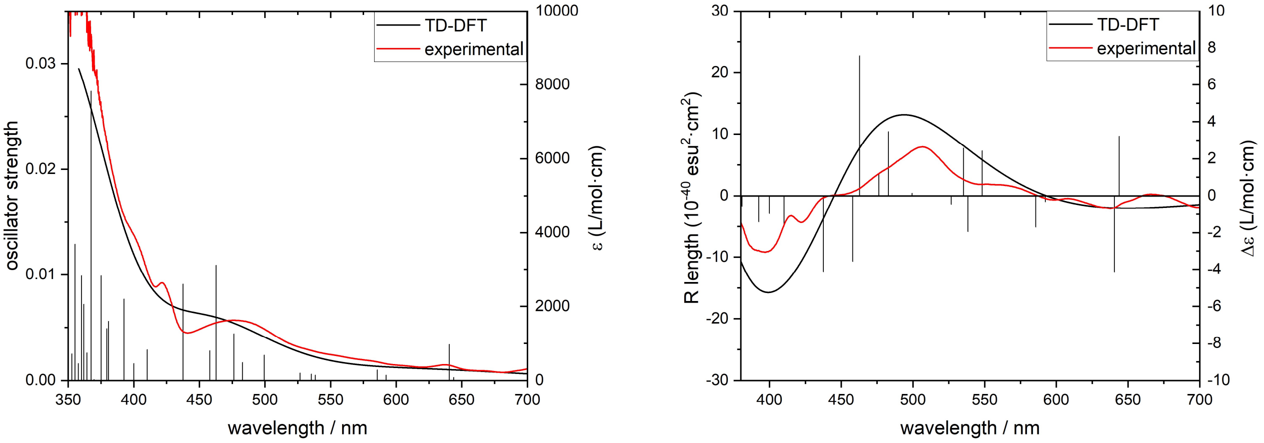

***trans*-3**

(*S*,*S*, *f,sC*)34,35-bis[60]PCBM

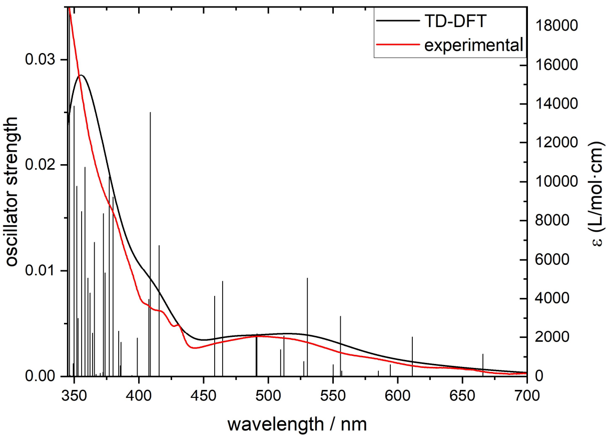

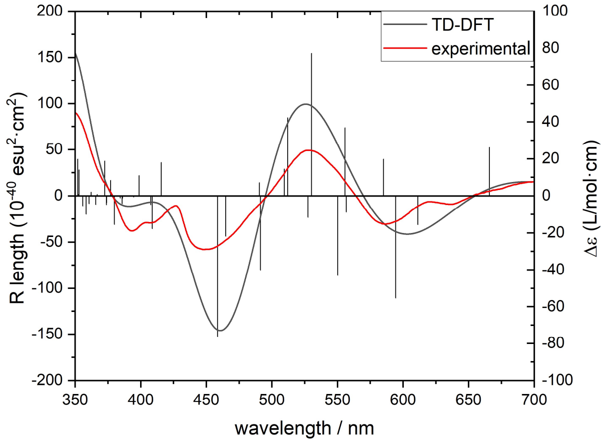

(*R*,*R*, *f,sC*)34,35-bis[60]PCBM

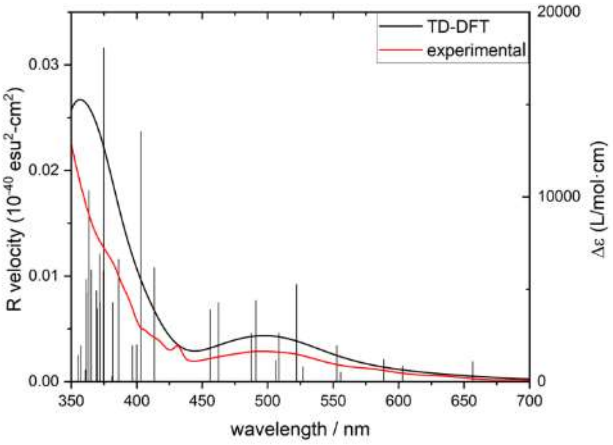

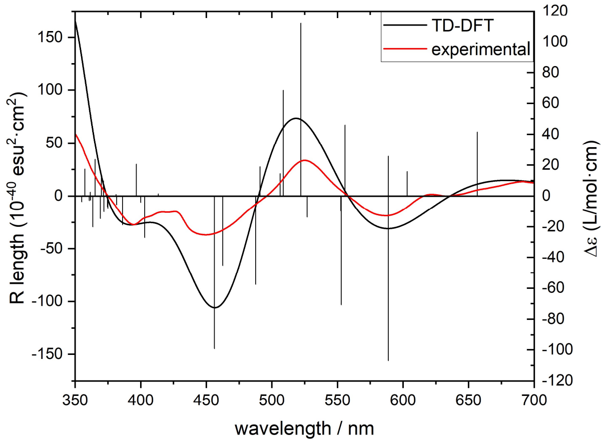

(*S*,*R*, *f,sC*)34,35-bis[60]PCBM

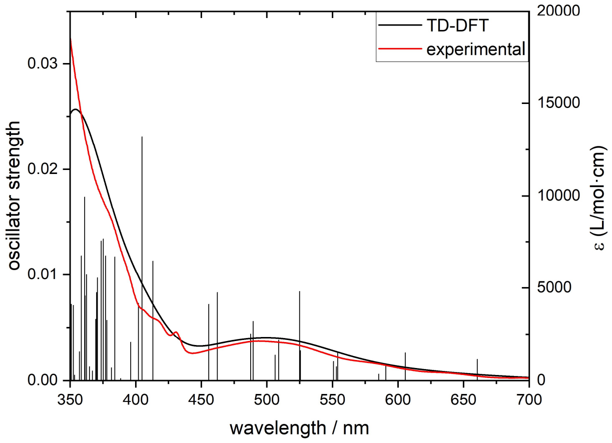

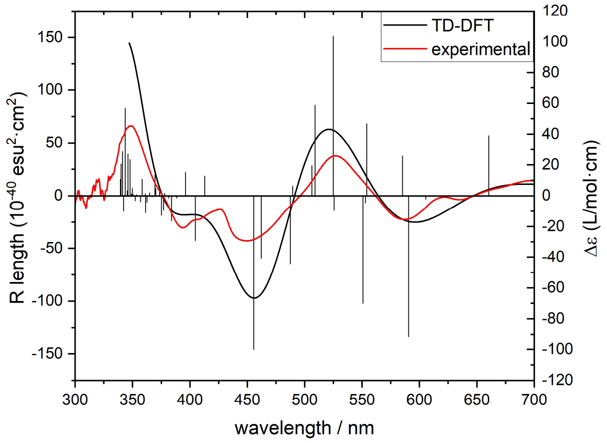

***trans*-2**

(*R*,*R*, ^{f,s}*A*)49,59-bis[60]PCBM

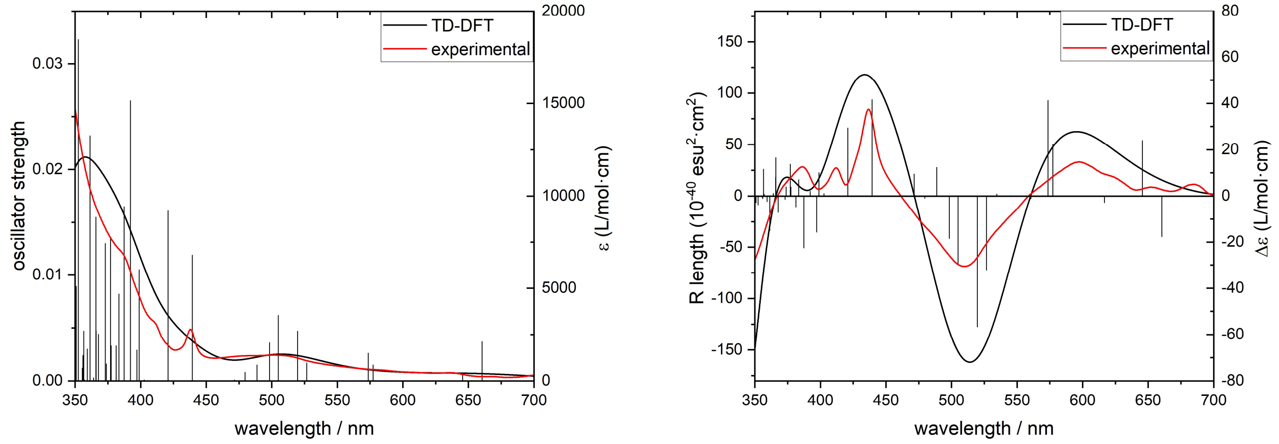

(*R*,*S*, ^{f,s}*A*)49,59-bis[60]PCBM

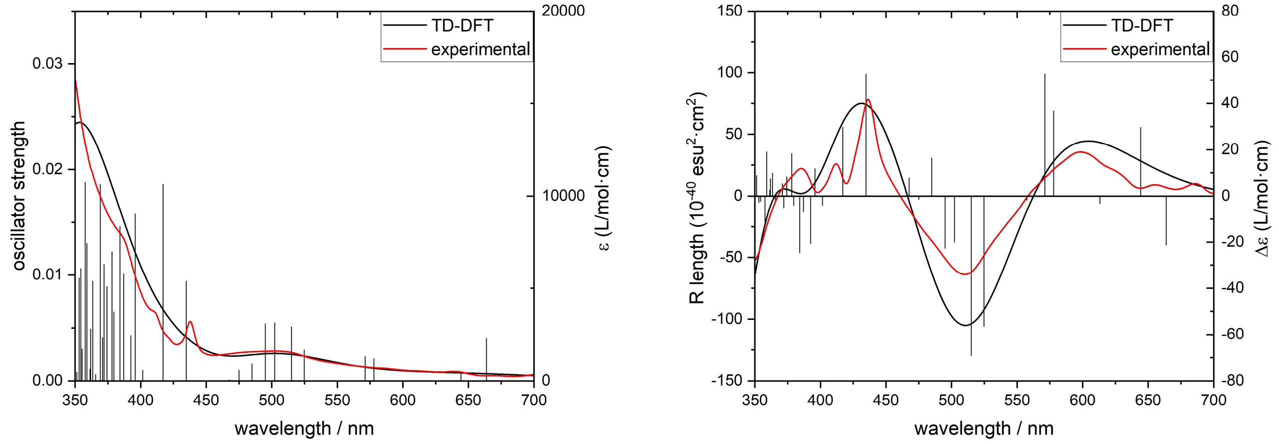

Figure S5 Simulated vs experimental UV-Vis and CD spectra.

Table S2 Values used by SpecDis to match simulated and experimental spectra: σ (the broadening factor) is the exponential half-width, i.e., half the bandwidth of the CD band at 1/*e* peak height; similarity factor and ΔESI (enantiomeric similarity index) reflect the discriminating power between the enantiomers.[11]

Isomer	σ / eV	shift / nm	similarity factor	ΔESI
(*S*,*S*)3,15	0.14	+6	0.8758	0.8672
(*S*,*R*, ^{f,s}*C*)13,14	0.3	-1	0.8797	0.8727
(*S*,*S*, ^{f,s}*C*)13,14	0.23	+10	0.8412	0.8277
(*anti*,*S*)16,17	0.2	+5	0.9452	0.9390
(*S*,*S*)32,33	0.3	-12	0.9528	0.9522
(*S*,*S*, ^{f,s}*C*)34,35	0.19	+10	0.8997	0.8945
(R,R, ^{f,s}*C*)34,35	0.2	+7	0.9573	0.9564
(S,*R*, ^{f,s}*C*)34,35	0.21	+7	0.9489	0.9487
(S,S, ^{f,s}*C*)49,59	0.2	+7	0.8643	0.8576
(R,*S*, ^{f,s}*A*)49,59	0.24	+2	0.9396	0.9391

Table S3 Experimental and calculated g_{abs} for all the 10 structural isomers at the main peaks maxima (wavelength indicated in brackets)

Structure	Bond Type	Experimental g_{abs} / 10^3 (nm)	Calculated g_{abs} / 10^3 (nm)
(*S,S*)3,15-C_1	*cis-2*	-1.4(611), +1.5(579), -1.1(511), +0.5(404)	-4.1(612), +3.0(565), -3.2(508), +1.0(394)
(*S,R,* ^{f,s}*C*)13,14-C_1	*cis-3*	+24.0(650), -6.6(605), -13.3(494), +2.8(381)	-26.4(524), +2.6(372)
(*S,S,* ^{f,s}*C*)13,14- C_2	*cis-3*	+18.1(653), -4.2(605), -10.7(495), +2.8(380)	-30.7(580), -34.0(512), +6.0(375)
(*anti,S*)16,17- C_1	*e*	-0.84(614), +0.64(525), -0.28(446), +0.72(410),-0.22(372)	-1.5(629), +1.0(529), -1.4(451), +1.6(410), -0.4(370)
(*S,S*)32,33- C_1	*trans-4*	-2.1(696), +2.22(511), -0.77(400)	-2.6(688), +4.2(524), -1.8(413)
(*S,S,* ^{f,s}*C*)34,35- C_2	*trans-3*	-18.4(594), +14.1(532), -19.7(445), -4.1(410)	-26.4(612), +23.3(528), -38.0(458), -0.9(395)
(R,R, ^{f,s}*C*)34,35- C_2	*trans-3*	-17.0(592), +13.0(528), -17.7(445), -3.9(403)	-28.7(597), +22.5(523), -40.1(452),
(S,*R,* ^{f,s}*C*)34,35- C_1	*trans-3*	-18.6(593), +14.2(529), -19.7(446), -4.2(409)	-22.8(606), +20.3(525), -35.3(454)
(S,S, ^{f,s}*C*)49,59- C_2	*trans-2*	+26.2(683), +30.1(600), -22.6(514), +16.0(434), +1.8(387)	+59.0(601), -52.0(519), +25.3(448), +0.8(375)
(R,*S,* ^{f,s}*A*)49,59- C_1	*trans-2*	+22.4(686), +33(602), -21.7(513), +15.6(433), +1.5(386)	+46.1(613), -38.8(515), +18.2(443), 0.27(373)

Excited States analysis

cis-2

(*S,S*)3,15-bis[60]PCBM

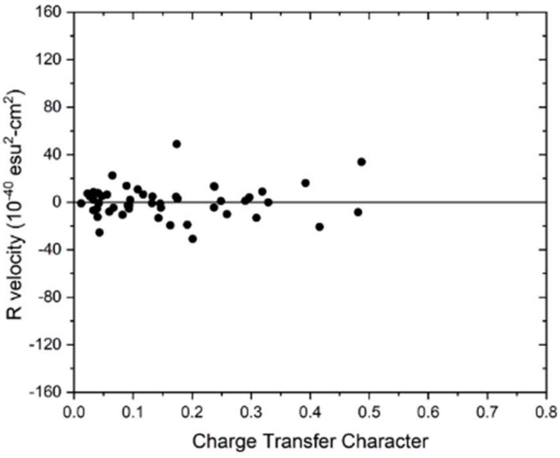

cis-3

(*S,R,* ^{f,s}*C*)13,14-bis[60]PCBM

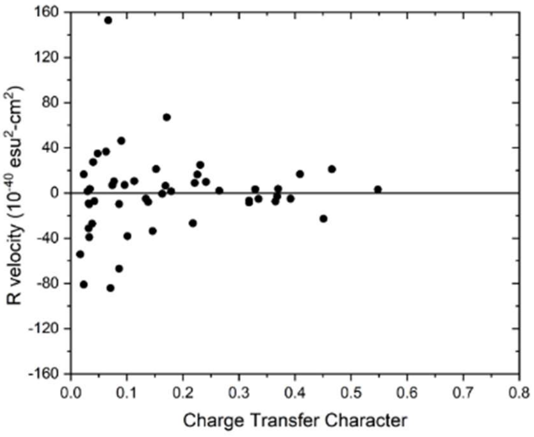

(*S,S,* ^{f,s}*C*)13,14-bis[60]PCBM

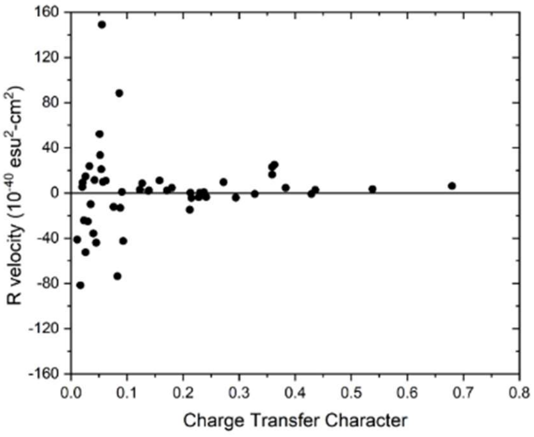

e

(*anti,S*)16,17-bis[60]PCBM

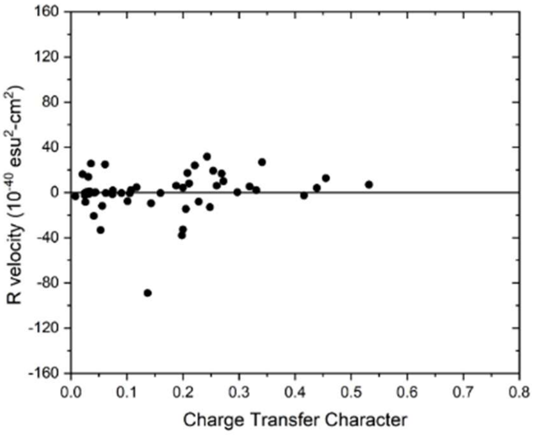

trans-4

(*S,S*)32,33-bis[60]PCBM

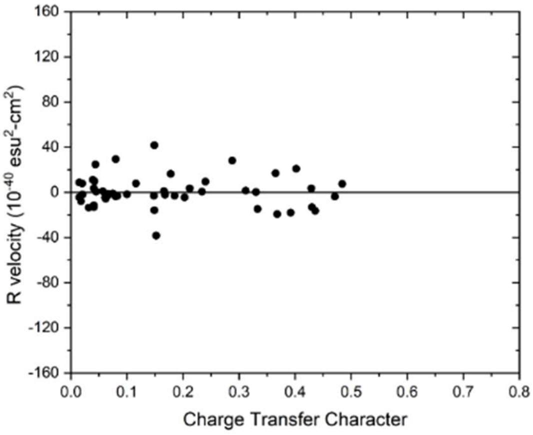

trans-3

(*S,S,* ^{f,s}*C*)34,35-bis[60]PCBM

(*S,S,* ^{f,s}*A*)34,35-bis[60]PCBM

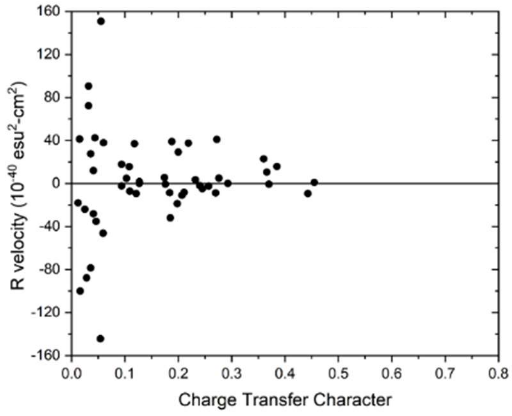

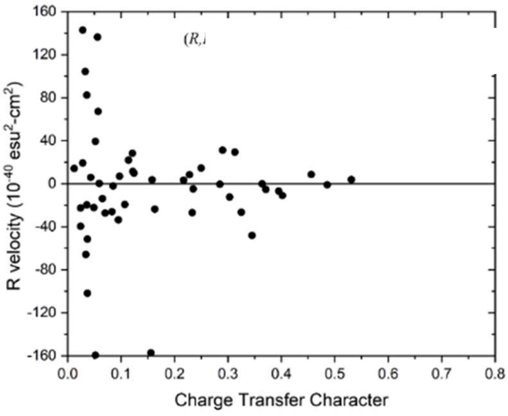

(*S,R,* ^{f,s}*C*)34,35-bis[60]PCBM

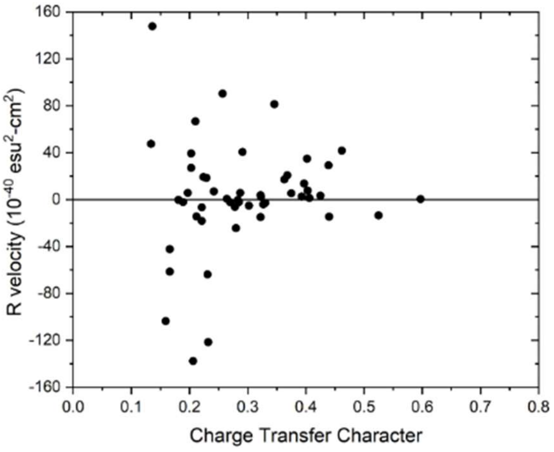

trans-2

(*S,S,* ^{f,s}*C*)49,59bis[60]PCBM

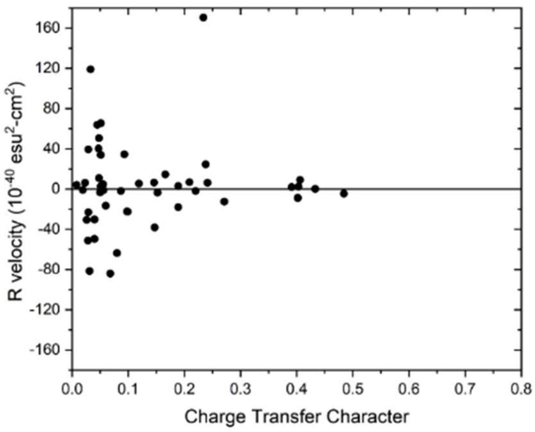

(*R,S,* ^{f,s}*A*)49,59-bis[60]PCBM

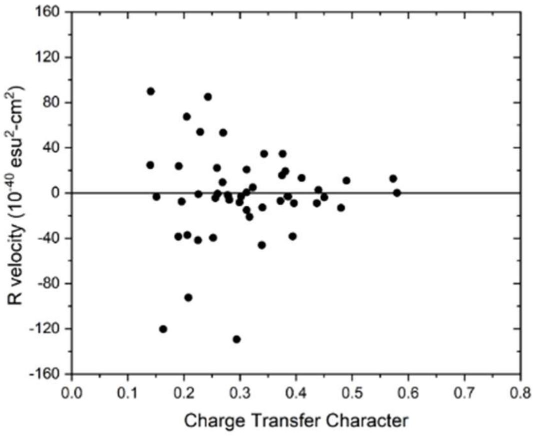

Figure S6 Relation between Rotatory strength (R) and Charge transfer character (CT).

TheoDORE output

Table S4. Output of TheoDORE excited state analysis: f = oscillator strength; Om = Omega matrix (Population analysis using the Loewdin localisation scheme); POS = average position of electron and holes; PR = arithmetic mean delocalization of electrons and holes; CT = Charge Transfer index; CTnt = net charge transfer distance; COH = Coherence Length; PRNTO = Population Ratio Natural Transition Orbitals; Z_eh = entanglement measurement (related to a measurement of information entropy); RMSeh = root means square e-h radius.[12]

cis-2
(*S,S*)3,15-bis[60]PCBM

state	dE(eV)	f	Om	POS	PR	CT	COH	CTnt	PRNTO	Z_HE	RMSeh	R (velocity)	E-M angle
1SingA	1.961	0.001	1	2.976	1.034	0.033	1.034	0.023	1.01	1.038	4.966	2.0544	88.41
2SingA	2.103	0	1	2.991	1.013	0.012	1.012	-0.011	1.215	1.418	4.925	-1.0305	102.36
3SingA	2.13	0.002	1.001	2.973	1.035	0.033	1.035	0.022	1.085	1.228	5.04	-6.9142	99.33
4SingA	2.222	0	1.001	2.97	1.042	0.041	1.042	0.003	1.248	1.554	5.016	7.6166	71.44
5SingA	2.264	0.002	1	2.969	1.044	0.042	1.044	0.017	1.509	1.901	5.071	-0.8851	91.22
6SingA	2.308	0	1	2.982	1.024	0.023	1.024	-0.009	2.114	2.539	4.949	7.2494	52.15
7SingA	2.339	0.005	1.001	2.972	1.034	0.033	1.034	0.006	1.619	1.872	5.039	8.5744	78.85
8SingA	2.361	0.001	1.001	2.977	1.031	0.03	1.031	0.003	2.07	2.422	5.047	6.3364	81.03
9SingA	2.373	0.004	1.001	2.968	1.045	0.043	1.044	0.035	2.267	2.584	5.021	-25.5558	97.07
10SingA	2.412	0.001	1.001	2.979	1.028	0.027	1.027	0.023	1.56	1.953	5.002	4.708	79.97
11SingA	2.45	0.005	1.002	2.97	1.041	0.039	1.041	0.021	2.36	2.7	5.119	-5.3019	169.76
12SingA	2.561	0.002	1.001	2.97	1.042	0.04	1.042	-0.001	1.12	1.307	5.008	-12.3647	96.86
13SingA	2.597	0.013	1.001	2.963	1.05	0.047	1.049	0.02	2.027	2.239	4.917	4.9404	87.81
14SingA	2.625	0.001	1.001	2.954	1.064	0.06	1.063	0.045	1.167	1.412	5.084	-7.8899	98.08
15SingA	2.668	0.012	1.002	2.957	1.059	0.056	1.059	0.033	2.042	2.269	5.087	6.315	87.59
16SingA	2.863	0.006	1	2.93	1.105	0.094	1.098	0.121	1.1	1.286	5.083	-2.2334	98.28
17SingA	3.045	0.023	1	2.928	1.104	0.093	1.099	0.094	1.51	2.023	4.809	-5.3245	94.15
18SingA	3.135	0.009	1	2.932	1.106	0.095	1.1	0.097	2.318	2.92	4.893	2.0714	87.57
19SingA	3.19	0.016	1	2.953	1.071	0.065	1.067	0.077	2.001	2.732	5.115	22.4356	68.87
20SingA	3.198	0.017	1	2.946	1.09	0.082	1.085	0.082	3.312	3.877	5.051	-10.622	96.95
21SingA	3.283	0.009	1	2.812	1.409	0.297	1.301	0.348	2.369	2.9	5.807	4.032	83.7
22SingA	3.299	0.01	1	2.957	1.073	0.067	1.069	0.059	2.826	3.228	4.862	-4.6137	98.02
23SingA	3.323	0.004	1	2.868	1.173	0.147	1.154	0.231	2.647	3.221	5.429	-4.5993	110.88
24SingA	3.342	0.034	1.001	2.841	1.211	0.174	1.181	0.287	3.479	4.419	5.372	48.8804	39.84
25SingA	3.359	0.013	1	2.838	1.256	0.201	1.217	0.277	3.476	4.138	5.474	-30.9636	118.41
26SingA	3.387	0.005	1	2.866	1.24	0.192	1.206	0.224	2.368	3.258	5.48	-18.9428	112.29
27SingA	3.409	0	1	2.871	1.218	0.175	1.187	0.222	3.991	4.503	5.344	2.9902	53.95
28SingA	3.427	0.018	1	2.932	1.102	0.091	1.095	0.105	3.164	3.712	5.193	-2.6122	94.34
29SingA	3.45	0.006	1	2.904	1.156	0.133	1.142	0.142	2.963	3.721	5.397	4.773	80.23
30SingA	3.456	0.009	1	2.91	1.134	0.117	1.125	0.142	3.578	4.321	5.373	6.5224	78.32
31SingA	3.473	0.005	1	2.917	1.121	0.108	1.117	0.09	4.294	5.011	5.118	10.7482	77.47
32SingA	3.507	0.002	1	2.782	1.33	0.249	1.261	0.396	3.303	4.027	5.806	1.0152	29.83
33SingA	3.516	0.016	1	2.88	1.171	0.146	1.163	0.151	3.784	4.436	5.412	-1.1375	91.81
34SingA	3.528	0.004	1	2.725	1.432	0.309	1.318	0.508	3.339	4.159	6.048	-13.2136	155.87
35SingA	3.542	0.008	1	2.79	1.352	0.259	1.292	0.333	3.423	4.257	5.733	-10.0434	136.05
36SingA	3.552	0.002	1	2.896	1.155	0.132	1.139	0.172	3.166	3.995	5.641	-0.8829	93.38
37SingA	3.571	0.017	1	2.852	1.304	0.237	1.252	0.25	3.225	4.378	5.751	13.3544	66.36
38SingA	3.591	0.005	1	2.665	1.667	0.416	1.431	0.612	3.28	4.116	6.458	-20.698	135.48
39SingA	3.604	0.018	1	2.732	1.586	0.481	1.391	0.494	3.009	4.077	6.667	-8.5159	111.13
40SingA	3.617	0.009	1	2.676	1.617	0.392	1.407	0.594	3.706	4.502	6.514	16.1399	44.78
41SingA	3.633	0.006	1	2.589	1.761	0.487	1.501	0.733	3.598	4.271	6.622	33.8425	41.71
42SingA	3.638	0.007	1	2.83	1.314	0.237	1.261	0.273	3.969	4.844	5.786	-4.4558	109.53
43SingA	3.658	0.002	1	2.851	1.206	0.173	1.191	0.224	4.035	4.861	5.578	4.5399	77.92
44SingA	3.663	0.005	1	2.797	1.443	0.329	1.35	0.321	3.178	4.07	6.232	-0.1191	90
45SingA	3.68	0.011	1	2.858	1.196	0.163	1.178	0.215	2.986	4.069	5.549	-19.5536	137.6
46SingA	3.685	0.015	1	2.781	1.447	0.319	1.359	0.342	3.937	4.537	6.161	8.8532	73.49

47SingA	3.692	0.003	1	2.883	1.163	0.143	1.16	0.14	4.512	5.126	5.611	-13.3497	113.36
48SingA	3.71	0.005	1	2.753	1.394	0.29	1.315	0.43	3.943	4.689	6.15	1.1631	84.1
49SingA	3.725	0.01	1	2.923	1.097	0.089	1.096	0.085	3.738	4.819	5.398	13.8166	31.05
50SingA	3.741	0.005	1	2.819	1.317	0.238	1.263	0.292	4.78	5.506	5.746	13.0623	29.68

cis-3

(*S,R*, $^{f,s}\underline{C}$)13,14-bis[60]PCBM

state	dE(eV)	f	Om	POS	PR	CT	COH	CTnt	PRNTO	Z_HE	RMSeh	R (velocity)	E-M angle
1SingA	1.803	0.003	1.001	2.951	1.068	0.063	1.065	0.082	1.019	1.067	5.036	36.6713	74.77
2SingA	1.813	0.004	1.001	2.949	1.072	0.067	1.07	0.07	1.028	1.088	5.024	152.8487	1.75
3SingA	2.079	0.001	1	2.983	1.024	0.023	1.024	0.007	1.04	1.128	4.93	-80.9585	158.56
4SingA	2.091	0.001	1.001	2.988	1.017	0.017	1.017	0.004	1.065	1.188	4.909	-54.2145	140.37
5SingA	2.182	0.003	1	2.97	1.042	0.04	1.041	0.025	2.249	2.528	5.048	27.4513	59.37
6SingA	2.256	0.002	1.002	2.975	1.034	0.033	1.034	0.019	1.421	1.788	5.053	-9.8979	135.59
7SingA	2.276	0.001	1.002	2.967	1.044	0.042	1.043	0.03	2.092	2.294	5.158	-7.1659	141.63
8SingA	2.298	0.001	1.001	2.977	1.031	0.03	1.031	0.013	1.292	1.631	4.947	1.4334	89.03
9SingA	2.304	0	1.001	2.983	1.023	0.023	1.023	0.016	1.213	1.494	4.948	16.4937	2.75
10SingA	2.457	0	1.001	2.975	1.035	0.034	1.035	-0.005	1.288	1.576	5.007	3.5909	82.73
11SingA	2.492	0	1.001	2.976	1.033	0.032	1.033	0.027	1.585	1.934	4.953	-9.1815	147.51
12SingA	2.533	0.006	1.001	2.975	1.034	0.033	1.034	0.001	1.386	1.692	4.955	-39.0025	99.77
13SingA	2.584	0.001	1.002	2.971	1.04	0.038	1.04	0.025	1.461	1.802	5.021	-27.1087	170.91
14SingA	2.661	0.001	1.001	2.976	1.033	0.032	1.033	-0.004	1.158	1.362	4.955	-31.2217	176.49
15SingA	2.854	0.015	1.001	2.963	1.051	0.048	1.05	0.015	1.089	1.273	4.827	34.981	81.94
16SingA	2.965	0.002	1	2.942	1.081	0.074	1.076	0.099	2.167	2.475	4.436	6.8912	31.1
17SingA	3.052	0.001	1	2.933	1.094	0.086	1.09	0.103	1.798	2.314	4.908	-9.6399	138.6
18SingA	3.114	0.007	1	2.919	1.113	0.101	1.105	0.14	2.103	2.394	5.54	-38.1072	113.4
19SingA	3.153	0.03	1	2.933	1.096	0.086	1.09	0.102	2.855	3.273	4.91	-66.8403	165.53
20SingA	3.165	0.011	1	2.931	1.108	0.096	1.1	0.116	2.319	2.895	5.119	7.1853	63.17
21SingA	3.187	0.01	1	2.934	1.1	0.09	1.095	0.083	3.244	4.102	4.636	46.2377	6.57
22SingA	3.282	0.002	1	2.866	1.28	0.221	1.226	0.242	3.064	3.495	5.398	8.9066	46.65
23SingA	3.287	0.004	1	2.817	1.421	0.318	1.307	0.338	1.945	2.325	5.76	-8.5088	117.8
24SingA	3.296	0.005	1	2.567	1.629	0.466	1.398	0.842	1.69	2.331	6.21	21.0354	26.94
25SingA	3.333	0.003	1	2.758	1.557	0.392	1.386	0.434	2.395	3.276	6.057	-5.0178	126.99
26SingA	3.337	0.01	1	2.712	1.571	0.37	1.385	0.534	2.566	3.296	6.001	3.6959	78.41
27SingA	3.375	0.017	1	2.676	1.647	0.409	1.433	0.588	2.888	3.742	6.193	16.7471	62.36
28SingA	3.391	0.012	1	2.866	1.207	0.171	1.189	0.204	4.259	4.838	5.319	66.9252	45.27
29SingA	3.418	0.002	1	2.882	1.182	0.152	1.163	0.198	3.278	3.957	5.077	21.245	25.81
30SingA	3.447	0.003	1	2.88	1.198	0.163	1.174	0.204	4.162	4.882	5.216	-0.7605	94.89
31SingA	3.461	0.009	1	2.933	1.084	0.077	1.081	0.103	3.534	4.461	4.996	10.2839	68.32
32SingA	3.485	0.007	1	2.848	1.22	0.179	1.19	0.267	4.374	5.082	5.382	1.4536	84.16
33SingA	3.517	0.001	1	2.799	1.454	0.329	1.326	0.372	1.621	2.346	6.128	3.2908	64.03
34SingA	3.527	0.002	1	2.798	1.321	0.241	1.254	0.373	2.371	3.305	5.688	9.8822	25.35
35SingA	3.536	0.005	1	2.826	1.306	0.231	1.246	0.314	2.145	3.029	5.96	24.8444	32.65
36SingA	3.546	0.003	1	2.862	1.209	0.169	1.179	0.246	3.012	3.846	5.704	6.342	62.09
37SingA	3.566	0.01	1	2.915	1.13	0.113	1.119	0.14	2.647	3.546	5.531	10.59	69.77
38SingA	3.576	0.001	1	2.897	1.161	0.138	1.148	0.154	3.281	4.306	5.588	-7.9996	129.78
39SingA	3.591	0.006	1	2.822	1.359	0.265	1.28	0.317	2.908	3.699	5.848	2.0038	69.03
40SingA	3.61	0.005	1	2.909	1.155	0.134	1.142	0.143	3.058	4.174	5.42	-4.968	101.21
41SingA	3.628	0.018	1	2.901	1.172	0.146	1.158	0.138	4.593	5.362	5.417	-33.6311	114.28
42SingA	3.639	0.005	1	2.948	1.076	0.071	1.076	-0.02	1.796	2.327	5.425	-84.155	166.84
43SingA	3.647	0.014	1	2.811	1.297	0.226	1.239	0.345	2.685	3.544	6.055	16.2527	56.78
44SingA	3.664	0.003	1	2.778	1.508	0.365	1.365	0.383	1.861	2.657	6.118	-7.3739	128.38
45SingA	3.679	0.012	1	2.757	1.468	0.318	1.347	0.426	4.129	5.004	6.034	-6.5807	104.99
46SingA	3.701	0.013	1	2.723	1.499	0.335	1.358	0.495	3.27	4.383	6.179	-5.1725	105.53
47SingA	3.725	0.011	1	2.632	1.723	0.451	1.464	0.67	3.915	4.823	6.243	-22.7509	161.39
48SingA	3.728	0.03	1	2.751	1.548	0.368	1.386	0.439	3.406	4.653	5.98	-2.9876	93.22
49SingA	3.741	0.005	1	2.66	1.757	0.548	1.452	0.647	1.65	2.462	6.608	3.18	11.15
50SingA	3.748	0.017	1	2.822	1.283	0.218	1.236	0.309	3.964	5.102	5.625	-26.7564	149.54

cis-3

($S,S,$ $^{f,s}C$)13,14-bis[60]PCBM

state	dE(eV)	f	Om	POS	PR	CT	COH	CTnt	PRNTO	Z_HE	RMSeh	R (velocity)	E-M angle
1SingA	1.847	0.004	1.001	2.958	1.058	0.055	1.057	0.054	1.03	1.095	5.03	149.0162	1.25
2SingA	1.848	0.002	1.001	2.961	1.055	0.052	1.053	0.065	1.02	1.072	5.052	33.4627	74.44
3SingA	2.098	0.001	1.001	2.987	1.018	0.017	1.018	-0.003	1.215	1.474	4.918	-81.6581	163.89
4SingA	2.109	0	1.001	2.991	1.011	0.011	1.011	0.004	1.293	1.514	4.944	-41.262	90
5SingA	2.195	0.003	1	2.974	1.035	0.033	1.034	0.018	2.463	2.728	5.06	23.6934	61.2
6SingA	2.267	0.002	1.002	2.985	1.02	0.02	1.02	0.003	1.377	1.718	5.018	5.2039	90
7SingA	2.281	0.001	1.001	2.982	1.024	0.023	1.024	0.01	1.959	2.27	5.006	-24.2276	109.82
8SingA	2.292	0	1.002	2.968	1.044	0.042	1.043	0.028	2.69	2.952	5.167	11.4085	2.05
9SingA	2.302	0	1.001	2.985	1.021	0.021	1.021	0.015	1.317	1.669	4.976	9.207	90
10SingA	2.453	0	1.001	2.98	1.027	0.026	1.027	-0.008	1.338	1.678	5.011	14.7496	90
11SingA	2.477	0	1.001	2.974	1.036	0.035	1.036	0.034	1.556	1.92	4.997	-9.8203	173.07
12SingA	2.522	0.006	1.001	2.98	1.027	0.026	1.027	-0.004	1.34	1.641	4.964	-52.4772	103.71
13SingA	2.557	0.001	1.002	2.969	1.042	0.04	1.042	0.03	1.547	1.904	5.071	-35.8713	90
14SingA	2.629	0	1.001	2.977	1.031	0.03	1.031	0	1.159	1.368	5.001	-25.1803	90
15SingA	2.805	0.014	1.001	2.961	1.054	0.051	1.054	0.026	1.074	1.234	4.906	52.2272	78.12
16SingA	2.977	0.002	1	2.953	1.067	0.062	1.064	0.08	2.168	2.463	4.435	10.8623	21.5
17SingA	3.05	0.001	1	2.943	1.083	0.076	1.079	0.087	1.83	2.331	4.917	-12.297	90
18SingA	3.124	0.007	1	2.93	1.104	0.093	1.097	0.121	2.177	2.512	5.493	-42.4742	117.48
19SingA	3.149	0.038	1.001	2.937	1.092	0.083	1.087	0.1	2.395	2.98	4.989	-73.6033	90
20SingA	3.177	0.002	1	2.957	1.061	0.057	1.059	0.071	1.989	2.666	4.834	9.7747	90
21SingA	3.189	0.006	1	2.959	1.058	0.054	1.056	0.045	3.638	4.423	4.509	21.1099	11.59
22SingA	3.274	0.002	1	2.84	1.277	0.212	1.222	0.302	2.819	3.236	5.541	-14.8342	90
23SingA	3.286	0	1	2.71	1.601	0.383	1.385	0.562	1.943	2.492	6.135	4.4903	90
24SingA	3.309	0	1	2.591	1.903	0.538	1.492	0.79	1.936	2.507	6.563	3.5229	90
25SingA	3.328	0.011	1	2.793	1.379	0.272	1.291	0.376	3.165	3.836	5.735	9.5523	68.57
26SingA	3.36	0.003	1	2.726	1.544	0.359	1.386	0.493	3.631	4.277	6.16	16.2933	7.15
27SingA	3.361	0.014	1	2.724	1.561	0.363	1.375	0.52	1.645	2.326	6.052	24.9347	90
28SingA	3.384	0.015	1.001	2.935	1.094	0.086	1.092	0.072	3.898	4.607	5.113	88.4227	37.37
29SingA	3.4	0	1	2.895	1.164	0.139	1.147	0.179	3.288	3.887	5.135	2.14	90
30SingA	3.437	0.009	1	2.895	1.163	0.138	1.147	0.173	4.14	4.82	5.425	1.7831	84.16
31SingA	3.453	0.005	1	2.88	1.191	0.158	1.167	0.213	4.148	4.737	5.347	11.0152	61.62
32SingA	3.458	0	1	2.676	1.696	0.429	1.424	0.626	2.347	3.115	6.17	-0.9495	90
33SingA	3.473	0.008	1	2.67	1.711	0.436	1.431	0.634	4.149	4.557	6.471	2.8106	10.15
34SingA	3.475	0.005	1	2.726	1.554	0.359	1.371	0.517	2.481	3.5	6.339	23.0762	19.29
35SingA	3.505	0	1	2.838	1.278	0.213	1.225	0.3	2.016	2.856	5.689	0.1485	90
36SingA	3.532	0.003	1	2.826	1.302	0.228	1.243	0.313	3.096	4.074	5.989	-3.766	112.84
37SingA	3.539	0.002	1	2.819	1.319	0.237	1.248	0.337	2.746	3.601	6	0.7123	86.76
38SingA	3.54	0.006	1	2.932	1.101	0.091	1.095	0.106	2.931	3.718	5.631	0.9548	15.04
39SingA	3.567	0.001	1	2.933	1.097	0.088	1.092	0.102	3.087	3.949	5.471	-13.2343	179.09
40SingA	3.569	0.005	1	2.904	1.148	0.127	1.133	0.168	2.596	3.384	5.669	8.5301	59.6
41SingA	3.582	0.002	1	2.862	1.225	0.18	1.193	0.239	3.591	4.235	5.689	4.6513	90
42SingA	3.593	0.008	1	2.906	1.141	0.123	1.134	0.129	4.559	5.204	5.288	2.8911	87.31
43SingA	3.614	0	1	2.746	1.494	0.328	1.348	0.459	2.208	2.909	6.118	-0.8103	90
44SingA	3.626	0.004	1	2.868	1.212	0.171	1.181	0.24	2.751	3.659	5.956	2.3006	17.85
45SingA	3.627	0.028	1.001	2.827	1.307	0.23	1.244	0.316	3.483	4.527	5.802	0.1749	90
46SingA	3.633	0.001	1	2.966	1.047	0.045	1.047	-0.036	1.639	2.038	5.454	-44.0207	90
47SingA	3.679	0.002	1	2.838	1.278	0.215	1.229	0.293	3.455	4.429	5.902	-4.241	117.53
48SingA	3.712	0.003	1	2.772	1.417	0.294	1.332	0.366	3.199	4.261	6.293	-4.1676	101.48
49SingA	3.72	0.001	1	2.815	1.328	0.241	1.255	0.335	2.167	2.556	6.158	-3.6624	90
50SingA	3.723	0.018	1	2.484	2.012	0.68	1.527	0.994	1.89	2.833	7.09	6.1592	55.55

e

(*anti,S*)16,17-bis[60]PCBM

state	dE(eV)	f	Om	POS	PR	CT	COH	CTnt	PRNTO	Z_HE	RMSeh	R (velocity)	E-M angle
1SingA	1.947	0	1.001	2.973	1.029	0.029	1.029	0.041	1.033	1.107	4.984	0.5888	88.33
2SingA	2.098	0	1	2.995	1.009	0.008	1.009	-0.002	1.034	1.106	4.924	-3.4403	95.69
3SingA	2.159	0.001	1	2.971	1.038	0.036	1.037	0.023	1.575	1.789	4.972	25.5052	73.98
4SingA	2.176	0.004	1	2.962	1.056	0.053	1.054	0.062	1.185	1.414	5.015	-33.1851	106.26
5SingA	2.261	0.001	1.001	2.978	1.026	0.025	1.026	0.013	1.93	2.063	5.039	-0.4097	90.9
6SingA	2.263	0.001	1.001	2.972	1.033	0.031	1.032	0.014	2.051	2.286	5.055	13.8163	59.03
7SingA	2.375	0.002	1	2.985	1.026	0.026	1.026	-0.012	1.075	1.211	4.937	-8.5204	94.2
8SingA	2.425	0.002	1.002	2.977	1.034	0.033	1.034	0.02	1.926	2.294	5.109	1.0718	87.23
9SingA	2.441	0.003	1	2.981	1.021	0.021	1.021	-0.021	1.116	1.304	4.938	16.1068	83.8
10SingA	2.469	0.001	1.001	2.983	1.025	0.025	1.025	-0.012	1.292	1.564	4.999	-2.5196	101.39
11SingA	2.476	0.003	1.001	2.976	1.033	0.032	1.033	-0.008	1.391	1.66	5.006	-1.41	91.53
12SingA	2.504	0.011	1.001	2.956	1.059	0.056	1.059	0.029	1.266	1.582	5.007	-11.8229	93.24
13SingA	2.538	0.006	1.002	2.957	1.065	0.061	1.064	0.046	1.479	1.925	5.21	24.9256	49.57
14SingA	2.597	0.011	1.001	2.976	1.043	0.041	1.043	0.002	1.362	1.669	4.969	-20.7385	95.52
15SingA	2.626	0.006	1.001	2.97	1.044	0.042	1.043	-0.001	1.245	1.508	5.044	-0.1403	90
16SingA	3.051	0	1	2.929	1.08	0.075	1.076	0.129	1.117	1.319	4.898	1.9337	54.78
17SingA	3.073	0.007	1	2.917	1.12	0.105	1.11	0.146	1.471	2.053	4.998	-0.6088	90.54
18SingA	3.187	0.01	1	2.961	1.046	0.044	1.045	0.06	1.53	2.1	4.912	0.2215	88.47
19SingA	3.242	0.001	1	2.914	1.099	0.09	1.092	0.157	2.312	2.746	4.546	-0.5431	97
20SingA	3.267	0.001	1	2.878	1.256	0.208	1.217	0.21	3.208	4.041	5.271	17.1746	17.58
21SingA	3.285	0.001	1	2.93	1.121	0.107	1.112	0.107	3.415	4.013	4.737	1.9932	27.78
22SingA	3.343	0.01	1	2.733	1.509	0.341	1.36	0.5	2.731	3.289	5.897	26.9083	54.41
23SingA	3.36	0.002	1	2.78	1.351	0.26	1.272	0.411	3.302	3.754	5.802	6.0179	65.5
24SingA	3.373	0.016	1	2.825	1.363	0.269	1.28	0.318	3.7	4.411	5.78	16.7191	59.83
25SingA	3.377	0.015	1	2.909	1.16	0.137	1.146	0.141	3.274	4.042	5.298	-89.0461	148
26SingA	3.395	0.017	1	2.767	1.329	0.254	1.258	0.438	3.344	3.944	6.03	19.2665	24.36
27SingA	3.423	0.001	1	2.939	1.08	0.074	1.078	0.081	3.32	3.855	5.387	-1.4979	99.34
28SingA	3.431	0.001	1	2.944	1.065	0.062	1.065	0.064	3.523	4.134	5.363	-0.5122	106.52
29SingA	3.465	0.007	1	2.872	1.257	0.205	1.216	0.225	3.255	4.089	5.892	-14.7041	105.04
30SingA	3.488	0.003	1	2.709	1.43	0.319	1.321	0.543	2.049	3.044	5.938	5.2754	68.85
31SingA	3.496	0.054	1.001	2.912	1.137	0.117	1.126	0.126	4.431	5.229	5.386	4.5733	88.05
32SingA	3.517	0.007	1	2.887	1.169	0.143	1.154	0.178	3.557	4.521	5.246	-9.6033	108.94
33SingA	3.521	0.005	1	2.888	1.229	0.188	1.201	0.169	4.196	5.065	5.565	6.0028	67.78
34SingA	3.525	0.001	1	2.9	1.191	0.16	1.17	0.152	3.707	4.707	5.44	-0.489	94.14
35SingA	3.544	0.014	1	2.816	1.336	0.248	1.265	0.325	4.451	5.127	5.703	-13.0719	103.13
36SingA	3.548	0.039	1	2.861	1.255	0.2	1.216	0.223	3.724	4.719	5.546	4.1311	87.53
37SingA	3.587	0.005	1	2.842	1.296	0.228	1.246	0.256	3.972	4.876	5.794	-8.174	128.42
38SingA	3.599	0.008	1	2.842	1.286	0.221	1.24	0.268	3.459	4.394	5.758	23.8657	38.79
39SingA	3.612	0.022	1	2.586	1.591	0.439	1.402	0.769	2.849	3.889	6.296	4.0024	85.58
40SingA	3.615	0.003	1	2.578	1.63	0.455	1.419	0.784	3.901	4.763	6.341	12.6449	43.33
41SingA	3.638	0.004	1	2.737	1.485	0.331	1.361	0.451	3.549	4.284	6.21	2.0802	67.68
42SingA	3.641	0.002	1	2.696	1.658	0.416	1.439	0.544	3.7	4.483	6.312	-2.7032	123.86
43SingA	3.649	0.004	1	2.633	1.845	0.532	1.491	0.679	3.107	3.998	6.768	6.8905	29.87
44SingA	3.668	0.004	1	2.805	1.374	0.272	1.295	0.346	2.999	3.829	5.854	9.9122	29.52
45SingA	3.684	0.004	1	2.801	1.32	0.243	1.272	0.319	3.481	4.894	5.767	31.7006	32.03
46SingA	3.703	0.003	1	2.832	1.253	0.2	1.219	0.276	4.137	4.838	6.023	-32.736	153.97
47SingA	3.708	0.01	1	2.871	1.248	0.198	1.217	0.184	3.35	4.352	5.564	-37.9992	139.69
48SingA	3.721	0.002	1	2.914	1.113	0.101	1.109	0.115	1.991	2.872	5.398	-7.6562	134.09
49SingA	3.733	0.007	1	2.851	1.27	0.211	1.234	0.231	3.045	3.954	5.938	8.0233	69.94
50SingA	3.737	0.001	1	2.741	1.414	0.297	1.317	0.464	3.715	4.729	6.239	0.1579	90

trans-4

(*S,S*)32,33-bis[60]PCBM

state	dE(eV)	f	Om	POS	PR	CT	COH	CTnt	PRNTO	Z_HE	RMSeh	R (velocity)	E-M angle
1SingA	1.891	0	1	2.969	1.044	0.042	1.043	0.048	1.029	1.094	4.999	9.8278	76.95
2SingA	1.9	0.003	1.001	2.969	1.043	0.041	1.042	0.04	1.042	1.128	5.042	-13.1936	95.15
3SingA	2.052	0.001	1	2.985	1.021	0.021	1.021	0.007	1.119	1.283	4.95	-1.9551	92.02
4SingA	2.075	0.001	1.001	2.988	1.016	0.015	1.016	0.011	1.219	1.42	4.956	-4.436	92.55
5SingA	2.213	0	1	2.985	1.02	0.02	1.02	0.006	2.127	2.439	4.952	7.9123	33.33
6SingA	2.253	0.001	1.001	2.987	1.018	0.018	1.018	0.007	1.491	1.762	4.996	-7.9186	101.26
7SingA	2.265	0.001	1.002	2.989	1.015	0.015	1.015	0.002	1.259	1.532	5.008	8.7891	71.89
8SingA	2.302	0.001	1.001	2.985	1.021	0.02	1.021	0.015	1.238	1.537	4.971	-2.0087	94.72
9SingA	2.424	0.002	1.001	2.956	1.061	0.057	1.06	0.018	1.447	1.812	5.165	0.942	87.52
10SingA	2.505	0.002	1.001	2.97	1.041	0.039	1.04	0.043	1.331	1.676	4.989	11.1421	80.32
11SingA	2.539	0.004	1.002	2.968	1.043	0.041	1.043	0.022	2.086	2.449	5.067	3.477	72.26
12SingA	2.611	0.011	1.002	2.966	1.046	0.044	1.046	0	1.786	2.036	5.11	24.6544	82.8
13SingA	2.638	0.003	1	2.976	1.034	0.032	1.033	-0.031	1.063	1.191	4.935	-13.602	95.75
14SingA	2.758	0.009	1.001	2.969	1.042	0.041	1.042	-0.02	1.079	1.238	5.013	-11.6217	94.38
15SingA	2.937	0.003	1	2.953	1.065	0.061	1.065	0.016	1.06	1.198	4.933	-4.6069	92.61
16SingA	3.01	0.002	1	2.952	1.071	0.066	1.068	0.082	2.276	2.58	4.417	-2.7529	108.5
17SingA	3.065	0.008	1	2.94	1.092	0.083	1.087	0.093	1.481	2.059	4.905	-3.2934	93.33
18SingA	3.157	0.006	1	2.921	1.113	0.1	1.104	0.14	1.871	2.454	5.123	-1.8284	93.11
19SingA	3.168	0.005	1	2.95	1.082	0.075	1.077	0.079	2.13	2.784	4.984	-1.0274	91.59
20SingA	3.203	0.01	1	2.949	1.072	0.067	1.069	0.076	3.094	3.646	5.095	-1.4157	91.13
21SingA	3.248	0	1	2.968	1.048	0.045	1.047	0.046	2.057	2.549	4.938	0.6341	54.53
22SingA	3.267	0.027	1	2.911	1.171	0.148	1.157	0.127	2.263	2.974	5.296	-3.1038	93.53
23SingA	3.294	0.003	1	2.587	1.571	0.436	1.376	0.804	1.589	2.144	6.2	-16.4172	154.28
24SingA	3.315	0.007	1	2.801	1.465	0.365	1.331	0.369	2.029	2.775	6.037	16.9483	66.28
25SingA	3.332	0.01	1	2.75	1.554	0.368	1.371	0.472	2.044	2.975	6.089	-19.3669	139.43
26SingA	3.353	0.002	1	2.642	1.673	0.429	1.419	0.687	2.336	3.129	6.354	3.4229	70.22
27SingA	3.376	0.013	1	2.686	1.638	0.402	1.405	0.602	3.043	3.77	6.295	20.7825	54.36
28SingA	3.398	0.003	1	2.839	1.316	0.24	1.252	0.286	2.856	3.869	5.44	9.4922	53.54
29SingA	3.426	0.009	1	2.875	1.181	0.152	1.163	0.213	3.849	4.523	5.324	-38.3077	124.79
30SingA	3.46	0.004	1	2.864	1.199	0.166	1.181	0.219	3.749	4.567	5.251	1.1804	87.64
31SingA	3.474	0.017	1	2.94	1.087	0.08	1.085	0.074	3.321	4.376	5.224	29.2987	35.76
32SingA	3.497	0.001	1	2.858	1.271	0.212	1.224	0.25	3.289	4.365	5.763	3.4949	71.24
33SingA	3.507	0.001	1	2.813	1.312	0.234	1.246	0.347	1.453	2.04	6.003	0.5527	84.17
34SingA	3.52	0.006	1	2.905	1.133	0.116	1.124	0.148	3.724	4.676	5.43	7.7867	54.08
35SingA	3.52	0.001	1	2.879	1.219	0.178	1.188	0.21	2.985	4	5.656	16.4033	25.54
36SingA	3.542	0.006	1	2.663	1.771	0.471	1.45	0.651	1.484	2.107	6.592	-3.7238	102.08
37SingA	3.552	0.006	1	2.866	1.205	0.168	1.179	0.236	4.256	4.873	5.826	-2.0388	100.11
38SingA	3.573	0	1	2.93	1.087	0.08	1.084	0.104	2.439	3.609	5.371	-3.6904	121.76
39SingA	3.583	0.005	1	2.894	1.176	0.149	1.161	0.164	3.372	4.401	5.772	-15.9698	137.05
40SingA	3.589	0.005	1	2.949	1.066	0.062	1.066	0.027	4.356	5.143	5.379	-5.8004	95.83
41SingA	3.596	0.018	1	2.822	1.255	0.203	1.214	0.323	3.27	4.398	5.865	-4.518	96.98
42SingA	3.603	0.003	1	2.725	1.469	0.33	1.369	0.466	4.799	5.455	6.014	0.1778	90
43SingA	3.628	0.005	1	2.81	1.422	0.312	1.321	0.332	4.388	5.206	6.247	1.4446	81.21
44SingA	3.651	0.017	1	2.904	1.172	0.149	1.164	0.12	3.804	5.05	5.646	41.7097	64.54
45SingA	3.654	0.011	1	2.701	1.464	0.333	1.339	0.553	5.149	5.951	6.11	-14.8751	142.92
46SingA	3.669	0.005	1	2.74	1.558	0.484	1.375	0.488	1.775	2.7	6.605	7.4334	32.28
47SingA	3.673	0.007	1	2.736	1.381	0.288	1.3	0.475	4.452	5.292	6.016	28.1832	55.41
48SingA	3.686	0.002	1	2.688	1.689	0.43	1.438	0.582	2.064	3.052	6.531	-13.0917	126.64
49SingA	3.696	0.004	1	2.625	1.507	0.392	1.36	0.705	2.737	3.604	6.217	-18.0023	171.33
50SingA	3.715	0.006	1	2.851	1.229	0.185	1.201	0.247	4.434	5.528	5.789	-3.106	100.99

trans-3

(*S,S*, ^f,s^C)34,35-bis[60]PCBM

state	dE(eV)	f	Om	POS	PR	CT	COH	CTnt	PRNTO	Z_HE	RMSeh	R (velocity)	E-M angle
1SingA	1.89	0.002	1.001	2.967	1.046	0.044	1.045	0.051	1.009	1.038	5.038	42.3862	70.45
2SingA	2.062	0.004	1.001	2.969	1.043	0.041	1.042	0.034	1.538	1.764	5.054	-28.2312	130.01
3SingA	2.122	0.001	1.001	2.988	1.017	0.016	1.017	0	1.332	1.573	4.97	-100.125	164.99
4SingA	2.156	0.001	1.001	2.989	1.015	0.015	1.015	-0.002	1.052	1.155	4.96	41.3466	90
5SingA	2.268	0.001	1.001	2.991	1.012	0.012	1.012	-0.006	1.152	1.372	4.959	-18.0745	120.95
6SingA	2.271	0.006	1.001	2.975	1.034	0.032	1.033	0.008	2.597	2.863	4.928	72.1866	0.53
7SingA	2.295	0.001	1.001	2.979	1.029	0.028	1.029	0.028	1.266	1.548	4.968	-87.7459	90
8SingA	2.383	0.009	1.002	2.957	1.059	0.055	1.058	0.01	1.746	2.133	5.08	150.8924	90
9SingA	2.396	0.001	1.001	2.981	1.026	0.025	1.026	0.024	1.142	1.378	4.995	-24.0358	115.32
10SingA	2.47	0.004	1.001	2.975	1.033	0.032	1.033	0.004	1.368	1.648	4.945	90.487	38.76
11SingA	2.482	0.003	1.002	2.973	1.037	0.036	1.037	0.014	1.431	1.83	4.997	27.599	90
12SingA	2.576	0.004	1.002	2.973	1.037	0.036	1.036	-0.038	1.317	1.619	5.02	-78.5818	179.81
13SingA	2.58	0.004	1.001	2.969	1.043	0.041	1.043	-0.028	1.415	1.734	5.029	11.9804	84.65
14SingA	2.726	0.009	1.001	2.954	1.063	0.059	1.063	-0.012	1.089	1.262	5.075	-46.3697	104.44
15SingA	2.764	0.008	1.001	2.959	1.057	0.054	1.057	-0.017	1.062	1.194	5.041	-144.398	179.85
16SingA	3.056	0.012	1	2.911	1.137	0.118	1.123	0.162	1.38	1.838	5.032	36.9099	70.22
17SingA	3.108	0.025	1.001	2.966	1.048	0.046	1.047	0.056	1.278	1.706	4.969	-35.2232	169.73
18SingA	3.117	0.007	1	2.908	1.141	0.121	1.127	0.162	2.348	2.739	4.761	-9.4554	90
19SingA	3.189	0.004	1	2.928	1.106	0.094	1.099	0.121	1.792	2.366	4.962	17.7987	65.22
20SingA	3.223	0	1	2.819	1.323	0.24	1.252	0.342	2.049	2.271	6.018	-2.0023	90
21SingA	3.296	0.003	1	2.805	1.353	0.257	1.27	0.366	1.809	2.655	5.503	-2.4728	134.83
22SingA	3.299	0.001	1	2.86	1.231	0.184	1.195	0.249	1.884	2.565	5.485	-8.7119	90
23SingA	3.311	0.004	1	2.929	1.105	0.094	1.097	0.122	2.569	3.216	5.124	-2.3954	90
24SingA	3.351	0.017	1	2.86	1.233	0.185	1.196	0.256	3.392	3.909	5.335	-31.9067	90
25SingA	3.377	0.019	1.001	2.917	1.124	0.108	1.115	0.126	3.749	4.374	5.14	15.6649	80.82
26SingA	3.407	0.01	1	2.911	1.125	0.109	1.115	0.146	3.197	4.077	5.219	-7.1509	164.22
27SingA	3.407	0.002	1	2.845	1.273	0.211	1.226	0.278	3.102	4.063	5.333	-8.3673	158.53
28SingA	3.419	0.015	1	2.954	1.065	0.06	1.062	0.063	3.273	4.115	4.956	37.8847	17.8
29SingA	3.422	0	1	2.778	1.421	0.293	1.305	0.422	2.179	2.723	5.962	-0.017	90
30SingA	3.442	0	1	2.866	1.219	0.176	1.187	0.24	4.426	4.955	5.497	-0.7101	97.22
31SingA	3.474	0	1	2.903	1.146	0.127	1.136	0.143	4.374	4.95	5.173	1.708	90
32SingA	3.486	0.013	1	2.842	1.268	0.207	1.22	0.284	2.794	3.642	5.493	-11.0536	103.17
33SingA	3.501	0.004	1	2.902	1.149	0.127	1.136	0.147	2.654	3.176	5.441	0.0977	90
34SingA	3.519	0.008	1	2.868	1.214	0.174	1.185	0.231	3.316	3.944	5.847	5.5227	67.16
35SingA	3.535	0.009	1	2.663	1.726	0.443	1.438	0.643	2.764	3.528	6.668	-9.4317	97.89
36SingA	3.559	0.02	1	2.85	1.251	0.198	1.212	0.264	2.163	3.306	5.694	-18.7684	90
37SingA	3.585	0.004	1	2.784	1.374	0.27	1.299	0.36	3.412	4.476	6.039	-8.8411	122.44
38SingA	3.585	0.011	1	2.66	1.749	0.455	1.459	0.635	2.995	3.754	6.641	0.8613	71.41
39SingA	3.615	0.005	1	2.849	1.256	0.2	1.211	0.282	2.48	3.01	6.008	29.1361	1.56
40SingA	3.618	0	1	2.824	1.307	0.232	1.248	0.318	2.371	3.225	5.803	3.5091	90
41SingA	3.625	0.018	1	2.856	1.239	0.188	1.2	0.256	1.926	2.781	5.727	38.8719	11.55
42SingA	3.646	0.026	1	2.727	1.55	0.36	1.379	0.505	2.876	3.264	6.305	22.9108	43.14
43SingA	3.648	0.001	1	2.722	1.566	0.366	1.376	0.527	3.643	4.303	6.27	10.5474	1.62
44SingA	3.654	0.001	1	2.719	1.564	0.37	1.398	0.505	2.827	3.713	6.253	-0.6097	90
45SingA	3.68	0	1	2.921	1.115	0.103	1.111	0.098	4.181	5.101	5.356	4.7095	2.84
46SingA	3.685	0.038	1	2.792	1.378	0.272	1.295	0.362	3.928	5.052	6.028	40.9491	66.19
47SingA	3.701	0.015	1	2.786	1.387	0.276	1.303	0.359	3.807	4.652	5.88	4.9367	85.99
48SingA	3.713	0.006	1	2.832	1.287	0.219	1.238	0.287	3.192	3.804	6.109	37.4847	51.8
49SingA	3.72	0.004	1	2.806	1.338	0.245	1.274	0.306	3.299	3.892	5.847	-4.7746	90
50SingA	3.742	0.005	1	2.702	1.601	0.385	1.426	0.51	4.11	4.578	6.332	15.7966	1.51

trans-3

(*S,S,* f,s*A*)34,35-bis[60]PCBM

state	dE(eV)	f	Om	POS	PR	CT	COH	CTnt	PRNTO	Z_HE	RMSeh	R (velocity)	E-M angle
1SingA	1.908	0.002	1.001	2.972	1.039	0.037	1.038	0.04	1.009	1.038	5.001	-51.5279	114.63
2SingA	2.08	0.002	1	2.982	1.024	0.024	1.024	0.012	2.009	2.066	4.969	-22.5795	154.49
3SingA	2.131	0.002	1.001	2.979	1.029	0.028	1.029	0.013	1.652	1.88	5.021	142.8775	10.44
4SingA	2.131	0.001	1.001	2.981	1.025	0.024	1.025	0.014	1.062	1.188	5.01	-39.5687	155.22
5SingA	2.259	0.001	1.001	2.974	1.036	0.034	1.035	0.023	1.159	1.338	4.957	-65.7976	179.5
6SingA	2.271	0.003	1	2.975	1.034	0.033	1.034	0.029	1.226	1.446	4.982	104.2954	1.15
7SingA	2.272	0	1.001	2.991	1.013	0.012	1.013	-0.007	1.184	1.427	4.951	14.0991	57.65
8SingA	2.385	0.001	1.001	2.979	1.028	0.028	1.028	0.027	1.169	1.429	5.01	19.2455	68.99
9SingA	2.406	0.009	1.002	2.96	1.055	0.052	1.055	-0.002	1.46	1.79	5.053	-159.3349	179.85
10SingA	2.471	0.005	1	2.972	1.038	0.037	1.038	0.004	1.631	1.895	4.961	-101.8621	148.16
11SingA	2.483	0.002	1.002	2.973	1.037	0.036	1.037	0.012	1.487	1.876	5	-19.6986	178.1
12SingA	2.561	0.008	1.002	2.963	1.051	0.049	1.051	-0.007	1.681	1.973	5.065	-22.2024	97.1
13SingA	2.58	0.005	1.002	2.973	1.038	0.036	1.037	-0.036	1.348	1.655	5.025	82.4112	0.43
14SingA	2.722	0.007	1.001	2.957	1.06	0.057	1.06	-0.015	1.09	1.264	5.065	67.2528	66.3
15SingA	2.76	0.007	1.001	2.957	1.06	0.056	1.06	-0.01	1.062	1.192	5.052	136.3257	0.51
16SingA	3.051	0.011	1	2.936	1.094	0.085	1.088	0.112	1.321	1.728	4.975	-2.1822	91.31
17SingA	3.13	0.024	1	2.961	1.055	0.052	1.053	0.065	1.445	2.013	4.912	39.3969	13.34
18SingA	3.155	0.004	1	2.967	1.045	0.043	1.044	0.037	2.118	2.729	4.495	5.7522	2.62
19SingA	3.182	0.003	1	2.937	1.092	0.083	1.086	0.103	1.911	2.45	4.919	-26.0096	126.93
20SingA	3.27	0.012	1	2.908	1.141	0.121	1.127	0.165	2.939	3.333	5.107	28.2739	90
21SingA	3.309	0.007	1	2.828	1.302	0.228	1.242	0.315	1.987	2.812	5.335	8.393	71.15
22SingA	3.311	0	1	2.955	1.063	0.059	1.061	0.064	2.637	3.063	4.97	0.1384	90
23SingA	3.312	0.001	1	2.631	1.81	0.486	1.459	0.719	1.442	1.94	6.672	-1.0052	90
24SingA	3.37	0.032	1.001	2.907	1.141	0.122	1.129	0.153	3.27	4	5.158	11.4256	84.68
25SingA	3.372	0.011	1	2.906	1.145	0.124	1.13	0.162	3.567	4.068	5.164	9.7867	6.5
26SingA	3.397	0.007	1	2.812	1.34	0.25	1.262	0.356	3.374	3.969	5.724	14.4492	4.35
27SingA	3.4	0.012	1	2.95	1.071	0.065	1.068	0.069	2.24	3.088	5.171	-13.9037	176
28SingA	3.414	0.007	1	2.918	1.122	0.107	1.112	0.136	3.995	4.646	5.317	-19.2454	178.03
29SingA	3.423	0.009	1	2.913	1.132	0.114	1.121	0.14	2.439	3.336	5.331	22.0314	1.88
30SingA	3.433	0	1	2.723	1.562	0.364	1.376	0.521	2.386	3.22	6.095	-0.1749	90
31SingA	3.461	0.011	1	2.927	1.107	0.095	1.099	0.123	2.976	3.739	5.316	-33.6773	145.17
32SingA	3.478	0.018	1	2.778	1.417	0.29	1.306	0.411	3.048	3.704	5.974	31.1267	28.82
33SingA	3.494	0.008	1	2.877	1.188	0.158	1.175	0.171	1.942	2.67	5.378	3.6101	10.27
34SingA	3.496	0.01	1	2.721	1.574	0.371	1.38	0.53	2.458	3.525	6.007	-5.4567	101.59
35SingA	3.505	0.001	1	2.835	1.285	0.217	1.229	0.303	2.284	3.256	5.915	3.3465	90
36SingA	3.539	0.003	1	2.755	1.48	0.325	1.338	0.465	2.056	3.072	6.109	-26.5144	141.67
37SingA	3.552	0	1	2.784	1.405	0.285	1.298	0.408	1.442	2.052	6.174	-0.4218	90
38SingA	3.562	0.003	1	2.926	1.109	0.097	1.102	0.116	2.999	3.784	5.591	6.9797	54.18
39SingA	3.621	0.018	1	2.876	1.196	0.163	1.176	0.196	3.676	4.659	5.298	-23.6991	179.51
40SingA	3.623	0.029	1.001	2.946	1.076	0.07	1.074	0.049	4.183	4.833	5.414	-27.308	121.69
41SingA	3.63	0.004	1	2.655	1.746	0.456	1.456	0.652	2.808	3.517	6.517	8.4856	2.17
42SingA	3.644	0.001	1	2.696	1.635	0.402	1.414	0.57	2.977	3.625	6.307	-11.0735	170.51
43SingA	3.656	0.024	1	2.734	1.525	0.345	1.364	0.481	3.467	4.411	5.998	-48.0915	138.08
44SingA	3.658	0.001	1	2.821	1.308	0.235	1.261	0.298	4.534	5.233	5.78	-4.8236	177.17
45SingA	3.676	0.004	1	2.587	1.905	0.531	1.514	0.764	3.004	3.693	6.806	3.8516	17.78
46SingA	3.681	0	1	2.767	1.434	0.303	1.337	0.394	5.225	5.72	6.166	-12.3862	176.58
47SingA	3.697	0.028	1	2.88	1.187	0.156	1.17	0.181	2.74	3.782	5.51	-157.2936	175.89
48SingA	3.703	0.004	1	2.695	1.628	0.395	1.417	0.548	4.001	4.563	6.237	-6.8308	178.17
49SingA	3.718	0.011	1	2.759	1.459	0.313	1.34	0.424	4.13	4.887	6.195	29.4014	53.42
50SingA	3.733	0.007	1	2.82	1.314	0.233	1.252	0.31	2.68	3.619	6.032	-26.845	121.58

trans-3

(S,R, $^{f,s}C$)34,35-bis[60]PCBM

state	dE(eV)	f	Om	POS	PR	CT	COH	CTnt	PRNTO	Z_HE	RMSeh	R (velocity)	E-M angle
1SingA	1.898	0.002	1	2.861	1.154	0.134	1.152	-0.076	1.009	1.037	5.023	47.4034	67.78
2SingA	2.071	0.003	1	2.79	1.236	0.189	1.233	-0.061	1.836	1.973	5.017	-2.0686	95.3
3SingA	2.124	0.002	1.001	2.734	1.302	0.232	1.301	-0.033	1.522	1.765	4.997	-121.605	166.29
4SingA	2.144	0.001	1.001	2.77	1.26	0.203	1.256	-0.012	1.059	1.178	4.984	39.2888	24.06
5SingA	2.266	0.003	1	2.761	1.27	0.21	1.266	-0.04	1.57	1.999	4.934	66.717	35.9
6SingA	2.27	0.001	1.001	2.745	1.289	0.221	1.283	0.078	1.498	1.875	4.952	-6.5898	96.41
7SingA	2.28	0.002	1.001	2.829	1.19	0.159	1.189	0.015	1.305	1.629	4.99	-103.646	173.83
8SingA	2.39	0.003	1.001	2.765	1.269	0.212	1.261	0.145	1.367	1.737	5.012	-14.4438	99.99
9SingA	2.393	0.008	1.002	2.853	1.163	0.136	1.157	-0.052	1.801	2.169	5.062	147.6472	7.68
10SingA	2.469	0.004	1	2.699	1.35	0.257	1.347	-0.016	1.456	1.73	4.963	90.2926	33.62
11SingA	2.483	0.002	1.002	2.782	1.248	0.203	1.24	-0.188	1.445	1.811	4.988	27.0231	37.34
12SingA	2.569	0.006	1.001	2.787	1.24	0.197	1.244	0.009	1.486	1.802	5.048	5.7452	87.79
13SingA	2.58	0.004	1.002	2.742	1.293	0.231	1.295	0.084	1.348	1.664	5.025	-63.7882	135.83
14SingA	2.723	0.008	1.001	2.822	1.199	0.166	1.198	-0.033	1.089	1.261	5.072	-61.4279	110.39
15SingA	2.763	0.007	1.001	2.77	1.262	0.206	1.258	0.077	1.061	1.19	5.047	-137.687	167.26
16SingA	3.053	0.011	1	2.768	1.302	0.224	1.288	0.071	1.341	1.769	5.035	19.2199	78.79
17SingA	3.117	0.023	1	2.827	1.199	0.166	1.195	-0.116	1.376	1.875	4.921	-42.349	169.9
18SingA	3.137	0.007	1	2.817	1.226	0.181	1.221	-0.068	2.197	2.752	4.643	-0.3199	90.99
19SingA	3.185	0.004	1	2.745	1.314	0.229	1.301	0.029	1.873	2.485	4.94	18.5837	64.43
20SingA	3.249	0	1	2.719	1.483	0.33	1.432	0.168	2.07	2.492	5.898	-2.8592	133.17
21SingA	3.288	0.012	1	2.727	1.387	0.28	1.377	0.122	2.745	3.353	5.193	-24.4412	150.79
22SingA	3.311	0.001	1	2.704	1.392	0.27	1.359	0.19	2.907	3.528	4.94	-2.2366	111.92
23SingA	3.341	0.006	1	2.707	1.468	0.323	1.443	0.07	1.688	2.347	5.673	2.4756	83.34
24SingA	3.35	0.012	1	2.513	1.943	0.525	1.689	0.555	1.724	2.441	6.481	-13.5902	112.57
25SingA	3.366	0.013	1	2.768	1.288	0.221	1.284	0.026	3.885	4.584	4.758	-18.3041	133.4
26SingA	3.381	0.013	1.001	2.694	1.403	0.287	1.395	0.117	3.206	3.844	5.194	5.5787	84.49
27SingA	3.408	0.01	1	2.7	1.403	0.283	1.391	0.056	3.104	4.044	5.381	-0.4992	92.19
28SingA	3.413	0.008	1	2.731	1.342	0.242	1.32	0.106	3.101	4.113	5.032	6.985	71.54
29SingA	3.419	0.006	1	2.673	1.582	0.368	1.483	0.271	3.176	4.068	5.743	20.7678	52.27
30SingA	3.443	0.001	1	2.705	1.388	0.285	1.385	0.121	3.556	4.274	5.401	-2.1267	108.72
31SingA	3.465	0.001	1	2.581	1.651	0.393	1.539	0.392	2.834	3.586	5.809	2.5905	66.84
32SingA	3.487	0.01	1	2.732	1.392	0.278	1.368	0.108	3.633	4.383	5.508	-6.2793	105.98
33SingA	3.499	0.008	1	2.653	1.469	0.322	1.428	0.276	2.421	3.4	5.614	-14.9027	116.08
34SingA	3.501	0.017	1	2.765	1.343	0.264	1.349	0.063	4.954	5.51	5.522	0.5604	89.13
35SingA	3.528	0.012	1	2.686	1.556	0.363	1.482	0.191	3.381	4.324	5.917	17.1394	56.88
36SingA	3.541	0.003	1	2.681	1.468	0.327	1.436	0.242	2.2	3.204	5.872	-4.1046	104.42
37SingA	3.578	0.001	1	2.553	1.864	0.597	1.661	0.453	2.102	2.876	6.756	0.3553	87.31
38SingA	3.589	0.007	1	2.75	1.409	0.302	1.395	0.133	3.305	4.258	5.887	-5.173	102
39SingA	3.605	0.007	1	2.709	1.472	0.322	1.425	0.182	5.256	5.644	5.946	3.6264	80.41
40SingA	3.616	0.01	1	2.611	1.638	0.403	1.541	0.38	3.34	4.399	5.951	7.7131	71.56
41SingA	3.625	0.002	1	2.609	1.666	0.425	1.617	0.295	2.62	3.537	5.864	3.3312	72.39
42SingA	3.637	0.013	1	2.613	1.649	0.402	1.574	0.287	3.675	4.391	5.992	34.8409	5.04
43SingA	3.656	0.033	1	2.602	1.784	0.462	1.601	0.416	3.441	4.198	6.213	41.7044	57.35
44SingA	3.661	0.006	1	2.644	1.605	0.375	1.494	0.359	3.838	4.704	6.077	5.3086	49.68
45SingA	3.681	0.02	1	2.644	1.553	0.346	1.465	0.331	4.77	5.506	5.74	81.362	29.4
46SingA	3.686	0.001	1	2.59	1.696	0.406	1.539	0.421	3.357	4.203	6.08	1.223	82.91
47SingA	3.698	0.017	1	2.605	1.726	0.44	1.607	0.353	4.538	5.299	6.204	-14.6094	121.33
48SingA	3.709	0.003	1	2.707	1.394	0.291	1.38	0.21	3.23	4.216	5.659	40.5064	34.82
49SingA	3.722	0.007	1	2.635	1.691	0.439	1.602	0.263	3.071	4.155	6.173	29.1233	19.03
50SingA	3.729	0.01	1	2.583	1.652	0.397	1.528	0.433	3.636	4.333	6.061	13.635	70.09

trans-2

(*S*,*S*, ^{f,s}*C*)49,59-bis[60]PCBM

state	dE(eV)	f	Om	POS	PR	CT	COH	CTnt	PRNTO	Z_HE	RMSeh	R (velocity)	E-M angle
1SingA	1.898	0.004	1.001	2.978	1.03	0.029	1.03	0.026	1.01	1.042	4.974	39.3217	76.71
2SingA	1.942	0.001	1.001	2.979	1.029	0.028	1.028	0.021	1.047	1.135	4.972	-51.273	90
3SingA	2.033	0	1.001	2.994	1.008	0.008	1.008	-0.008	1.037	1.121	4.914	4.2221	74.97
4SingA	2.174	0.002	1.001	2.97	1.042	0.04	1.041	0.029	1.586	1.908	4.955	-49.5387	90
5SingA	2.188	0.003	1.001	2.976	1.032	0.031	1.032	0.015	1.092	1.24	4.972	-81.5395	123.03
6SingA	2.24	0	1.001	2.983	1.023	0.023	1.023	0.006	2.135	2.314	5.011	6.2382	90
7SingA	2.35	0	1.003	2.985	1.02	0.019	1.02	0	1.826	2.105	5.053	-0.9223	90
8SingA	2.386	0.002	1	2.966	1.047	0.045	1.047	0.013	2.42	2.776	4.998	63.7776	36.52
9SingA	2.419	0.005	1	2.975	1.034	0.033	1.034	0.01	1.894	2.362	4.964	118.9664	9.68
10SingA	2.489	0.006	1.002	2.961	1.054	0.051	1.054	0.037	2.237	2.557	5.14	65.5016	33.69
11SingA	2.524	0.004	1.002	2.964	1.049	0.047	1.049	0.001	1.203	1.485	5.105	40.0947	90
12SingA	2.574	0.002	1.002	2.978	1.029	0.029	1.029	-0.024	1.344	1.743	5.043	-23.1077	114.23
13SingA	2.623	0.001	1	2.961	1.054	0.051	1.054	0.029	1.717	1.921	5.077	2.6048	90
14SingA	2.669	0	1.001	2.97	1.041	0.04	1.042	-0.003	1.696	1.928	4.992	-30.3723	90
15SingA	2.867	0.012	1	2.948	1.073	0.068	1.073	0.028	1.053	1.177	4.953	-84.1302	112.15
16SingA	2.994	0.016	1.001	2.939	1.088	0.08	1.083	0.101	1.379	1.833	4.981	-63.6659	125.83
17SingA	3.134	0	1	2.962	1.053	0.05	1.051	0.06	2.27	2.598	4.665	-3.4044	90
18SingA	3.163	0.011	1	2.926	1.111	0.098	1.101	0.135	1.496	1.928	5.145	-22.2733	157.64
19SingA	3.177	0.003	1	2.929	1.104	0.093	1.098	0.107	1.324	1.743	4.88	34.4611	90
20SingA	3.219	0.026	1	2.633	1.807	0.484	1.461	0.711	1.443	1.926	6.534	-4.6858	134.72
21SingA	3.258	0.017	1	2.963	1.051	0.048	1.05	0.048	2.421	2.856	4.789	50.5059	90
22SingA	3.294	0.008	1.001	2.954	1.065	0.06	1.063	0.066	2.934	3.721	4.598	-16.6995	124.94
23SingA	3.312	0.003	1	2.964	1.051	0.048	1.05	0.045	2.918	3.668	4.609	10.8156	90
24SingA	3.345	0.003	1	2.695	1.641	0.402	1.404	0.585	2.488	3.377	6.216	-8.741	90
25SingA	3.35	0.014	1	2.98	1.027	0.026	1.026	0.01	3.121	3.971	4.71	-30.7053	134.96
26SingA	3.379	0.002	1	2.695	1.643	0.402	1.404	0.587	3.181	3.717	6.147	-9.1097	90
27SingA	3.387	0.013	1	2.909	1.136	0.119	1.129	0.13	4.105	4.899	4.952	5.4159	83.25
28SingA	3.41	0	1	2.67	1.709	0.433	1.43	0.63	1.938	2.568	6.325	0.0952	90
29SingA	3.435	0.004	1	2.875	1.203	0.166	1.175	0.225	2.505	3.313	5.622	14.4345	90
30SingA	3.453	0.005	1	2.888	1.175	0.147	1.158	0.181	3.091	3.76	5.309	-38.3238	174.48
31SingA	3.455	0.015	1	2.856	1.238	0.189	1.201	0.254	3.593	4.064	5.704	-18.1039	170.19
32SingA	3.472	0	1	2.833	1.286	0.22	1.236	0.295	3.78	4.401	6.028	-1.9351	120.59
33SingA	3.499	0.023	1	2.961	1.053	0.051	1.053	0.024	3.709	4.358	5.234	33.8239	90
34SingA	3.519	0.003	1	2.958	1.059	0.055	1.058	0.043	3.347	3.912	5.582	4.734	90
35SingA	3.542	0	1	2.289	1.628	0.938	1.396	1.401	1.033	1.11	7.868	-2.0338	90
36SingA	3.547	0.005	1	2.925	1.111	0.099	1.105	0.113	3.158	3.93	5.646	-22.4536	166.48
37SingA	3.552	0.002	1	2.702	1.616	0.391	1.405	0.552	3.853	4.631	5.841	1.8669	73.26
38SingA	3.557	0.001	1	2.885	1.181	0.152	1.163	0.186	4.274	4.929	5.846	-3.6022	99.05
39SingA	3.589	0.032	1	2.688	1.654	0.406	1.417	0.583	3.665	4.562	6.29	9.0077	67.23
40SingA	3.605	0.009	1	2.818	1.321	0.241	1.258	0.329	3.071	3.911	5.861	6.2541	23.41
41SingA	3.608	0	1	2.957	1.059	0.056	1.059	0.026	2.156	2.946	5.273	-1.2179	90
42SingA	3.618	0.011	1	2.794	1.374	0.271	1.296	0.362	4.644	5.447	5.986	-12.6388	122.61
43SingA	3.625	0	1	2.841	1.269	0.209	1.225	0.275	1.489	2.075	5.703	6.8459	90
44SingA	3.658	0.004	1	2.934	1.096	0.087	1.091	0.095	1.485	2.089	5.4	-2.0148	90
45SingA	3.675	0.001	1	2.886	1.176	0.146	1.161	0.156	1.936	2.84	5.424	6.3895	90
46SingA	3.676	0.002	1	2.685	1.653	0.403	1.427	0.562	2.884	4.038	6.405	2.1983	61.74
47SingA	3.69	0.005	1	2.854	1.241	0.189	1.203	0.246	3.514	4.477	5.694	2.8862	75.32
48SingA	3.721	0.002	1	2.818	1.318	0.238	1.257	0.317	2.302	2.85	6.135	24.4034	90
49SingA	3.731	0.008	1	2.306	1.693	0.907	1.429	1.349	1.155	1.433	7.531	11.5168	54.58
50SingA	3.733	0.035	1.001	2.821	1.305	0.234	1.263	0.281	4.116	5.278	5.5	170.4294	0.16

trans-2

(*R*,*S*, $^{f,s}A$)49,59-bis[60]PCBM

state	dE(eV)	f	Om	POS	PR	CT	COH	CTnt	PRNTO	Z_HE	RMSeh	R (velocity)	E-M angle
1SingA	1.874	0.004	1.001	2.7	1.349	0.252	1.338	-0.029	1.01	1.041	5.021	-39.5647	103.13
2SingA	1.93	0.001	1	2.666	1.389	0.27	1.374	0.036	1.05	1.14	4.985	53.2035	13.1
3SingA	2.028	0	1.001	2.693	1.353	0.256	1.346	0.015	1.041	1.13	4.917	-4.4506	110.77
4SingA	2.153	0.002	1.001	2.765	1.269	0.205	1.257	0.076	1.382	1.675	5.003	67.3584	6.66
5SingA	2.178	0.002	1	2.712	1.331	0.243	1.314	0.154	1.097	1.249	4.986	84.9632	52.35
6SingA	2.232	0	1.001	2.78	1.248	0.196	1.241	-0.087	2.111	2.329	5.027	-7.5727	146.61
7SingA	2.346	0	1.002	2.743	1.292	0.226	1.29	0.053	1.831	2.118	5.058	-1.0457	101.49
8SingA	2.371	0.003	1	2.762	1.272	0.208	1.26	0.103	1.874	2.331	5.05	-92.4559	156.93
9SingA	2.416	0.005	1	2.817	1.203	0.163	1.195	-0.041	1.624	2.091	4.966	-120.334	164.34
10SingA	2.478	0.005	1.002	2.766	1.268	0.206	1.26	-0.038	2.22	2.602	5.156	-37.1759	132.06
11SingA	2.514	0.005	1.002	2.742	1.295	0.225	1.278	0.177	1.225	1.526	5.118	-41.7969	172
12SingA	2.567	0.002	1.002	2.783	1.243	0.191	1.234	0.089	1.368	1.776	5.047	23.7188	67.17
13SingA	2.621	0.001	1	2.83	1.189	0.151	1.179	-0.019	1.708	1.911	5.096	-3.3627	166.13
14SingA	2.662	0	1.001	2.844	1.172	0.14	1.164	0.003	1.685	1.916	5.005	24.6303	29.4
15SingA	2.864	0.009	1	2.842	1.177	0.141	1.164	0.045	1.06	1.194	4.965	89.8174	62.89
16SingA	2.986	0.019	1	2.726	1.318	0.229	1.298	-0.083	1.391	1.867	4.974	54.0221	66.86
17SingA	3.102	0.001	1	2.659	1.441	0.281	1.399	0.03	2.054	2.453	4.817	-6.0618	125.62
18SingA	3.147	0.016	1	2.691	1.369	0.259	1.353	-0.032	1.259	1.643	5.012	22.1944	26.55
19SingA	3.175	0.004	1	2.779	1.251	0.19	1.236	-0.04	1.344	1.779	4.849	-38.561	168.66
20SingA	3.218	0.01	1	2.515	1.917	0.48	1.721	0.333	1.547	2.018	5.948	-13.0954	155.09
21SingA	3.244	0.015	1	2.607	1.524	0.339	1.49	0.196	2.239	2.653	5.397	-46.1815	154.05
22SingA	3.283	0.006	1	2.569	1.626	0.372	1.55	0.266	2.05	2.89	5.552	-7.0827	121.85
23SingA	3.296	0.012	1	2.552	1.568	0.343	1.498	0.308	2.362	3.202	5.336	34.564	39.57
24SingA	3.329	0.009	1	2.611	1.615	0.375	1.566	0.108	3.657	4.411	5.253	15.5694	11.07
25SingA	3.35	0.011	1	2.541	1.707	0.396	1.613	0.261	2.652	3.368	5.346	-8.9442	104.64
26SingA	3.36	0.004	1	2.703	1.382	0.269	1.371	0.007	3.758	4.308	5.16	9.3735	30.84
27SingA	3.376	0.019	1	2.67	1.403	0.278	1.388	0.068	4.393	4.987	4.988	-1.7161	91.48
28SingA	3.409	0.001	1	2.689	1.395	0.279	1.385	0.063	3.465	4.142	5.098	-2.9404	128.25
29SingA	3.429	0.009	1	2.684	1.459	0.312	1.436	0.113	3.165	4.038	5.671	20.7099	18.78
30SingA	3.445	0.005	1	2.575	1.673	0.41	1.582	0.353	4.068	4.599	6.071	13.1943	7.65
31SingA	3.449	0.001	1	2.484	1.688	0.44	1.572	0.558	3.181	4.068	6.26	2.5627	57.83
32SingA	3.472	0.013	1	2.601	1.62	0.376	1.552	0.227	3.549	4.288	5.718	34.5758	45.53
33SingA	3.486	0.019	1	2.652	1.442	0.317	1.431	0.184	3.741	4.562	5.526	-21.0408	129.42
34SingA	3.508	0.003	1	2.517	1.985	0.58	1.725	0.503	2.445	3.124	6.558	0.1225	90
35SingA	3.517	0.011	1	2.603	1.613	0.386	1.552	0.257	3.567	4.316	5.882	-3.2399	96.64
36SingA	3.532	0.01	1	2.467	1.738	0.45	1.641	0.478	2.996	4.299	6.251	-3.9821	98.33
37SingA	3.549	0.001	1	2.62	1.542	0.381	1.552	0.153	3.188	3.774	5.789	19.2179	30.64
38SingA	3.573	0.03	1	2.671	1.415	0.299	1.415	0.091	4.577	5.663	5.332	-8.3441	134.52
39SingA	3.576	0.003	1	2.399	2.047	0.573	1.801	0.598	2.306	3.457	6.538	12.7957	29.38
40SingA	3.596	0.013	1	2.719	1.374	0.26	1.352	0.071	3.667	4.625	5.333	-0.5992	95.9
41SingA	3.605	0	1	2.624	1.46	0.312	1.428	0.248	2.841	3.624	5.672	0.621	69.61
42SingA	3.614	0.002	1	2.54	1.893	0.49	1.694	0.36	2.839	3.904	6.268	10.9553	44.98
43SingA	3.634	0.003	1	2.648	1.47	0.312	1.42	0.258	2.014	3.127	5.375	-15.1416	136.81
44SingA	3.639	0	1	2.703	1.442	0.302	1.398	0.21	1.807	2.748	5.687	-3.1173	160.92
45SingA	3.681	0.017	1	2.653	1.494	0.34	1.471	0.211	3.371	4.434	5.667	-12.7433	114.39
46SingA	3.684	0.003	1	2.666	1.489	0.323	1.449	0.201	2.975	3.898	5.538	4.9891	53.02
47SingA	3.697	0.012	1	2.643	1.63	0.394	1.546	0.275	3.88	4.498	5.938	-38.3028	131.19
48SingA	3.708	0.013	1	2.548	1.684	0.437	1.573	0.478	5.18	5.908	6.151	-9.0275	135.19
49SingA	3.71	0.022	1	2.686	1.409	0.294	1.409	0.083	4.839	6	5.345	-129.423	155.69
50SingA	3.72	0.017	1	2.573	1.575	0.385	1.539	0.314	3.316	4.107	5.929	-3.062	101.13

Natural Transition Orbitals analysis

cis-2

(*S,S*)3,15-bis[60]PCBM

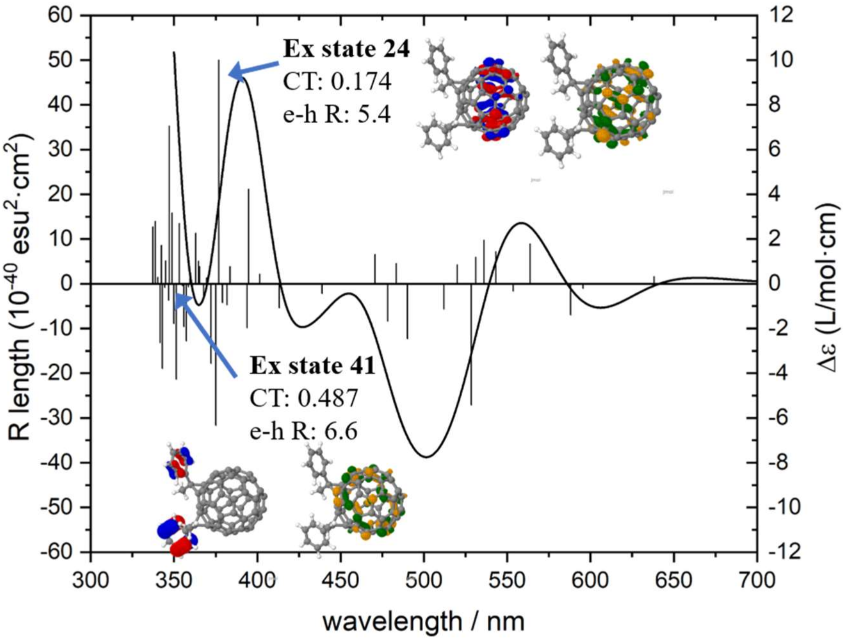

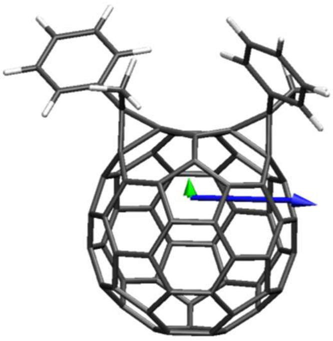

Ex state 24

|**μ**| = 0.08 a.u.

|**m**|= 0.42 a.u.

$\theta_{\mu,m}$= 40°

cis-3

(*S,R,* [f,s]*C*)13,14-bis[60]PCBM

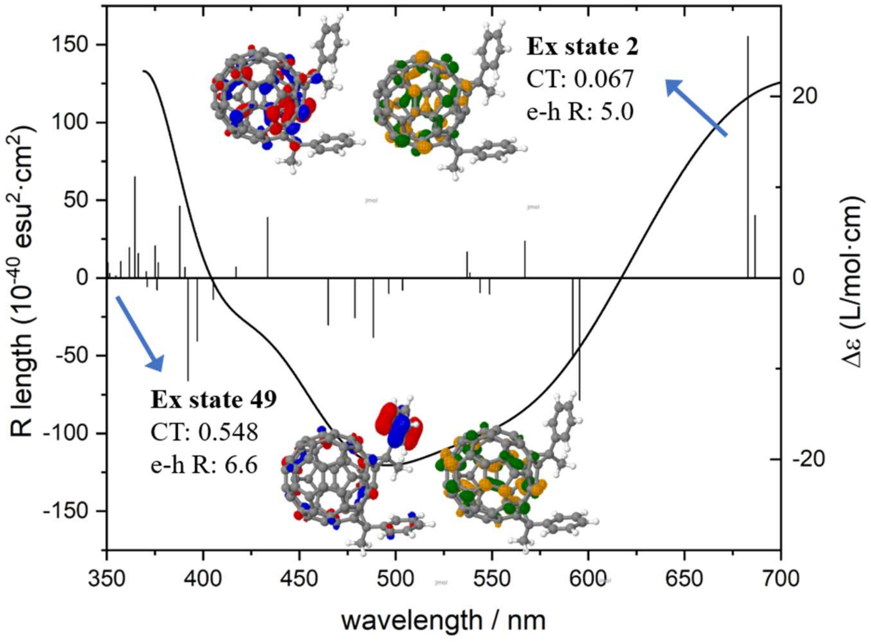

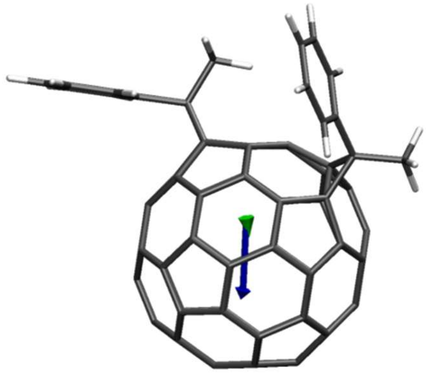

Ex state 2

|**μ**| = 0.02 a.u.

|**m**|= 2.24 a.u.

$\theta_{\mu,m}$= 1.7°

($S,S,$ $^{f,s}\underline{C}$)13,14-bis[60]PCBM

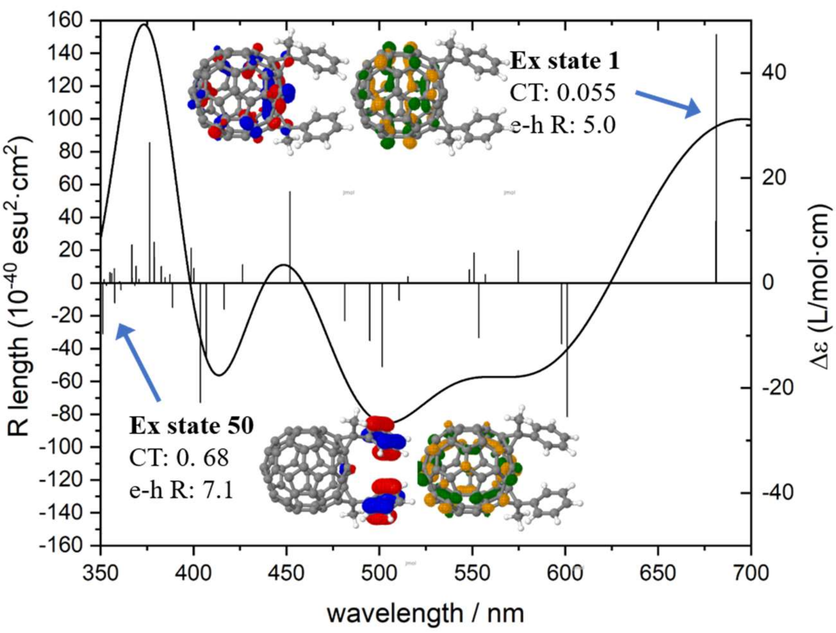

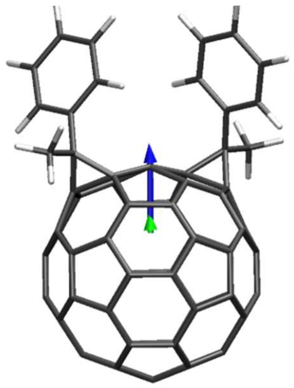

Ex state 1

|**μ**| = 0.02 a.u.

|**m**|= 2.23 a.u.

$\theta_{\mu,m}$= 1.3°

e

(*anti,S*)16,17-bis[60]PCBM

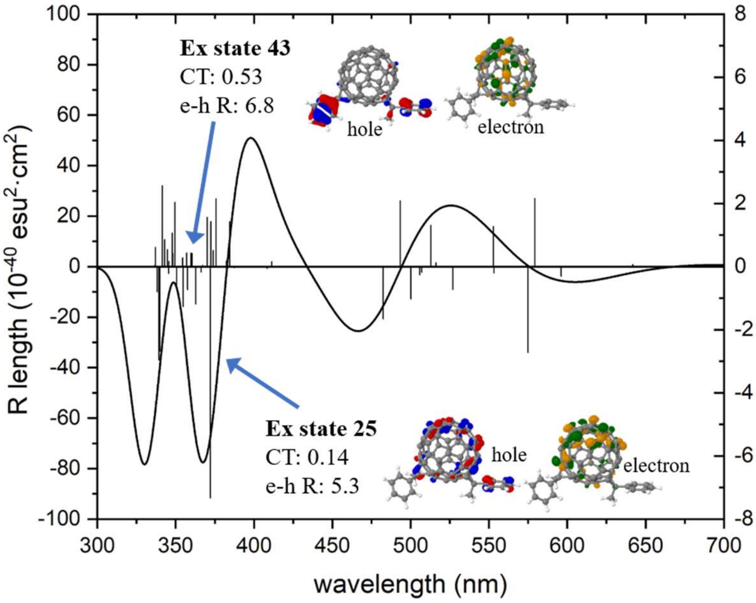

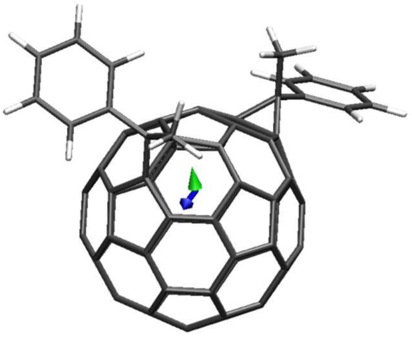

Ex state 25

|**μ**| = 0.05 a.u.

|**m**|= 1.05 a.u.

$\theta_{\mu,m}$=148.12°

trans-4

(*S,S*)32,33-bis[60]PCBM

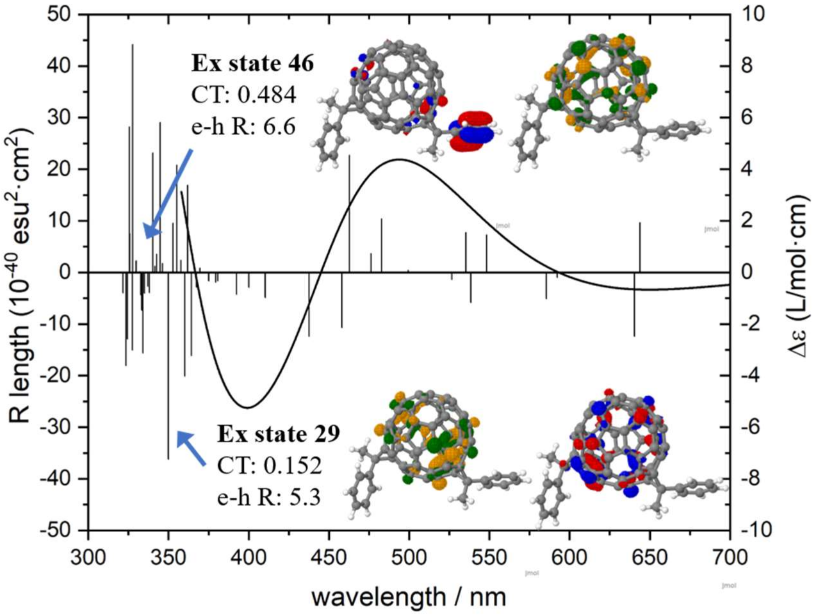

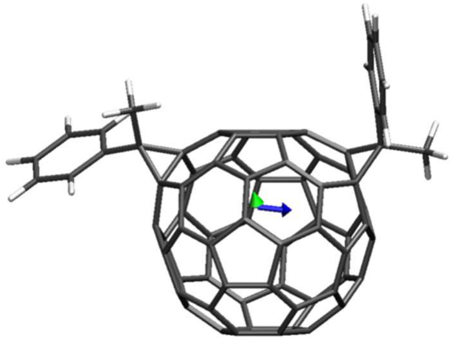

Ex state 29

|**μ**| = 0.04 a.u.

|**m**|= 0.83 a.u.

$\theta_{\mu,m}$= 125°

trans-3

(*S,S,* ^{f,s}<u>*C*</u>)34,35-bis[60]PCBM

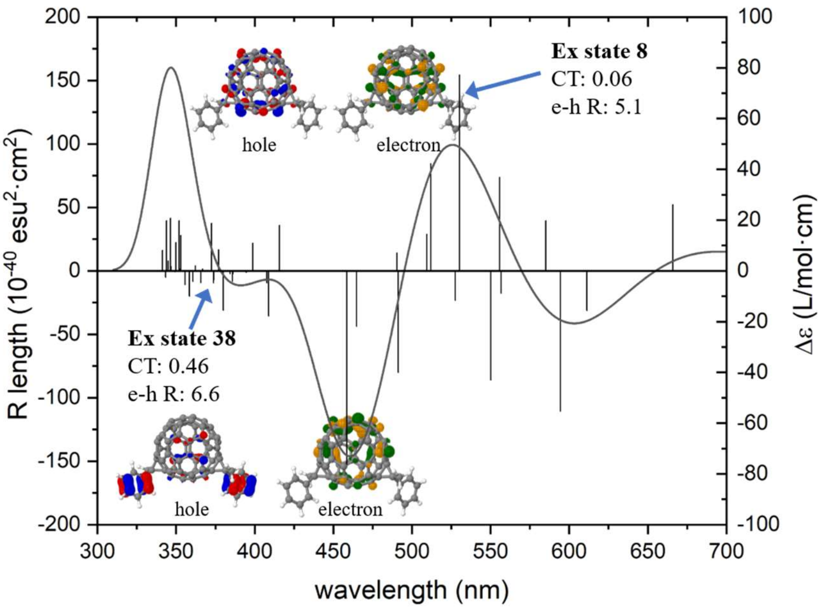

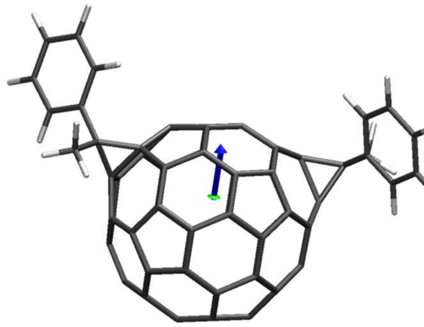

Ex state 8

|**μ**| = 0.03 a.u.

|**m**|= 1.64 a.u.

$\theta_{\mu,m}$= 0.036°

(*R*,*R*, f,s*C*)34,35-bis[60]PCBM

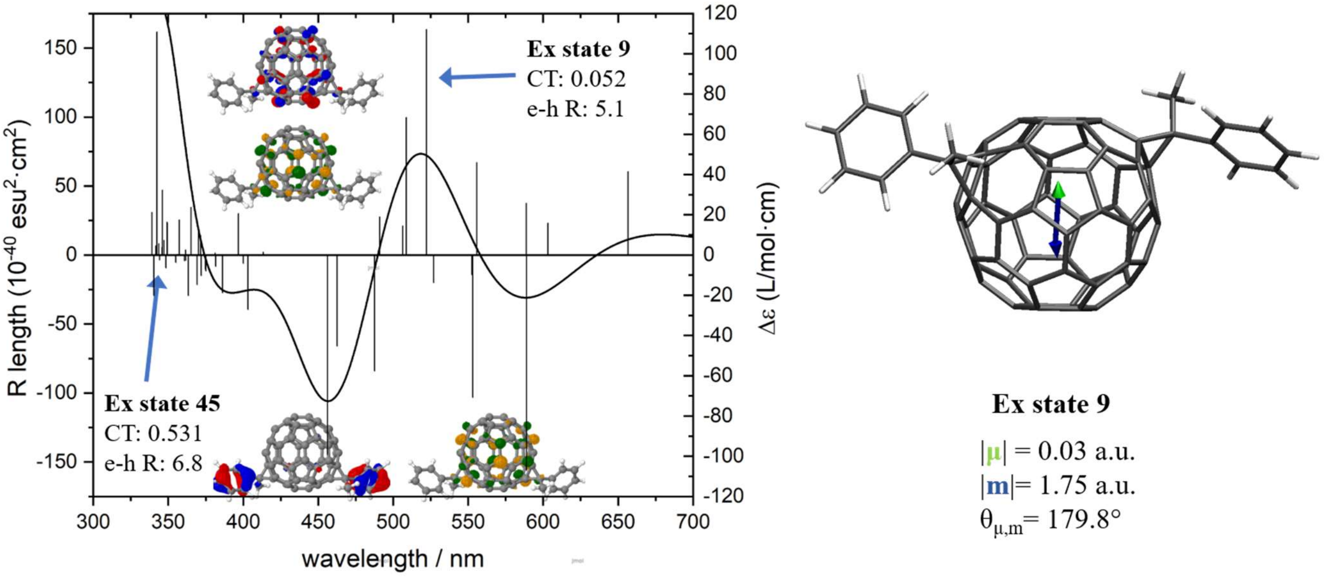

(*S*,*R*, f,s*C*)34,35-bis[60]PCBM

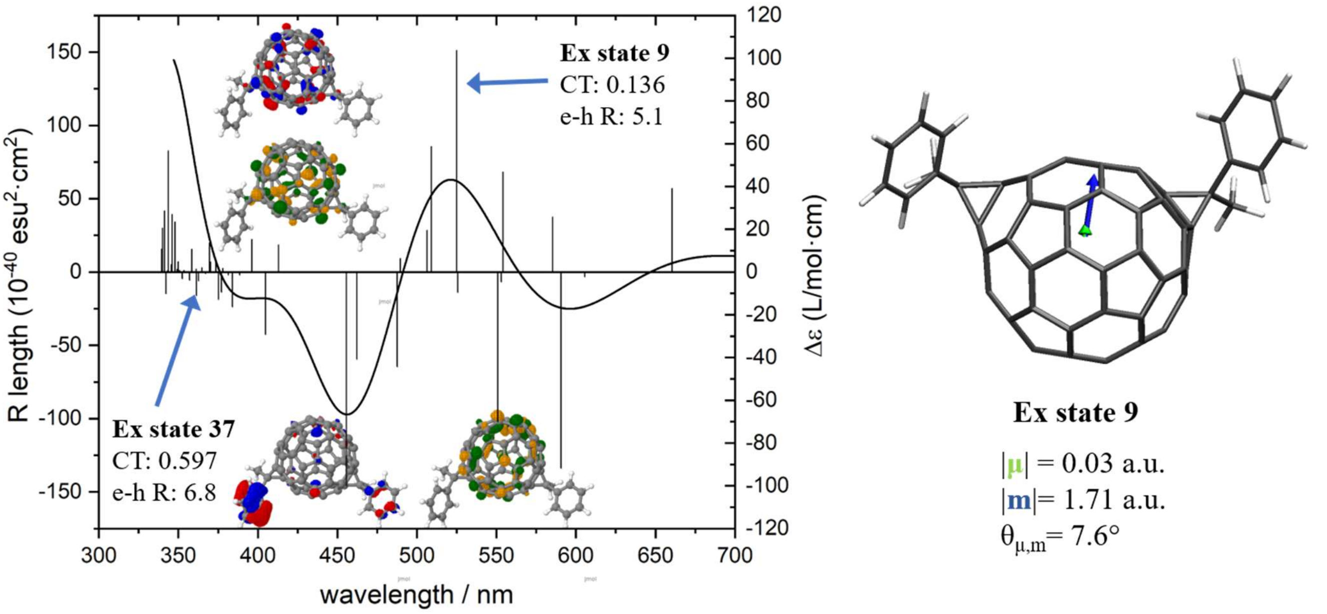

trans-2

($R,R,$ $^{f,s}\underline{A}$)49,59-bis[60]PCBM

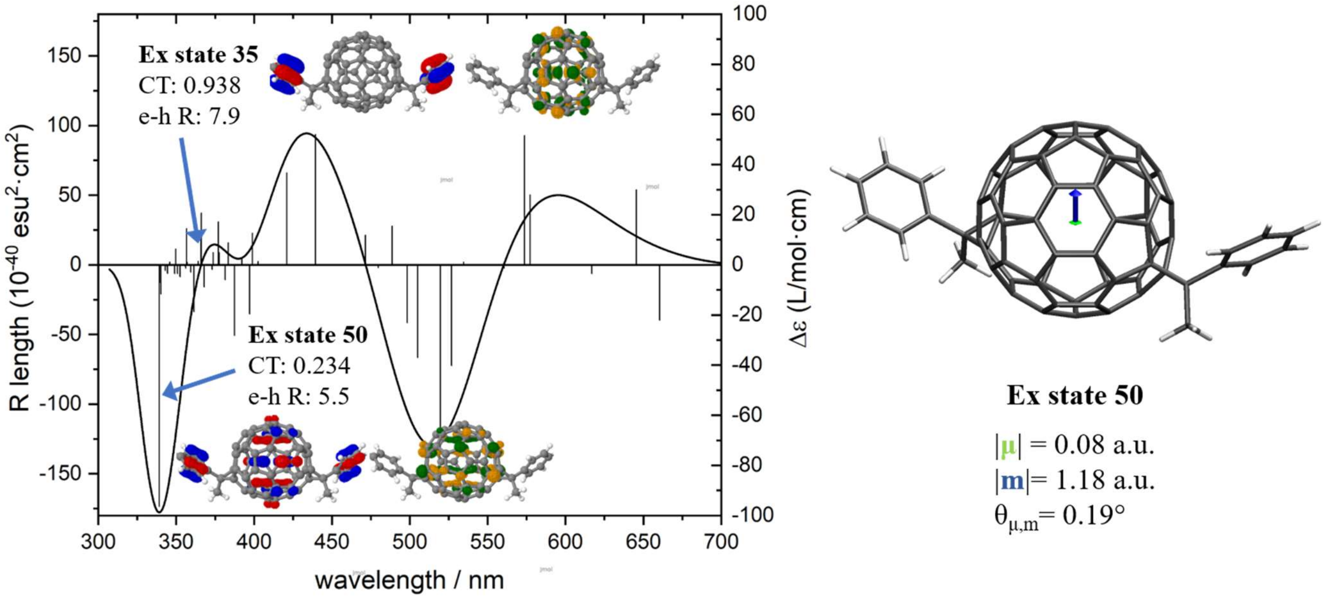

($R,S,$ $^{f,s}\underline{A}$)49,59-bis[60]PCBM

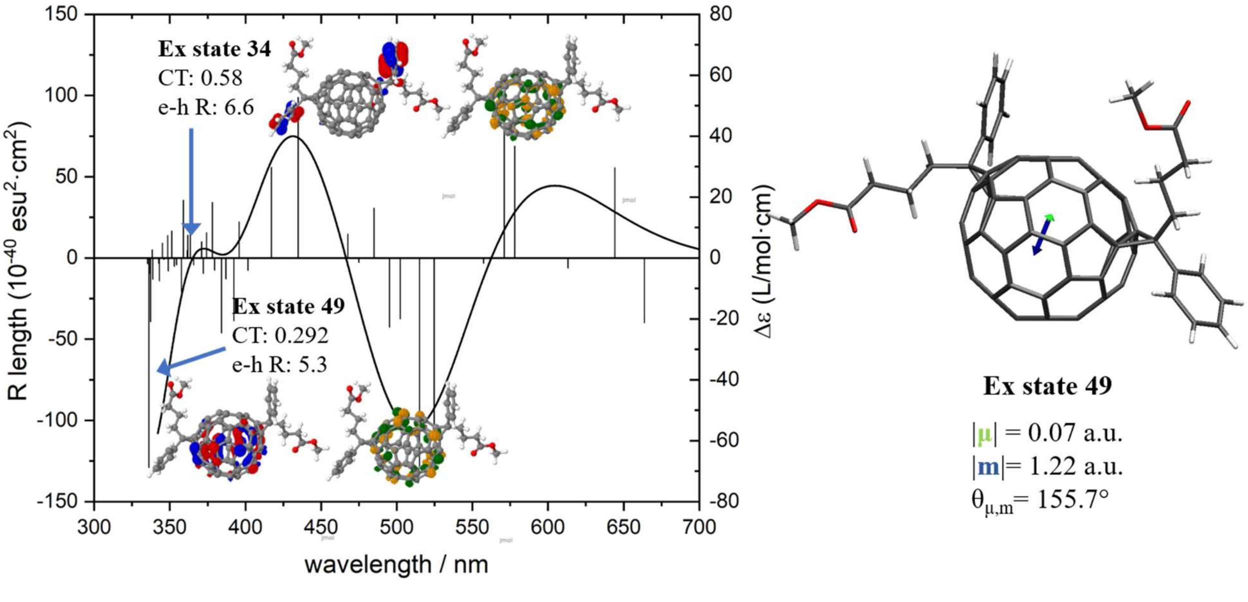

Figure S7 NTOs for the highest CT and the highest R states along with arrow diagrams for every isomer. m, μ and θ indicate the magnetic transition dipole, the electric transition dipole and the angle between them, respectively. The insets show the excitation described as hole-electron pair. Holes are displayed in red/blue and electrons are displayed in yellow/green.

Excited States analysis discussion

In order to establish a connection between the excited states and chiroptical properties, we developed our analysis based on the calculations performed using the Natural Transition Orbital (NTO) formalism as implemented in the software TheoDore.[7] The NTO methodology describes the electronic excitations in terms of electron-hole pairs (excitons), offering a localised picture between the ground state and the excited state of interest. As a consequence of this theoretical construction, many useful quantities can be defined, which provide a more detailed physical insight into the nature of the electronic excitation. Thus, quantities such as natural populations (NP), charge transfer numbers (CT) and exciton size ($R_{e\text{-}h}$) provide a useful framework to analyse the characteristics of the different electronic transition dipole moments.

In general terms, we observe a good agreement between our computed (UV-Vis and CD) and measured spectra at the B3LYP level employing a 6-31G+ basis set. In more detail, one can observe a clear trend excited states depicting a high absorbance intensity at low wavelengths ranges ($\lambda < 450$ nm). In the middle and low wavelength regions ($\lambda > 450$ nm) a clear separation between middle intensity peak moieties such as *cis-2* (*S,S*)3,15-bis[60]PCBM and *e* (*anti,S*)16,17-bis[60]PCBM is contrasted with lack of absorbing states as observed for instance in *cis-3* (*S,R,* ^{f,s}*C*)13,14-bis[60]PCBM and *cis-3* (*S,S,*^{f,s}*C*)13,14-bis[60]PCBM. This clearly indicates that the substitution pattern has an impact on the distribution of electronic excitations and provides a way to effectively tune the magnitude and direction of the electronic transition dipole moment.

This can be further observed as a part of the NTO analysis performed, where we have divided the studied moiety into three different fragments, namely, the bulky C_{60} molecule and the two addends correspondingly. This partition of the moieties allows us to perform a sectorized analysis based on an organic π-conjugated core with a homogeneously distributed electronic density whose electronic properties are distorted by the spatial location of two functional groups. This effect can be quantified by R_{eh} and the CT number. The CT number indicates the amount of charge effectively moved within the cage. As stated in the main text, we observe that strong CT states are related to translational movement of charge.

For states with high rotatory strength, the charge displacement during the excitation occurs within the C_{60} moiety. This can be observed in the excited states 24 ((*S,S*)3,15), 2 ((*S,R,*^{f,s}*C*)13,14), 1 ((*S,S,*^{f,s}*C*)13,14), 25 (*anti,S*)16,17), 29 ((*S,S*)32,33), 8 ((*S,S,*^{f,s}*C*)34,35), 9 ((*R,R,*^{f,s}*C*)34,35), 9 ((*S,R,*^{f,s}*C*)34,35), 50 ((*R,R,*^{f,s}*A*)49,59) and 49 ((*R,S,*^{f,s}*A*)49,59) (**Figure S7**). The local nature of the transition is ascribed to small root mean square R_{eh} numbers that for the studied molecules range from 5.0 to 5.4. This highlights that a major part of the electronic density remains within the C_{60} moiety with a dominant $\pi \rightarrow \pi^*$ character of the associated excitation. This can be observed in the corresponding molecular orbital diagrams displayed along the CD spectra.

On the contrary, we find excited states in which the electron is localised at the bulk and then transferred to the peripheral addends as displayed in 41 ((*S,S*)3,15), 49 ((*S,R,*^{f,s}*C*)13,14), 50 ((*S,S,*^{f,s}*C*)13,14), 43 (*anti,S*), 46 ((*S,S*)32,33), 38

((*S*,*S*,^{f,s}*C*)34,35), 45 ((*R*,*R*,^{f,s}*C*)34,35), 37 ((*R*,*R*,^{f,s}*C*)34,35), 35 ((*R*,*R*,^{f,s}*A*)49,59) and 34 ((*R*,*S*,^{f,s}*A*)49,59). As a general trend, these states display a big root mean square R_{eh} numbers ranging from 6.6 to 7.1 clearly indicating the non-locality of the intramolecular charge transfer. CT numbers are bigger than in the previous case ranging from 0.42 to 0.65 showing the effective migration of charge within the moiety. These states are generally associated with weak circular dichroism.

As important as the electronic features, the chiroptical properties of every molecular species depends on the behaviour of their magnetic characteristics. These are also impacted by the substituents modifying important quantities such as the electronic density distribution, π-conjugation and aromaticity in the different isomers. To investigate these properties, we have employed the anisotropy of the current (induced) density (ACID) method.[13] Throughout these calculations, we have used the same level of theory as specified in the main text. For completeness of our analysis, we have also computed the anionic C_{60}^{6-} and neutral C_{60} fullerene whose magnetic properties have been already established in literature (globally aromatic for the anionic case whereas for the neutral case non-aromaticity has been reported).[14,15] As a starting point, the ACID scalar field has been computed with an isosurface value of 0.05 (arb. units) for the different molecular complexes and displayed in yellow as shown in **Figure S8**. The external magnetic field is aligned to the z-axis passing through a pseudo-C_2 axis of the isomer studied.

The results of these calculations display the global delocalised nature of the total electronic density over the whole system (cage and substituents too) indicating the π-character of the ground state of the molecules. The interaction between the external magnetic field and the molecules can be visualised by employing the current density and projected onto the ACID isosurface (displayed as red and green arrows in Figure S8). This vectorial quantity reveals areas of aromaticity or anti-aromaticity (clockwise-diamagnetic-aromatic or counterclockwise-paramagnetic-antiaromatic) within the studied moiety which are defined by the induction of global or local currents. This has been especially useful in the prediction and interpretation of magnetic properties of 3-D aromatic molecules as in the case of the psuedospherical systems such as C_{60}.[15,16]

According to literature,[17] only some cationic (+10) and anionic (-6) C_{60} species display global aromaticity while the neutral fullerene is globally non-aromatic with regions of local and semilocal anti-aromaticity. Global aromaticity is allowed through processes of charge-carrier reduction or oxidation that enables electronic configurations that complies with the spherical aromaticity rule $2(N+1)^2$ (where N indicates the total number of electrons).[18]

In our case, we observe that all isomer types are globally non-aromatic resembling the behaviour of the parent (neutral) fullerene. These results complement and agree with previously observed trends for other adducts where it has been shown that a large portion of the C_{60} π-system is preserved.[17] The impact caused by the addition of the substituents to the cage generates a local rearrangement of the bonds with a concomitant

removal of electrons from the conjugated system. This is observed in our calculations where an enlargement of the bond from 1.34 Å (pristine Fullerene) to 1.62 Å (two adduct cases) is reported for all cases. Similarly, an enhancement of the Mulliken charges is reported at the vertices of the two adjacent benzenes implying a localisation of electronic charge. Consequently, the aromaticity of the different isomers is altered and mediated by new accessible sigma bonds associated with this double-bond breaking process caused by the substitutions. In this sense, we observe that different bond type isomers show various areas of local aromaticity and anti-aromaticity. The isomers *e, cis-2, cis-3* and *trans-2* show mainly non-aromatic or locally anti-aromatic (5- and 6-membered) rings on the cage, while *trans-3* and *trans-4* have a predominance of aromatic areas. Similarly, the phenyl rings of the adducts display different behaviours such as weak ring currents (*trans-4* and *trans-2*), aromaticity (*cis-2, cis-3* and *e*), or anti-aromaticity situation (*trans-3*). Thus, the localisation of charge is effectively destroying the observed network of local and semilocal antiaromaticities formed in C_{60} creating small spin densities that interact also with the external magnetic field as observed in other substituted fullerenes.

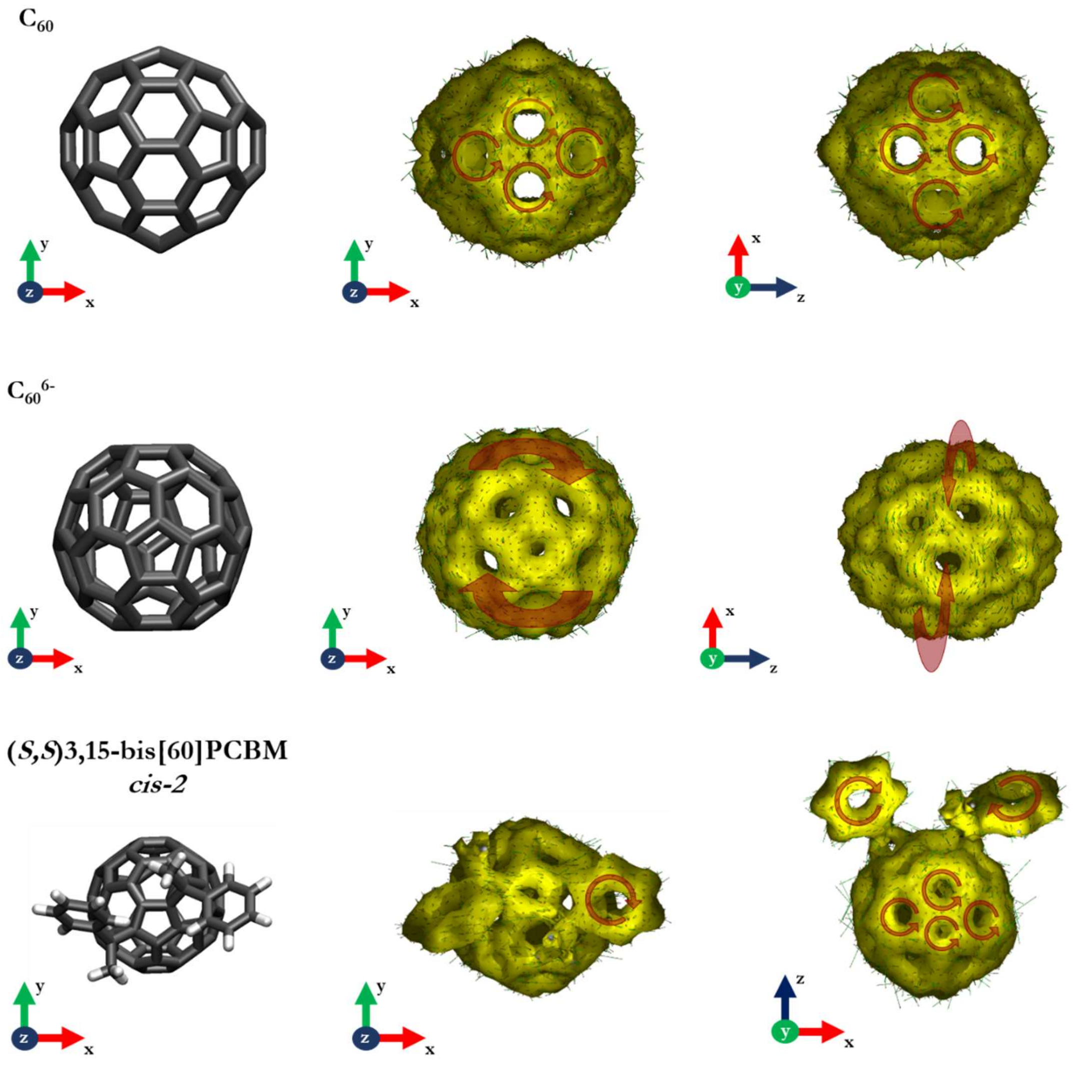

(*S,S,* *^{f,s}C*)13,14-bis[60]PCBM
cis-3

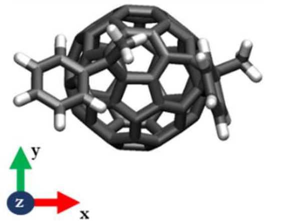 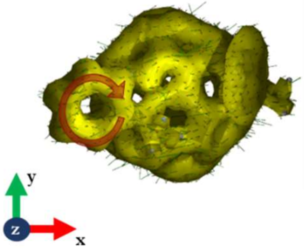 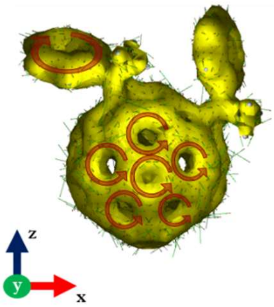

(*anti,R*)16,17-bis[60]PCBM
e

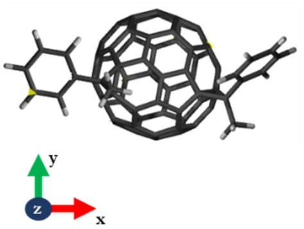 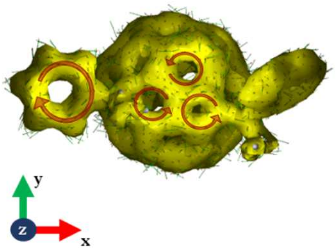 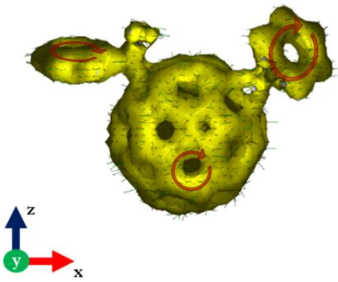

(*S,R,* *^{f,s}C*)49,59-bis[60]PCBM
***trans*-2**

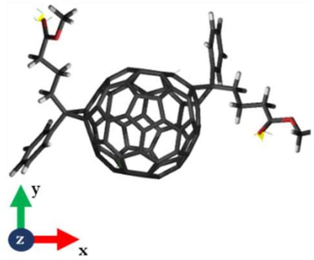 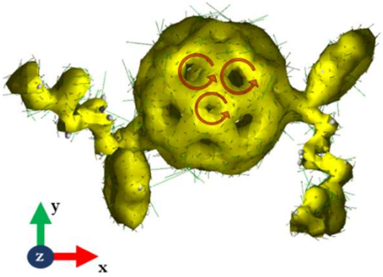 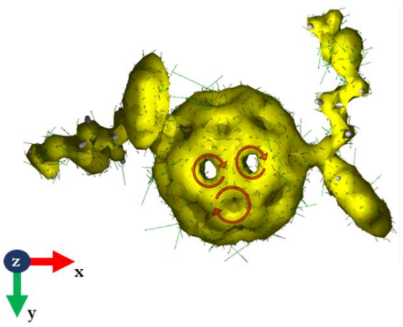

(*R,R,* *^{f,s}C*)34,35-bis[60]PCBM
trans-3

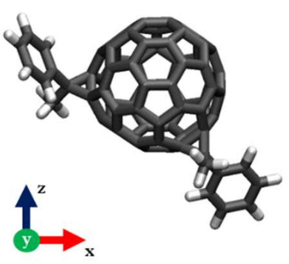 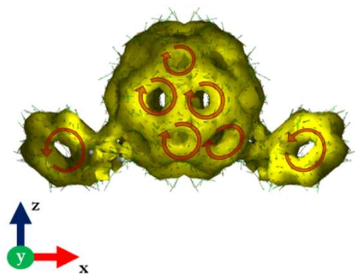 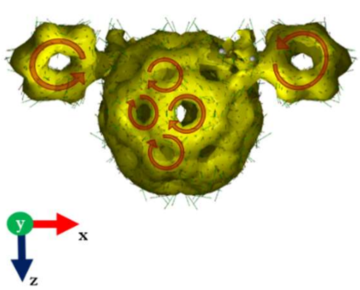

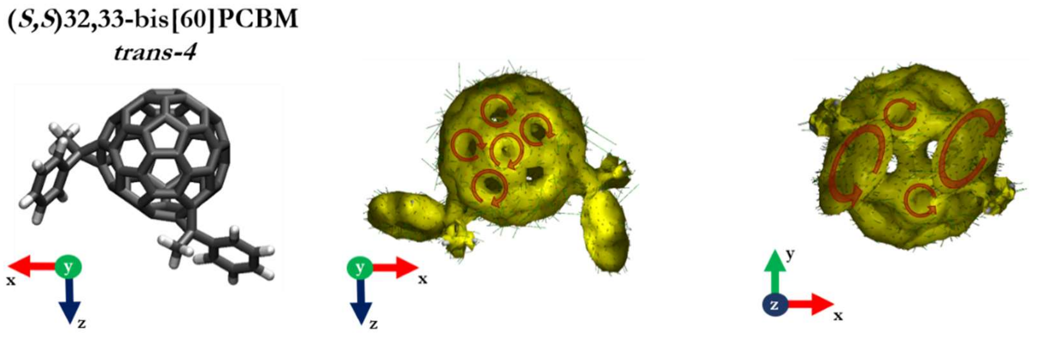

Figure S8 Anisotropy of the induced current density (ACID) plots to visualize electronic delocalization, at an isovalue of 0.05. One isomer per bond-type has been analysed. Large red arrows indicate the direction of the small current density vectors, which show diatropic (clockwise, aromatic) and paratropic (counterclockwise, anti-aromatic) local ring currents.

To summarise, chiroptical activity is the result of several simultaneous factors. Overall, states displaying high rotatory strength intensity must involve a rotational movement of charge (that is a strong magnetic transition dipole) together with a small angle between electric and magnetic transition dipoles. We found that the magnetic properties and the aromaticity of the compound are also changed by the adducts. The adducts change the local electronic density distribution producing the destruction of local antiaromatic networks but facilitating the localisation of charge which interacts also with the magnetic field component of the pulse. Therefore, the chiroptical properties are the result of a combination of local collective aromatic and antiaromatic rings and enhanced localisation of charge which can only be explained by means of performing fully comprehensive theoretical analysis.

Different viewpoints of all the enantiomers

The right image represents the image on the left rotated by 90° around a vertical axis.

(*S*,*S*, ^{f,s}*A*)49,59-bis[60]PCBM

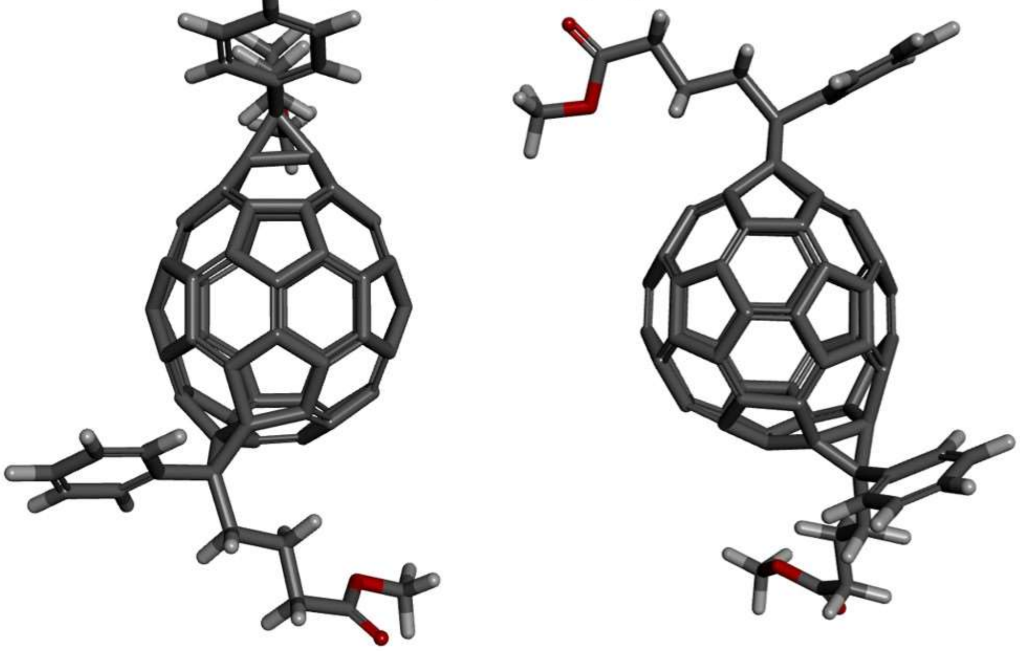

(*R*,*R*, ^{f,s}*C*)49,59-bis[60]PCBM

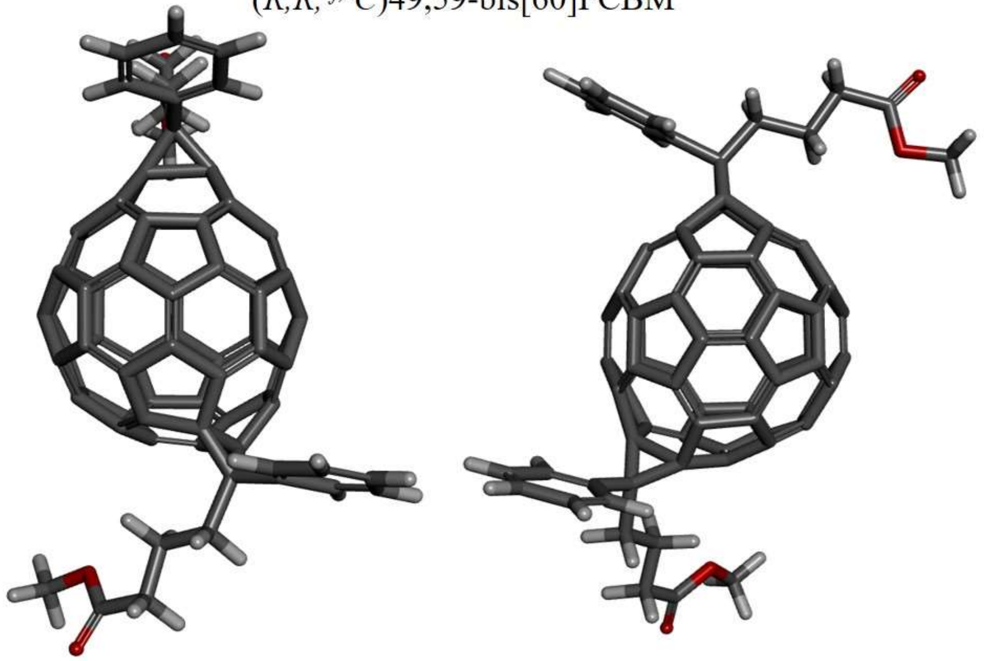

(*R*,*S*, ^{f,s}*A*)49,59-bis[60]PCBM

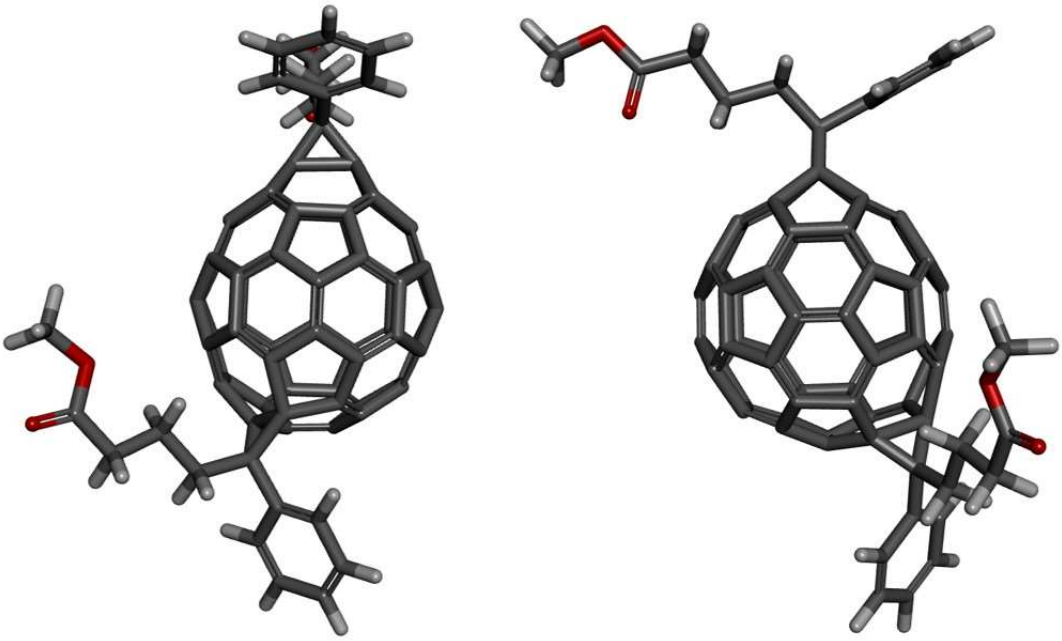

(*S*,*R*, ^{f,s}*C*)49,59-bis[60]PCBM

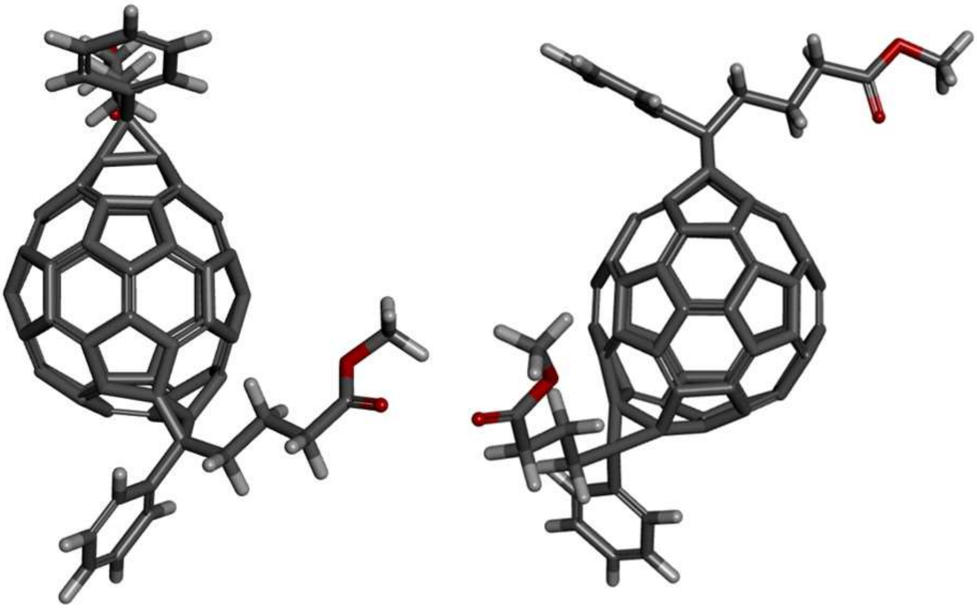

(*S,S,* ^{f,s}*C*)34,35-bis[60]PCBM

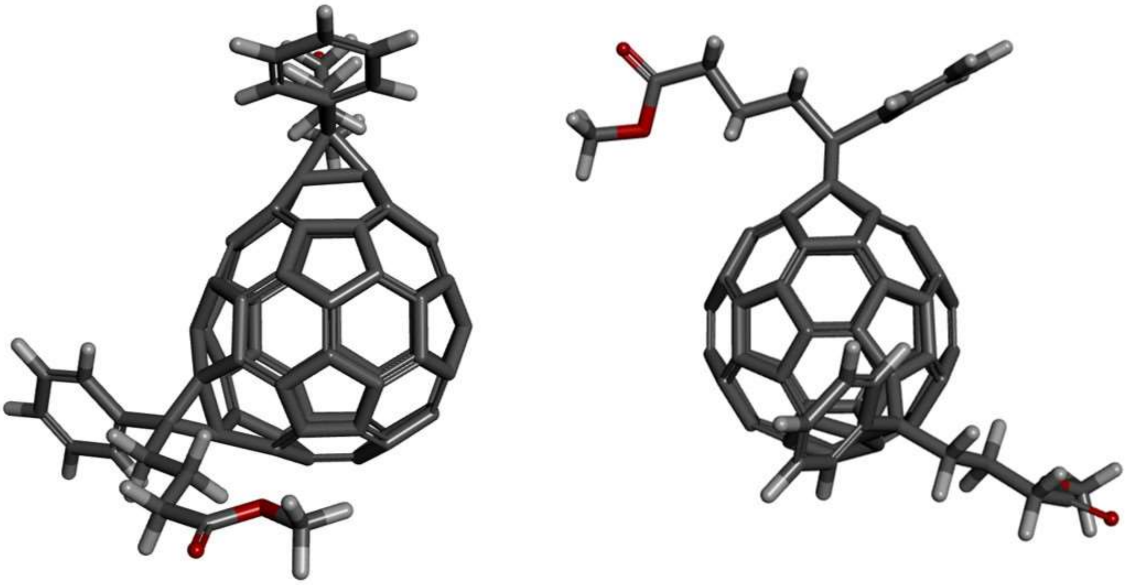

(*R,R,* ^{f,s}*A*)34,35-bis[60]PCBM

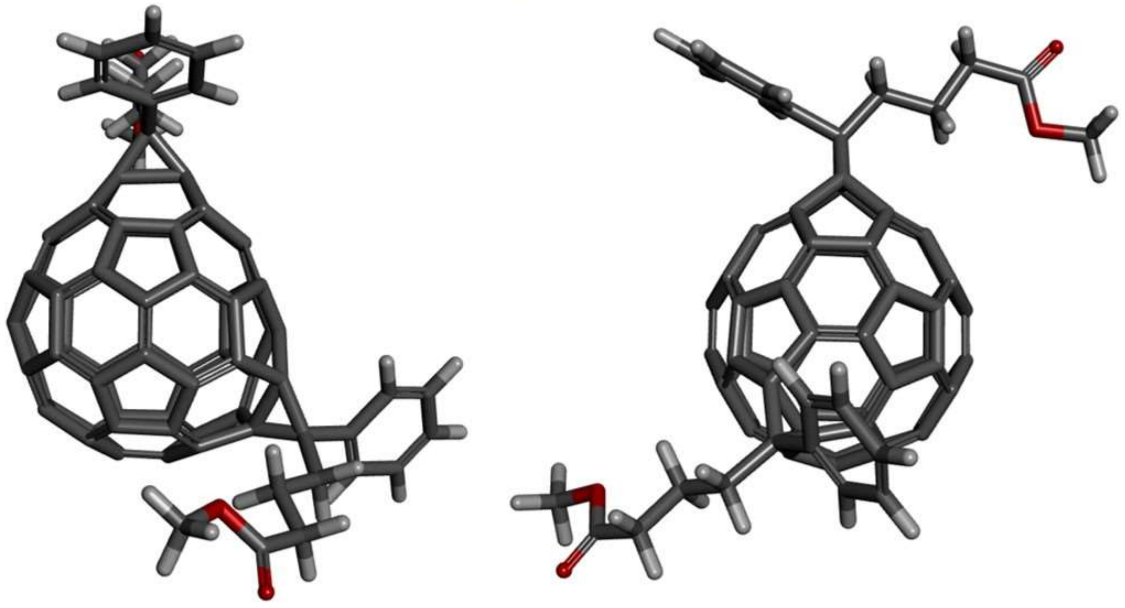

(*S*,*S*, ^{f,s}*C*)49,59-bis[60]PCBM

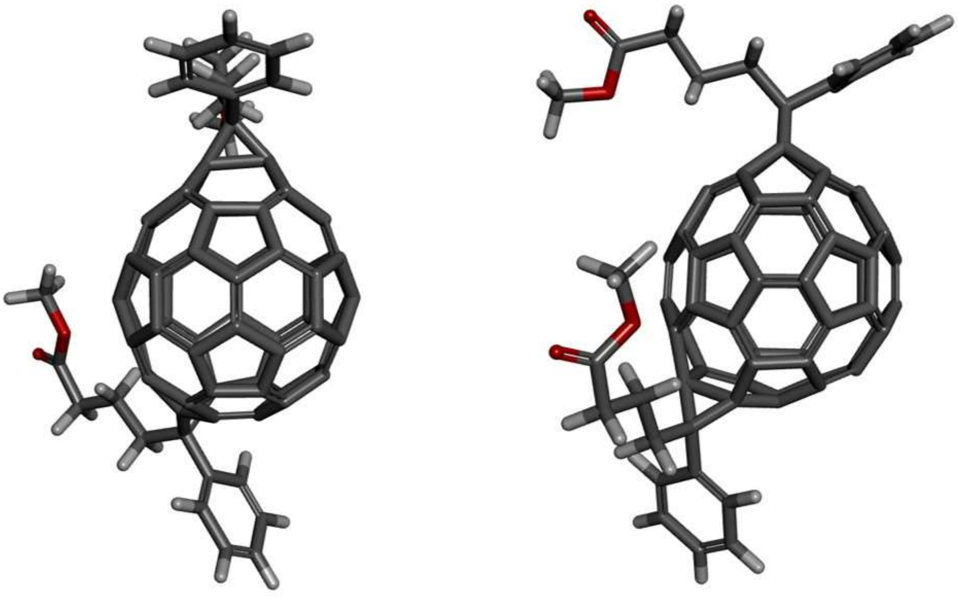

(*R*,*R*, ^{f,s}*A*)49,59-bis[60]PCBM

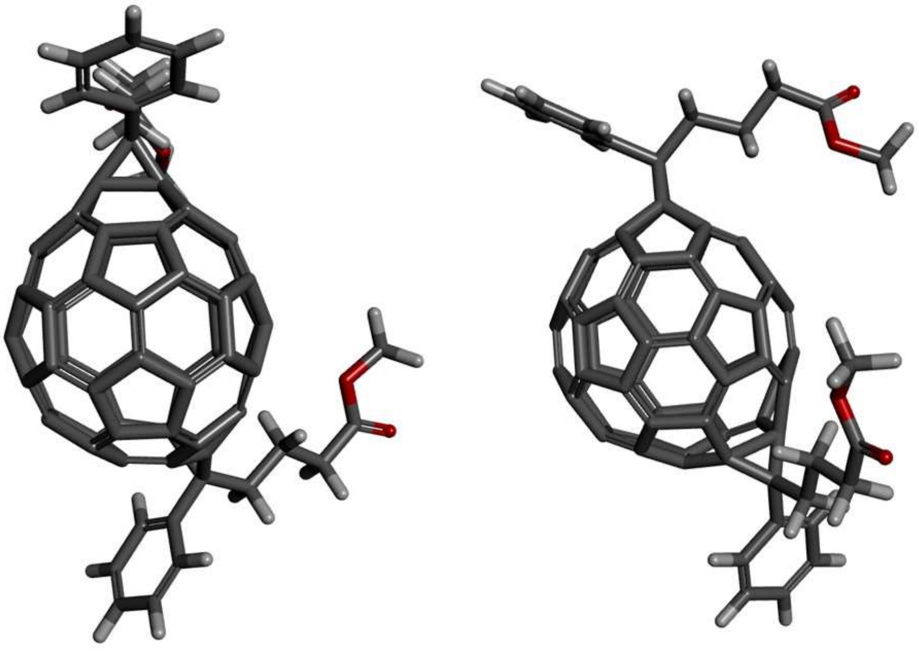

$(R,S,{}^{f,s}A)$34,35-bis[60]PCBM

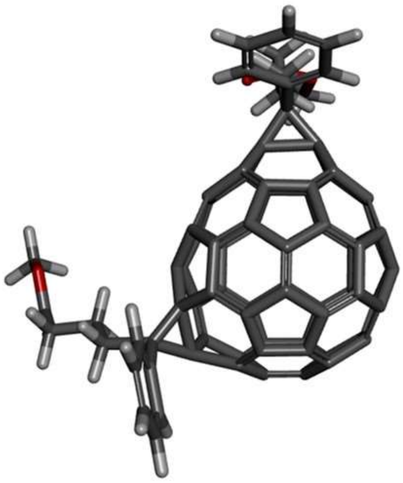

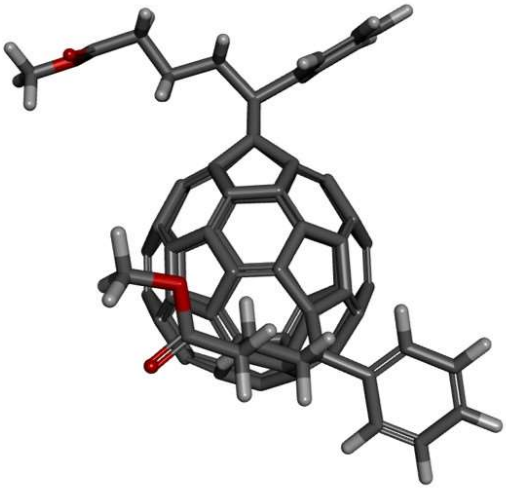

$(S,R,{}^{f,s}C)$34,35-bis[60]PCBM

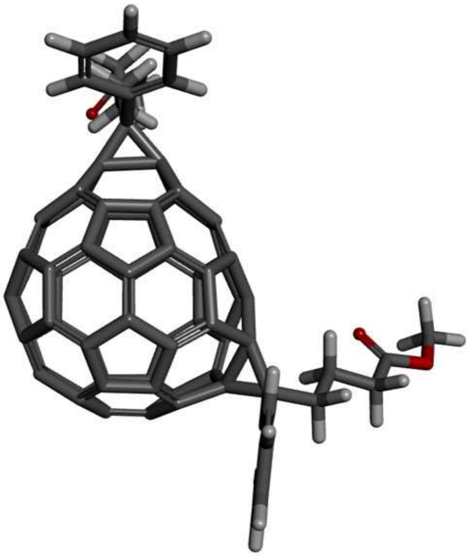

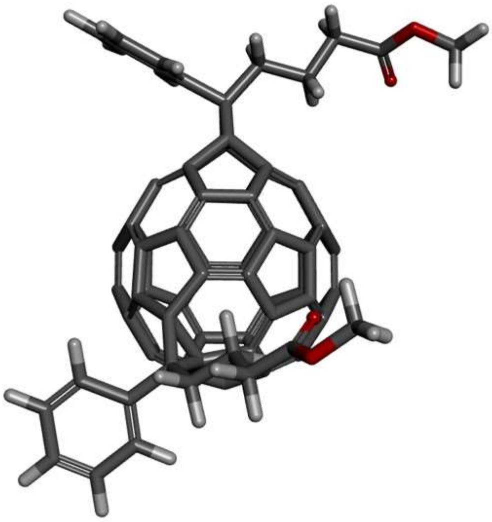

(*S,S*)32,33-bis[60]PCBM

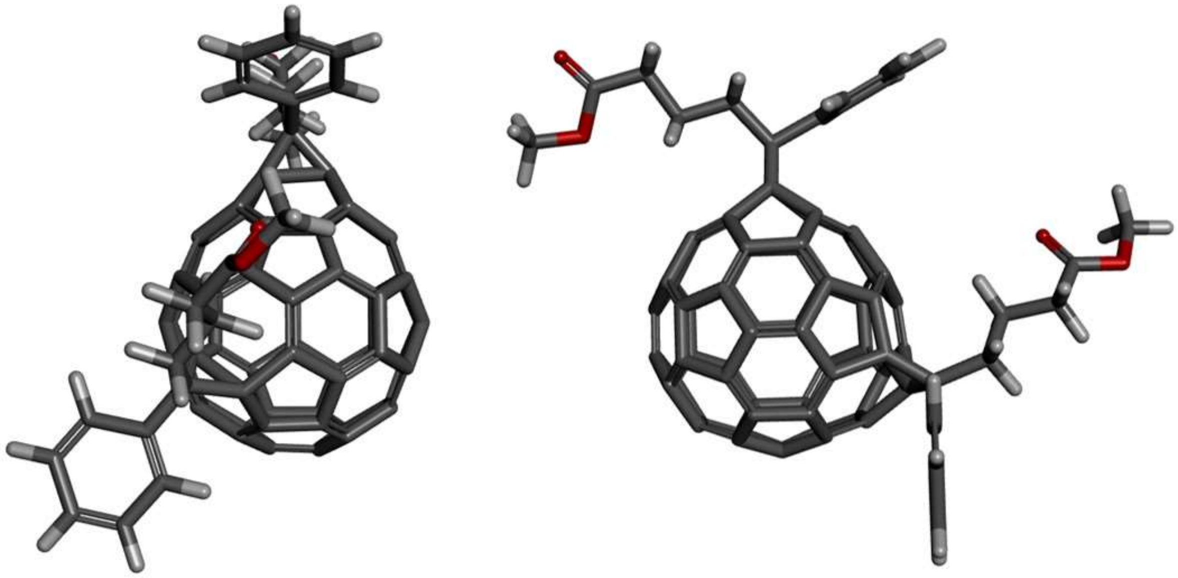

(*R,R*)32,33-bis[60]PCBM

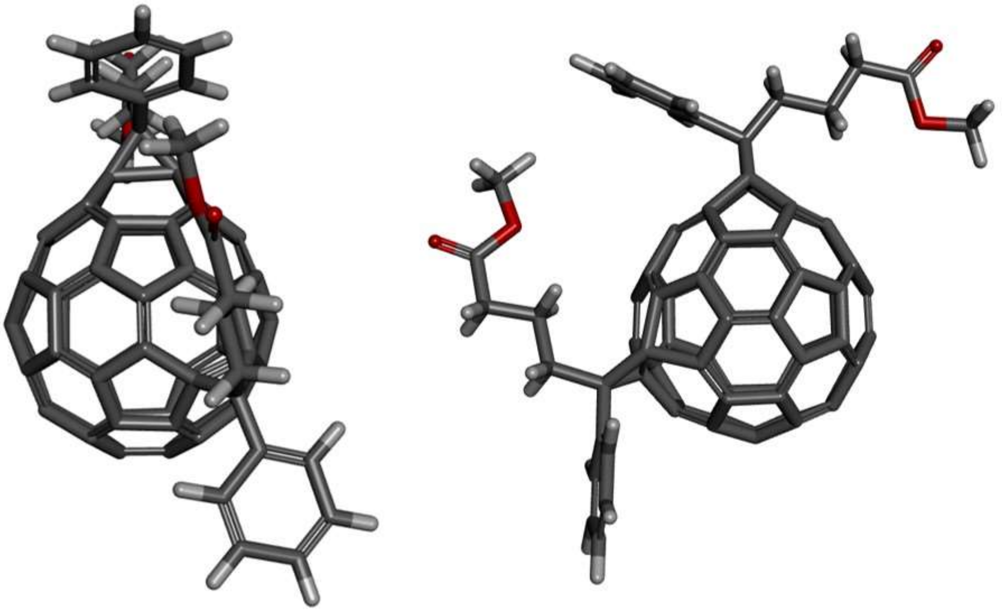

(*S,S,* ^{f,s}*A*)34,35-bis[60]PCBM

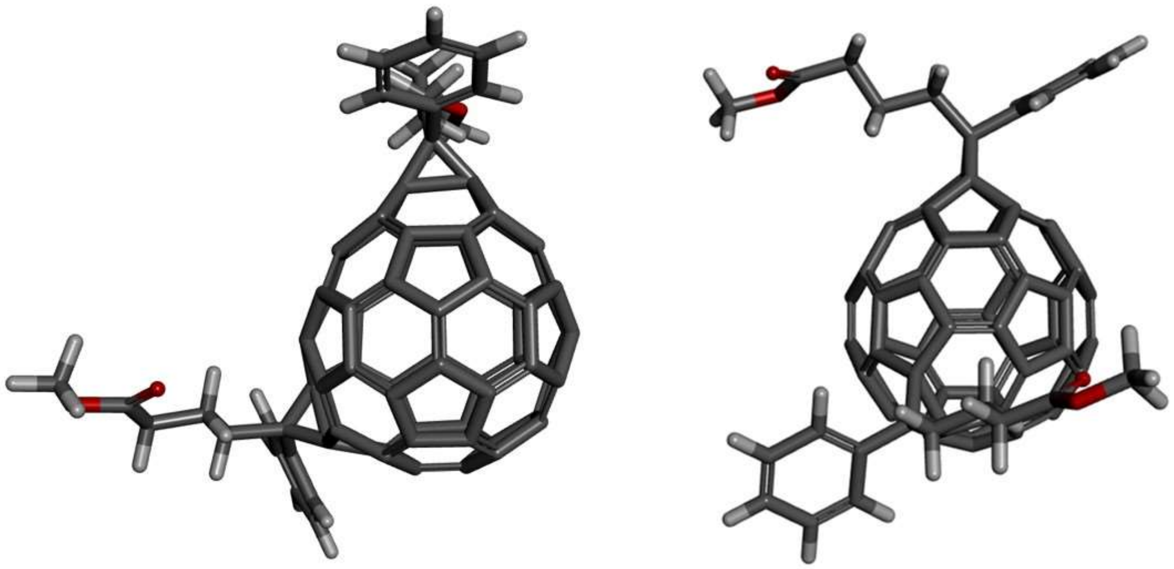

(*R,R,* ^{f,s}*C*)34,35-bis[60]PCBM

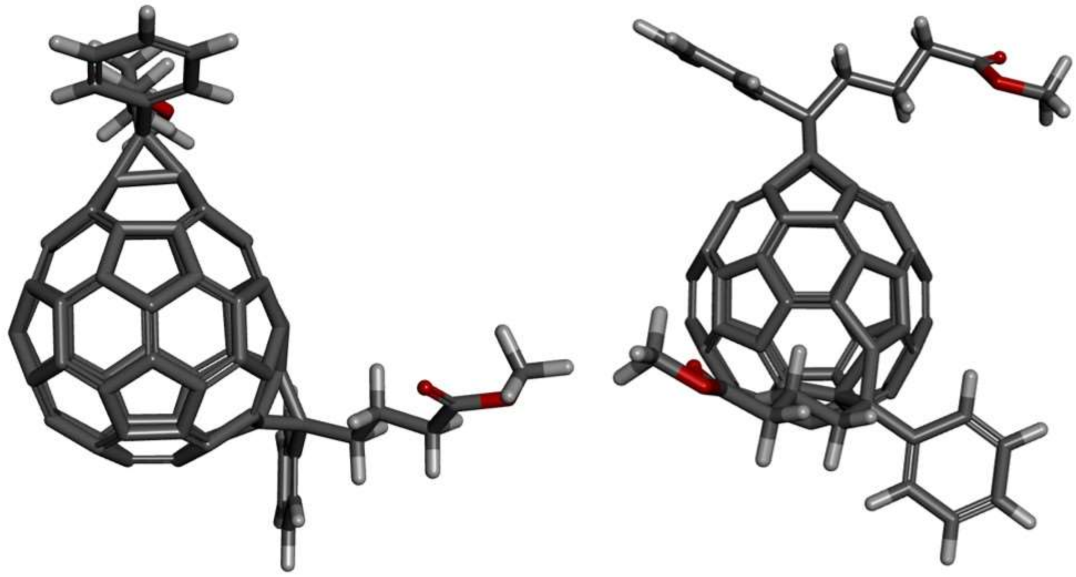

(*syn,S*)16,17-bis[60]PCBM

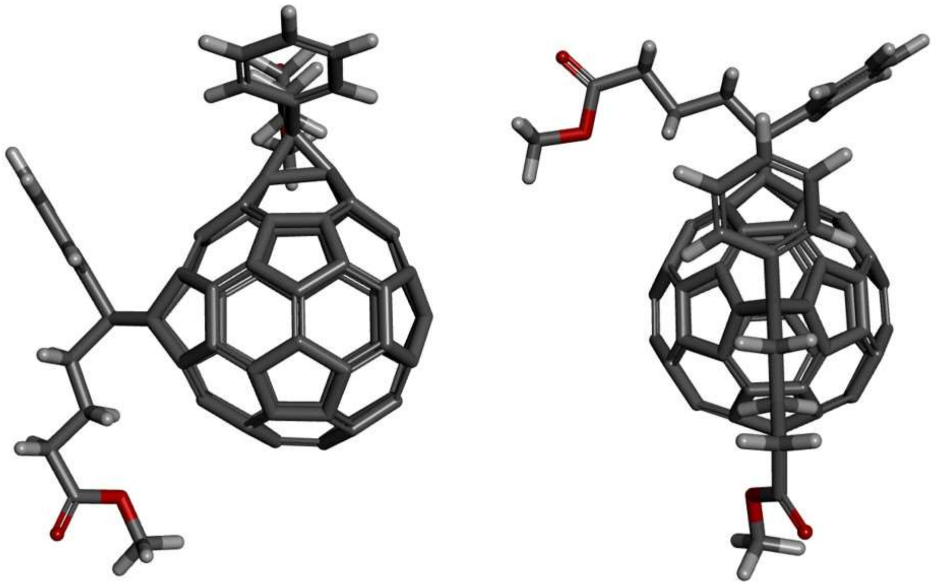

(*syn,R*)16,17-bis[60]PCBM

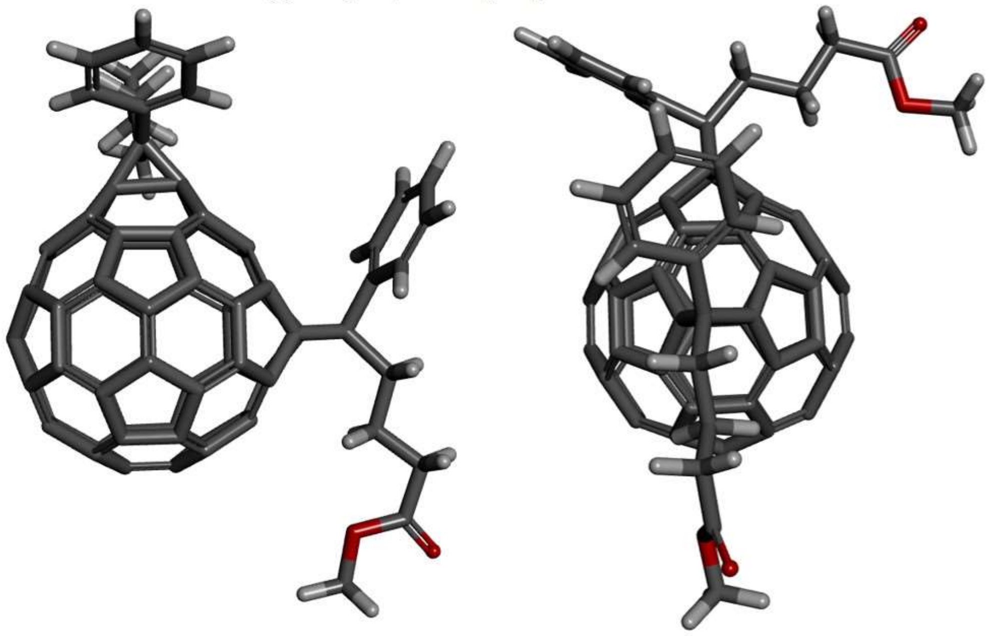

(*S*,*S*, ^{f,s}*C*)13,14-bis[60]PCBM

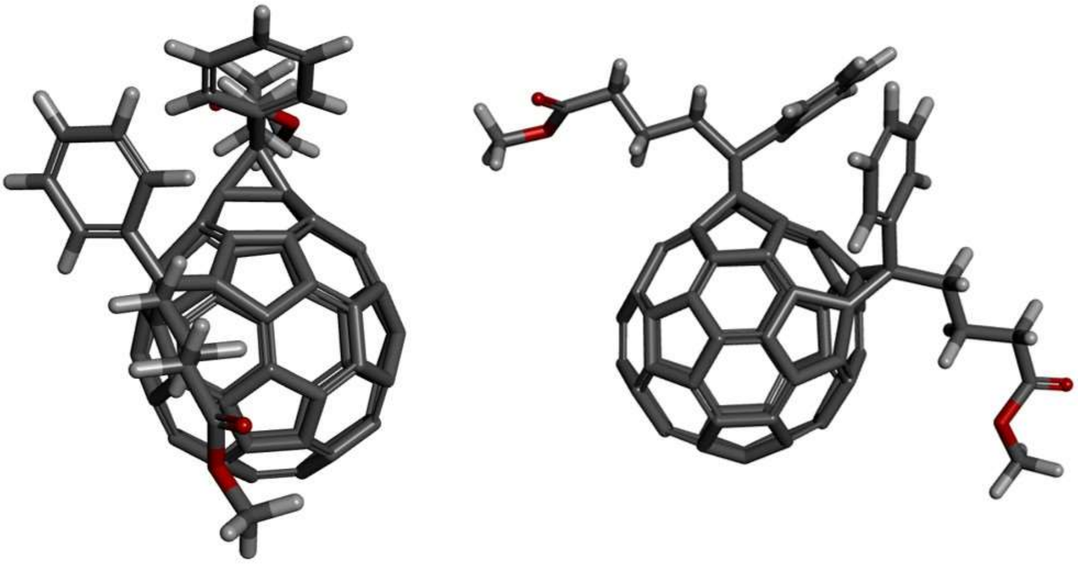

(*R*,*R*, ^{f,s}*A*)13,14-bis[60]PCBM

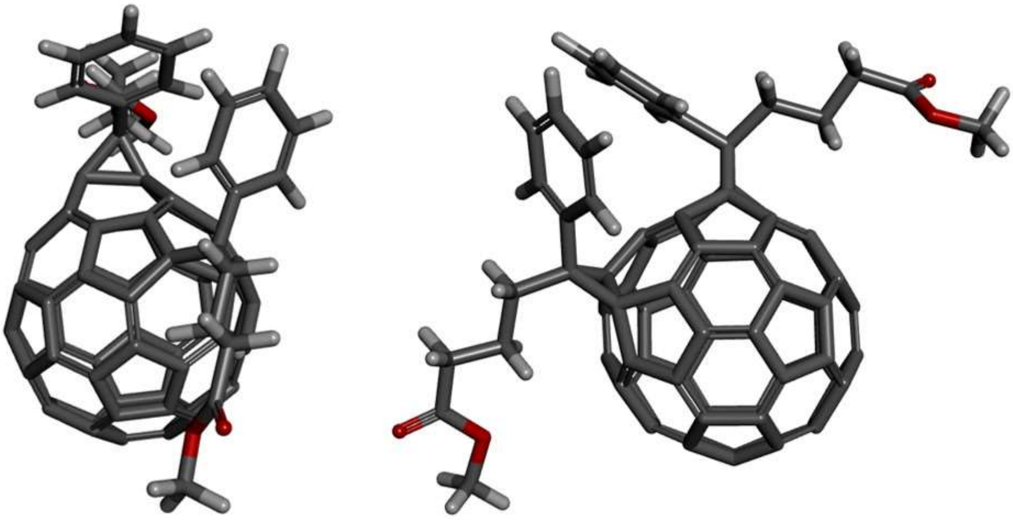

(*anti,S*)16,17-bis[60]PCBM

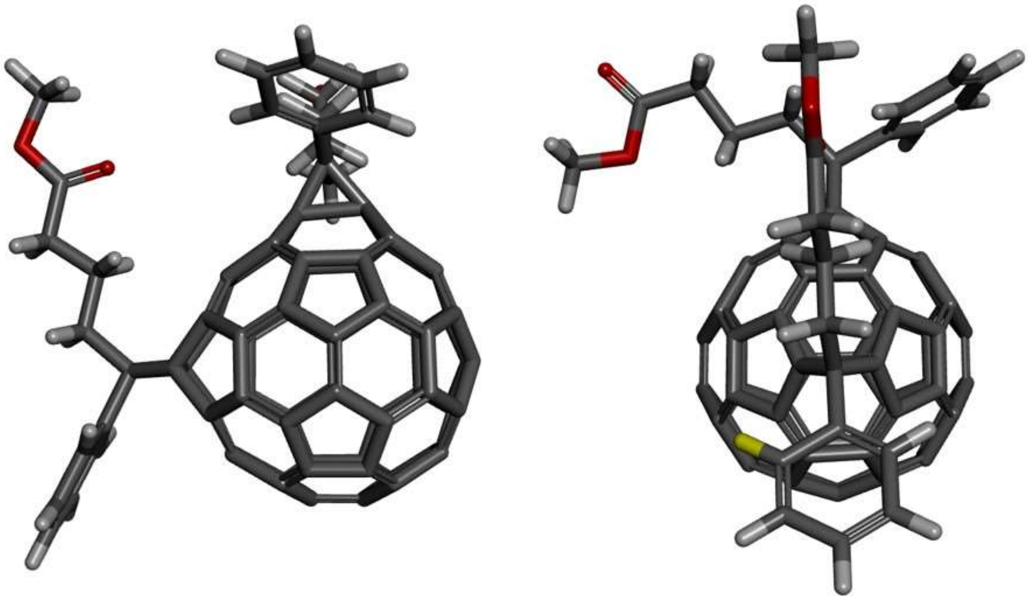

(*anti,R*)16,17-bis[60]PCBM

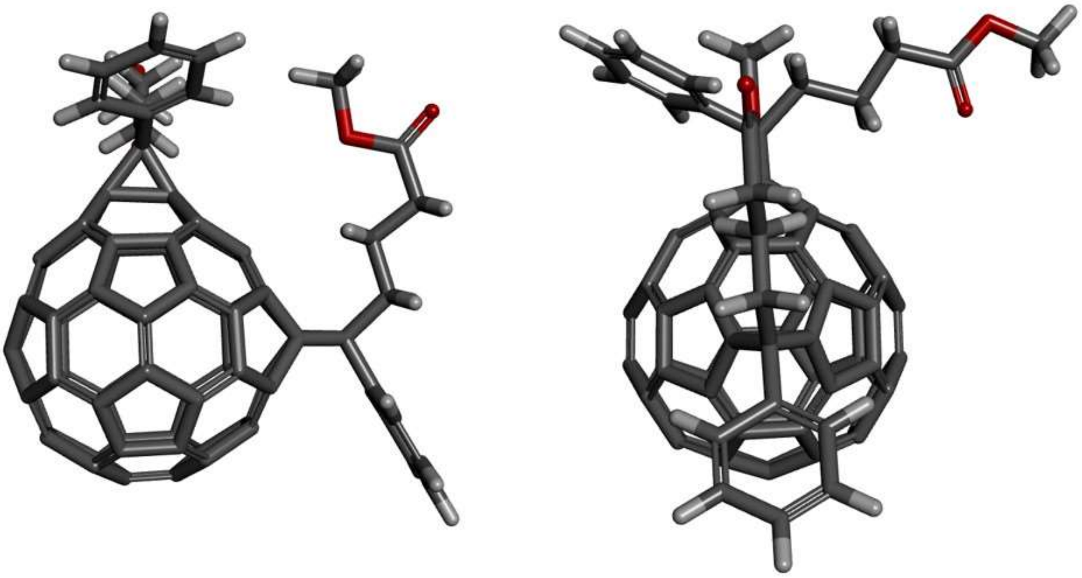

(*S*,*S*, ^{f,s}*A*) 13,14-bis[60]PCBM

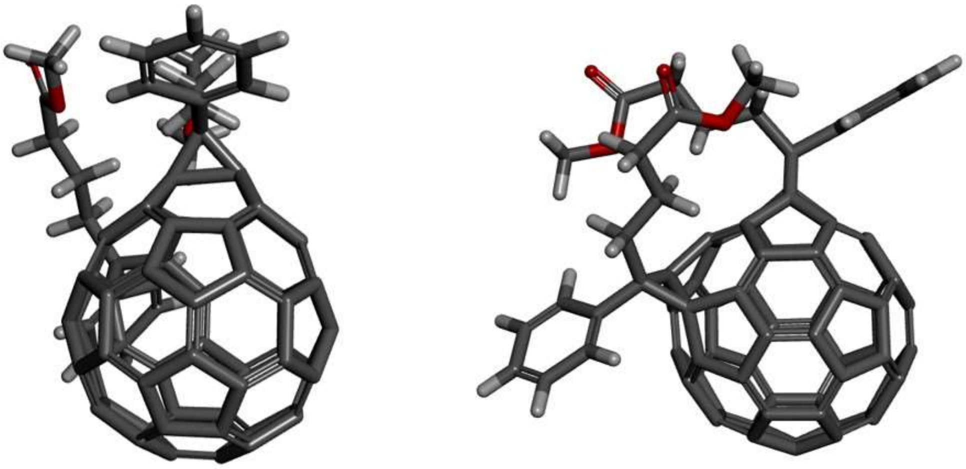

(*R*,*R*, ^{f,s}*C*) 13,14-bis[60]PCBM

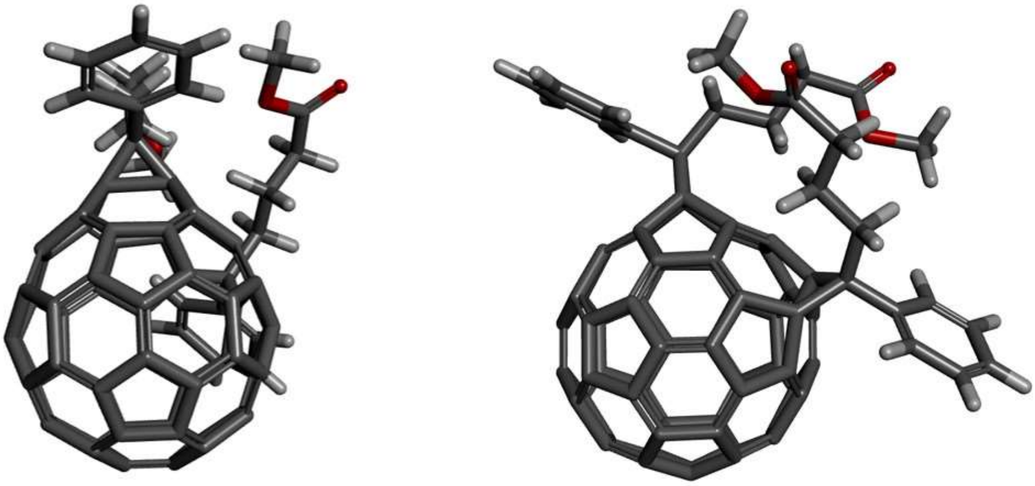

(*R*,*S*, ^{f,s}*C*)13,14-bis[60]PCBM

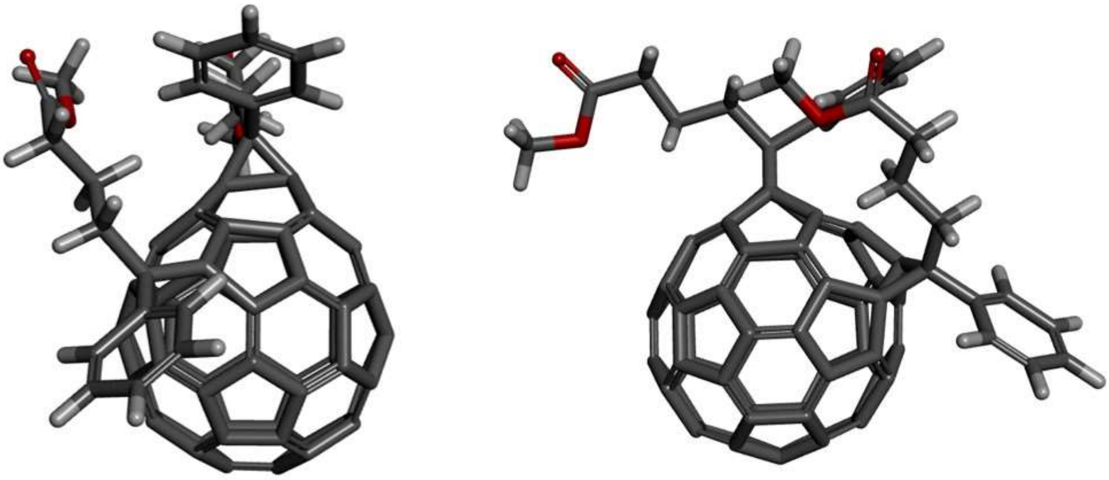

(*S*,*R*, ^{f,s}*A*)13,14-bis[60]PCBM

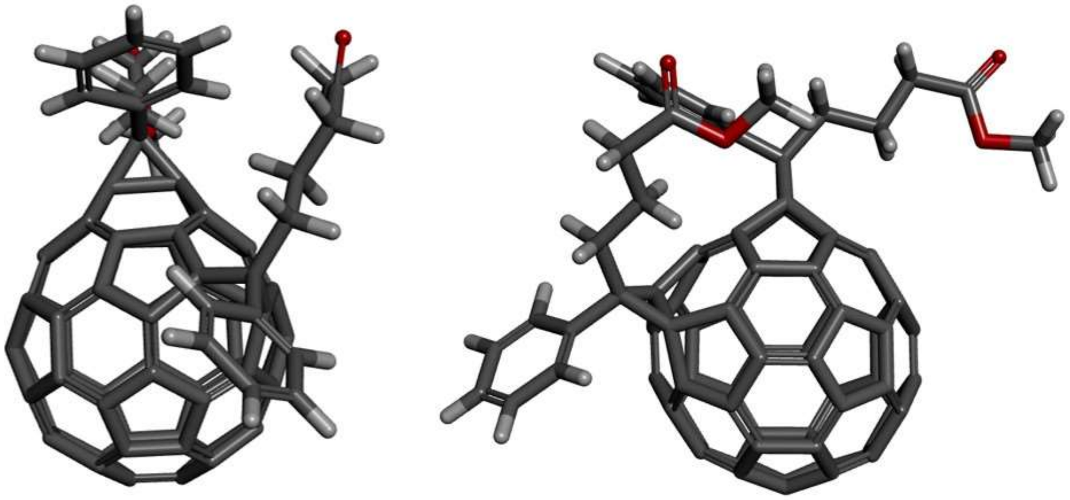

(*S,S*)3,15-bis[60]PCBM

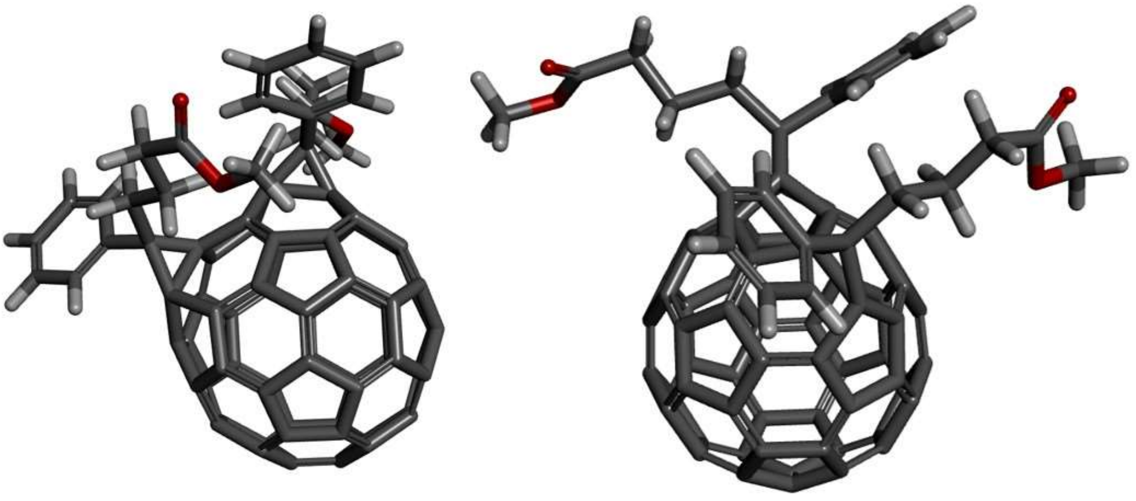

(*R,R*)3,15-bis[60]PCBM

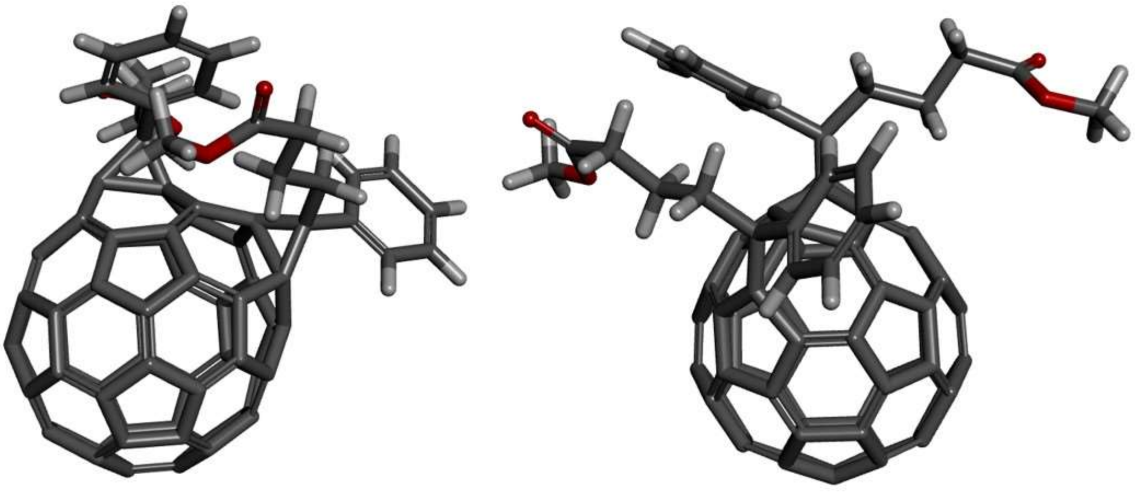

Thin film characterisation

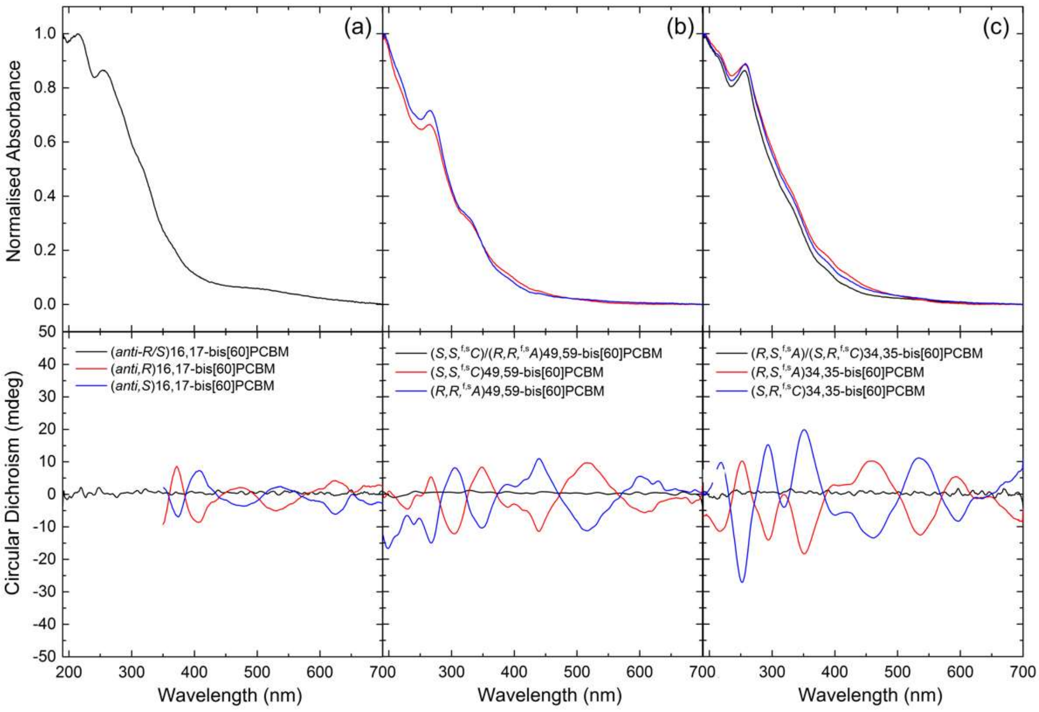

Figure S9 Absorption and CD spectra for thin films (thickness ~ 40 nm) of three pairs of enantiomers (red/blue) and their racemic counterparts (black). The naming convention can be found in Table S1.

All isomers present similar absorption profiles, with weak, broad peaks in the visible and more intense, distinct peaks in the UV part of the spectrum. All isomers display absorption bands around 260, 343 and 445 nm, which is consistent with previous reports on thin films of C_{60}. The CD spectra of the enantiopure isomers are comparable to their solution spectra (**Figure S2**). They exhibit an equal-and-opposite chiroptical response, with peaks corresponding to the various excited states.

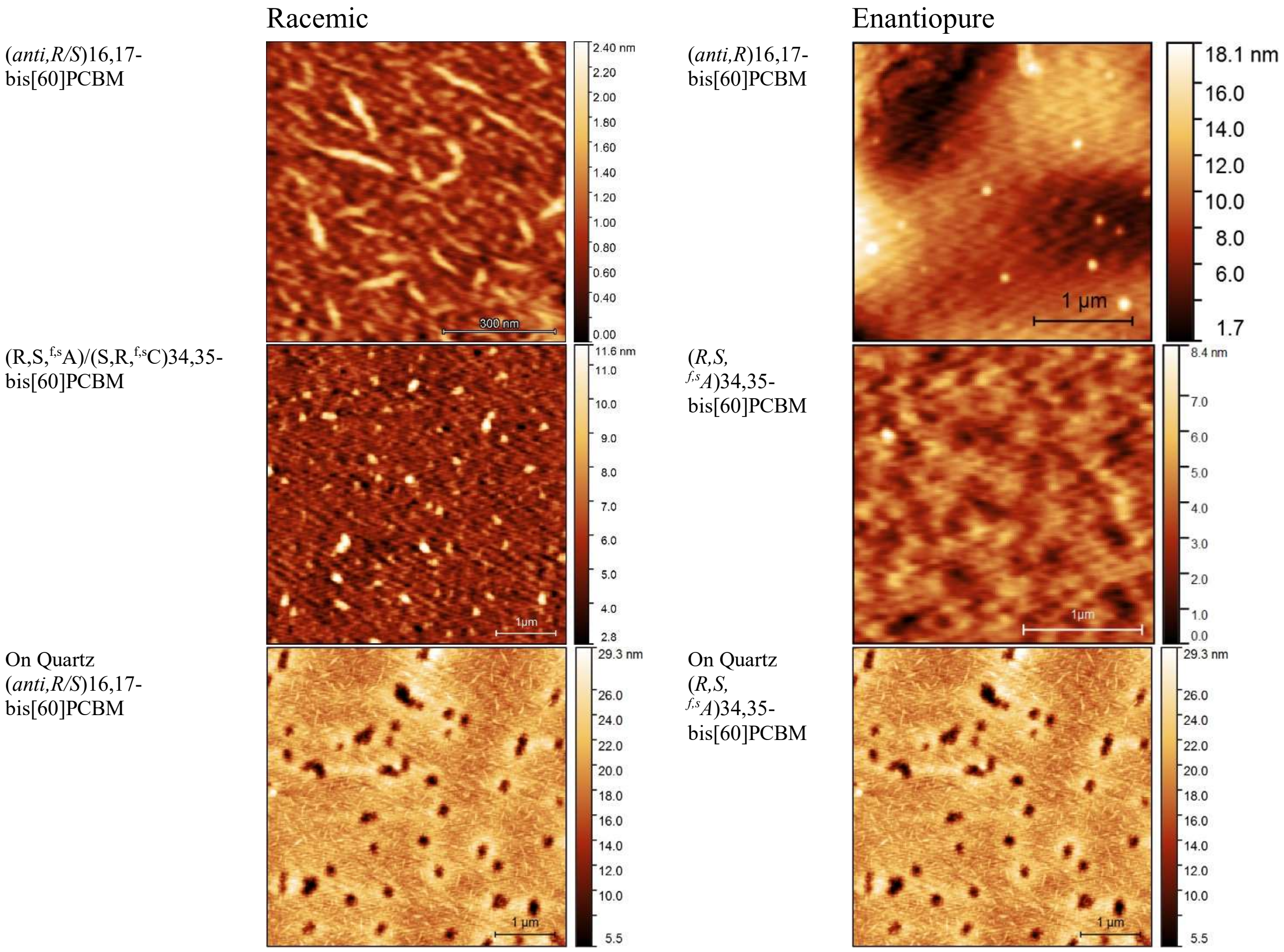

Figure S10 Atomic force micrographs of thin films (thickness ~ 40 nm) of two representative enantiomeric and two racemic fullerene cages. The first two rows are measured on OFET substrates and the final row on quartz. The naming convention can be found in **Table S1**. The scale bars for each image are indicated.

AFM investigations indicate that all enantiomers form relatively smooth (root-mean-square roughness, $r_q \sim 0.5 – 1$ nm), featureless films. On the other hand, micrographs of the racemic isomers ((*R,S,* ^{f,s}*A*)/(*S,R,* ^{f,s}*C*)34,35-bis[60]PCBM and (*anti,R/S*)16,17-bis[60]PCBM) reveal the formation of clusters, 100s of nms across, with the films exhibiting increased roughness ($r_q \sim 2$ nm). A comparison of AFM scans of the thin films on quartz and in complete devices emphasise the importance of the HMDS layer in improving the wettability of the fullerene solution; whilst the samples on quartz are littered by pin holes ($r_q \sim 10$ nm)., these are largely absent on the transistor substrates.

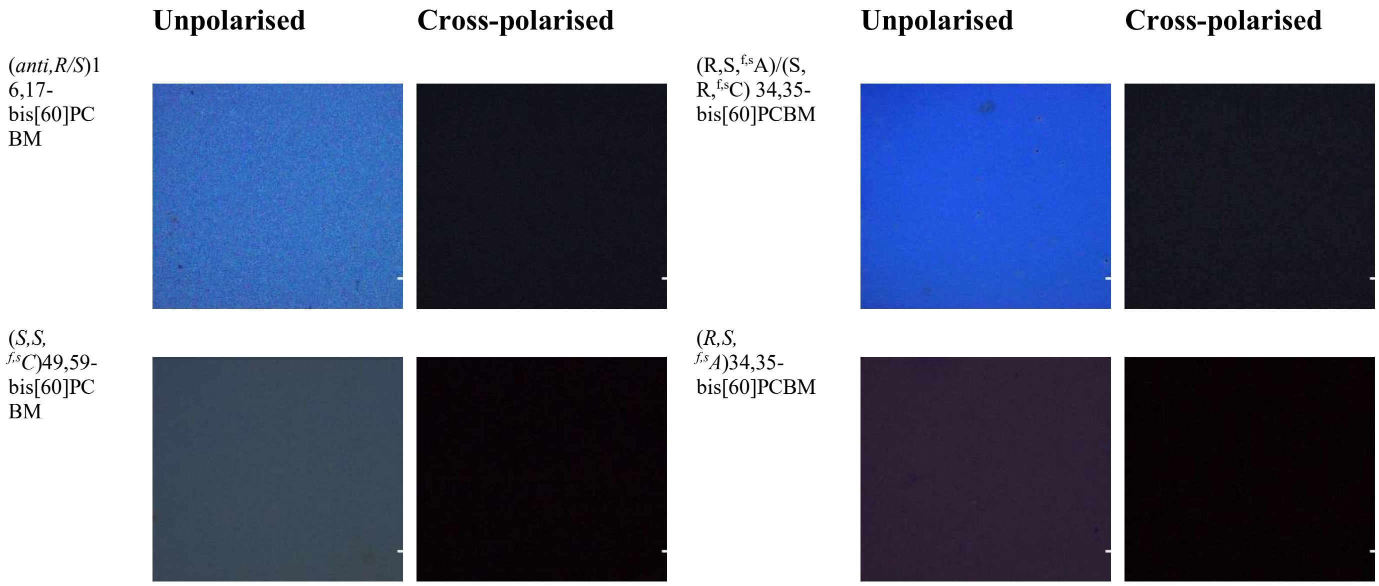

Figure S11 Optical micrographs (unpolarised and polarised) of thin films (thickness ~ 40 nm) of representative enantiomeric and racemic fullerenes. The naming convention can be found in **Table S1**. The scale bars for each image are indicated.

The unpolarised and cross-polarised microscope images of racemic (top row) and enantiopure (bottom row) thin films confirm the absence of any aligned crystalline domains. In particular, the lack of any colour when rotating the crossed polarisers indicate no linear birefringence, which suggests that the CP-photo response observed in devices is due to an intrinsic property of the systems considered, and not the formation of a chiral supramolecular structure.

Device Measurements

PhotoFET Testing Setup

View from above

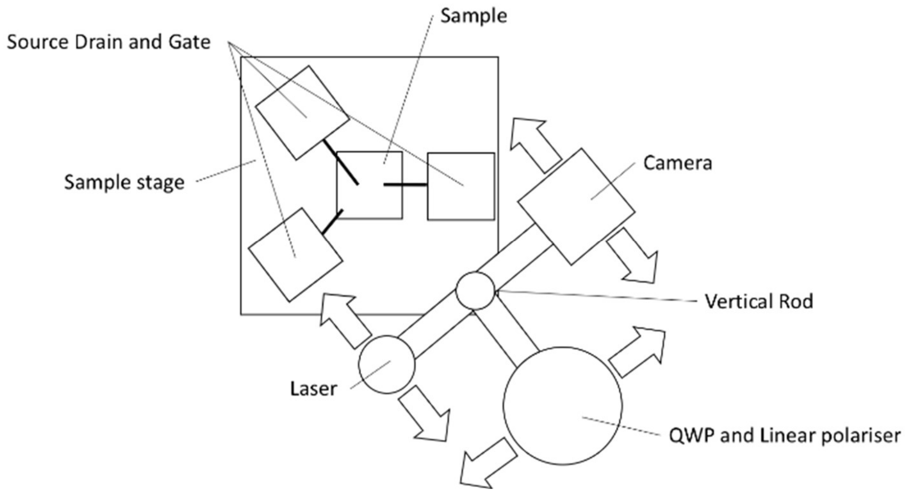

Side view

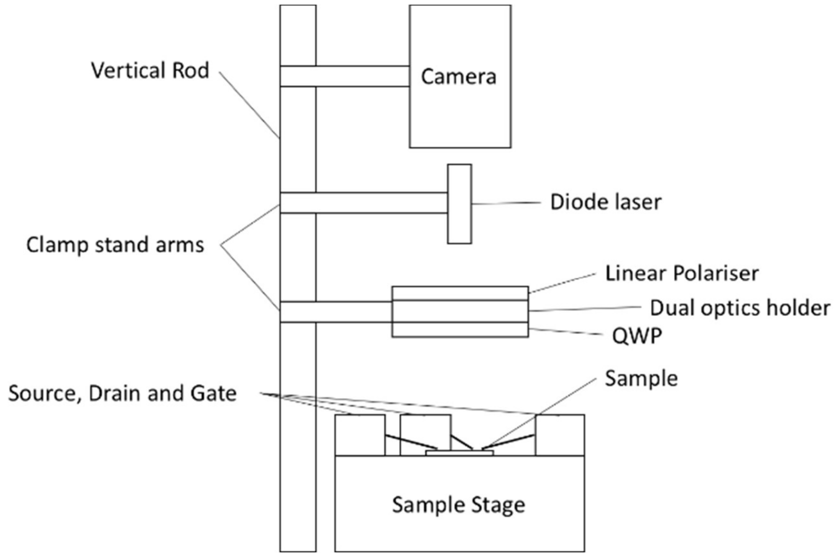

Figure S12 Diagram of the experimental setup for CP photoFET measurements

Additional (*anti,R*)16,17-bis[60]PCBM Measurements

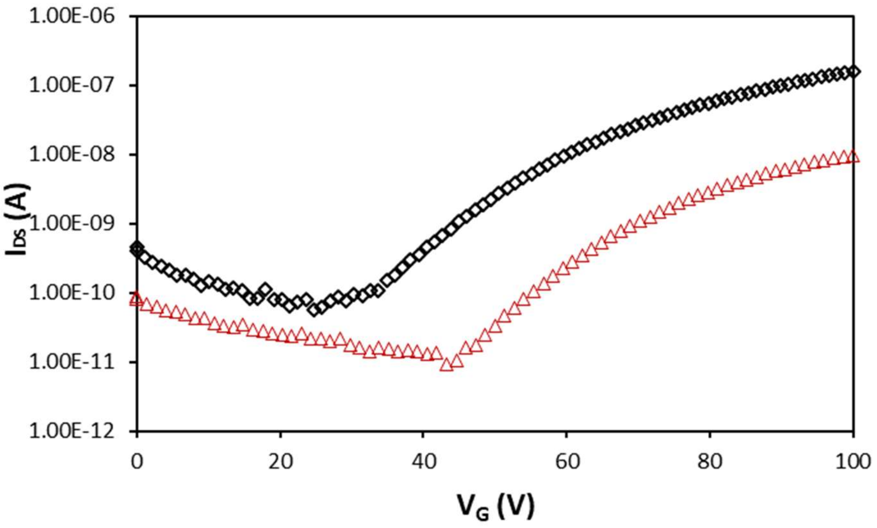

Figure S13 The saturation regimes (black) V_{DS}=100V and linear regime (red) V_{DS}=20V of transfer characteristics under dark.

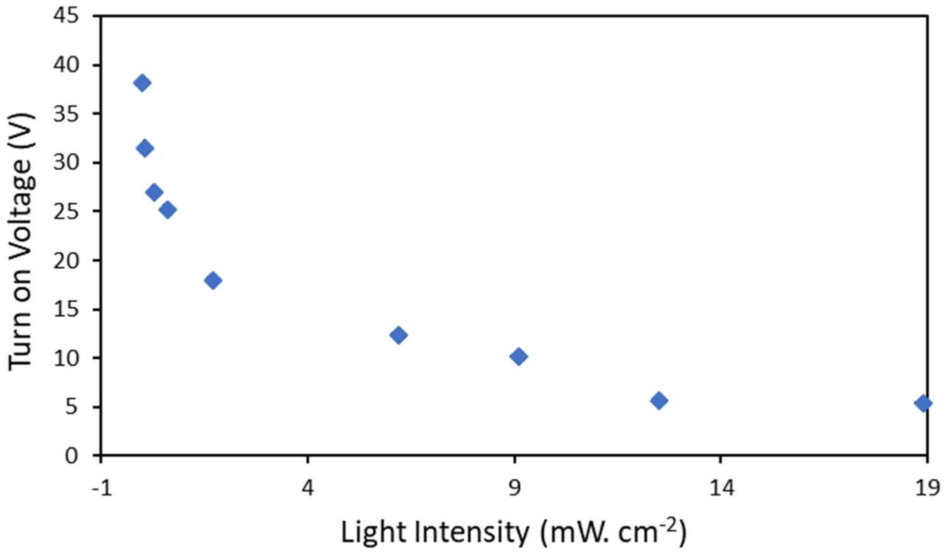

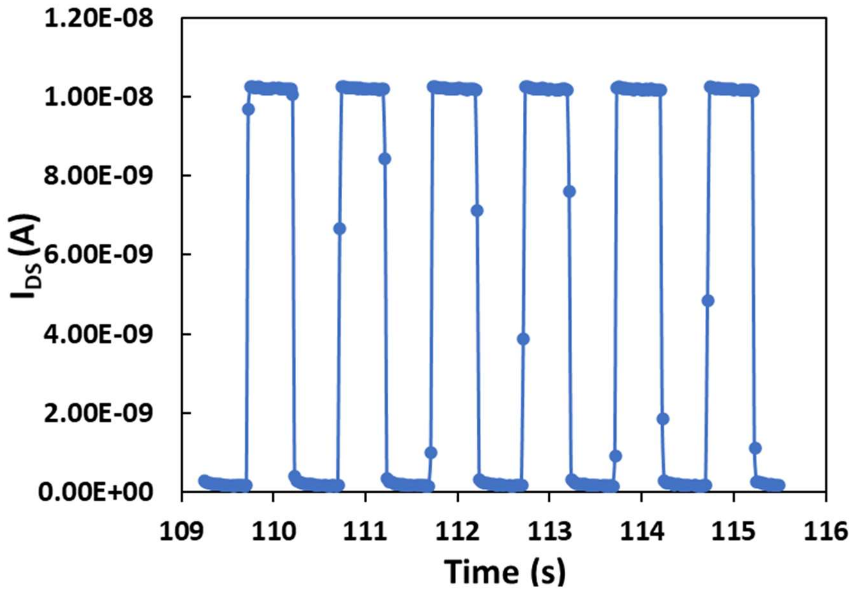

Figure S14 The turn on voltage of CP Photo-FET shift under increasing illumination intensity and temporal response showing fast photo switching. Turn on voltages were obtained by the x-intercepts of the slope of $I_{DS}^{1/2}$ vs V_{GS} plot.

Discussion on the mechanisms of CP responsiveness in chiral photoFETs

Under illumination, the photoresponse of an n-type photoFET is governed by the photoconductive and photovoltaic effects.[19,20] In the photoFET off-state ($V_{GS} < V_{th}$, where V_{th} is the threshold voltage), the photoconductive effect dominates and the majority of charges present in the photoactive layer are photogenerated by the incident light.[19,20] As a result, for fixed V_{GS} and V_{DS}, this leads to a linear relationship between the device photocurrent ($I_{DS,ph}$) and the total incident optical power (P_{opt}), that is:

$$I_{DS,ph}=AP_{opt} \qquad \textbf{Equation S1}$$

where A is a proportionality constant.[19,20] This linear behaviour is demonstrated in our own photoFETs in **Figure S15a** for $V_{GS} < V_{th}$, where fit parameters of $A = (32.80 \pm 0.82) \times 10^{-12}$ A/W and $A = (32.35 \pm 0.40) \times 10^{-12}$ A/W are obtained for $V_{GS} = 0$ V and $V_{GS} = 5.62$ V, respectively.

When $V_{GS} > V_{th}$ (the "on-state"), the photoFET photoresponse is dominated by the photovoltaic effect.[19,20] In this regime, V_{th} is decreased under illumination relative to the dark case, as observed in **Figures 3a,b**.[19,20] This is caused by the accumulation of photogenerated holes at the source electrode, which reduces the barrier for electron injection. In this case, the relationship between $I_{DS,ph}$ and P_{opt} takes the form:

$$I_{DS,ph}=g_m \Delta V_{th}=\frac{BkT}{q}\ln\left(1+\frac{\eta q P_{opt}}{I_{pd}E}\frac{a_d}{a_b}\right) \qquad \textbf{Equation S2}$$

where g_m is the transconductance, ΔV_{th} is the shift in threshold voltage, B is a proportionality constant, η is the photogeneration quantum efficiency, a_d is the device area, a_b is the beam area, I_{pd} is the dark current for minority charges (holes), E is the photon energy and all other symbols have their usual meanings.[19,20]

To demonstrate the consistency of our device behaviour with the photovoltaic mode of operation, **Equation S2** was fitted to plots of $I_{DS,ph}$ as a function of unpolarised light intensity (**Figure S15b**), using B and η/I_{pd} as fitting parameters. As shown, the data was found to be in good agreement theory for $V_{GS} > V_{th}$, with $R^2 > 0.98$ for all fits. B is approximately constant for these fits with increasing V_{GS} (ca. 1×10^{-7} S), however, η/I_{pd} was found to increase. The latter observation suggests an enhancement of η by the transverse electric field of the gate and/or a decrease in I_{pd} due to the depletion of photogenerated holes.

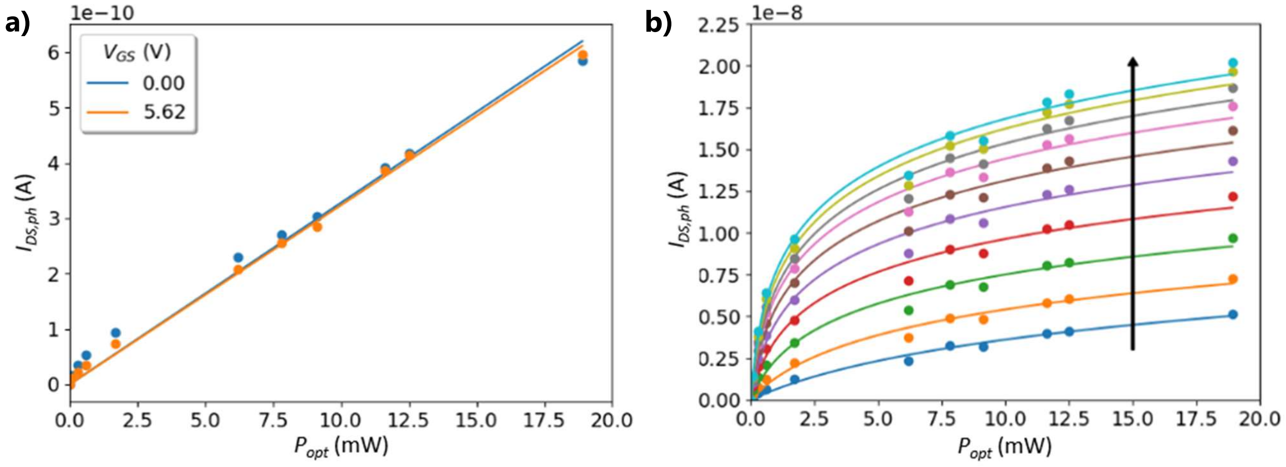

Figure S15 Intensity dependence (unpolarised light) of photoFET $I_{DS,ph}$ in the two device modes: photoconductive (**a**) and photovoltaic (**b**). Filled circles indicate experimental data and solid lines represent fits to **Equation S1** (**a**) and **Equation S2** (**b**). Black arrow indicates direction of increasing V_{GS} from 44.94 V to 95.51 V.

To summarise the above, the photoresponse of an n-type phototransistor is the combination of two effects, both of which increase $I_{DS,ph}$. The first is the photogeneration of electrons, which increases the majority carrier density in the photoactive layer and is the dominant effect for $V_{GS} < V_{th}$. The second is the reduction of the injection barrier for electrons at the source electrode due to the accumulation of photogenerated holes, which dominates when $V_{GS} > V_{th}$.

To explain the amplification of g_{ph} relative to g_{abs} for our devices, we propose that two cooperative mechanisms are at work, based on the two regimes of device behaviour. For the same incident intensity of L-CPL and R-CPL, we expect a greater number of photogenerated electrons when illuminated by the strongly absorbed handedness (SAH) relative to the weakly absorbed handedness (WAH)[1]. This is observed as a greater photocurrent for the SAH in the photoconductive regime of **Figures 3a,b**. We also expect a greater number of photogenerated holes for the SAH, which can accumulate at the source electrode. This leads to a lower V_{th} for the SAH relative to the WAH, meaning that for a narrow range of voltages, the device is in the on-state ($V_{GS} > V_{th}$) for the SAH and is in the off-state ($V_{GS} < V_{th}$) for the WAH. This leads to a peak in g_{ph} at around $V_{GS} = V_{th}$ for the devices under illumination which is observed in **Figures 3a** (This is less clear in **Figure 3b**, perhaps due to the increased photodegradation in these devices – see next section).

Whilst this model is consistent with many characteristics of device behaviour, it is unclear at present whether this model is sufficient to fully explain the enhancement of g_{ph} relative to g_{abs} and further study is required in this area.

Racemic Devices and the Influence of Device Photodegradation

To identify any artefacts in the procedure used to measure the CP response of enantiopure devices and to compare the performance of enantiopure devices relative to their racemic starting materials, the device characteristics of a racemic (*anti,R/*S)16,17-bis[60]PCBM photoFET were investigated. The transfer characteristics of this device in the absence of light and under L-CPL, R-CPL and unpolarised illumination is shown in **Figure S16.**

Under dark conditions, this device exhibits analogous transfer characteristics to that of the enantiopure (*anti,R*)16,17-bis[60]PCBM device, albeit with a greater turn-on voltage of 50 V. Also, as might be expected, the transfer curves of these devices under unpolarised light and CPL of equal intensity appear to be similar, suggesting limited CP selectivity. However, calculating g_{ph} for transfer curves measured under L-CPL (**Figure S17**, Left #1) followed by R-CPL (Right #1) indicates a significant g_{ph} value of up to 0.15 (g #1) for this racemic device. To further investigate the origin of this anomalous g_{ph}, the order in which photoFETs were exposed to CPL was reversed – first testing under R-CPL (**Figure S17**, Right #2) followed by L-CPL illumination (Left #2). This led to a calculated g_{ph} value of a similar magnitude but

[1] For $g_{abs} > 0$, SAH and WAH are L-CPL and R-CPL respectively. For $g_{abs} < 0$, SAH and WAH are R-CPL and L-CPL.

opposite sign (-0.10) to the g_{ph} calculated previously. This finding, in addition to observations of device performance degradation under intense light exposure nearing the UV, suggested that these devices are subject to photodegradation which leads to a non-negligible uncertainty in the calculated value of g_{ph}.

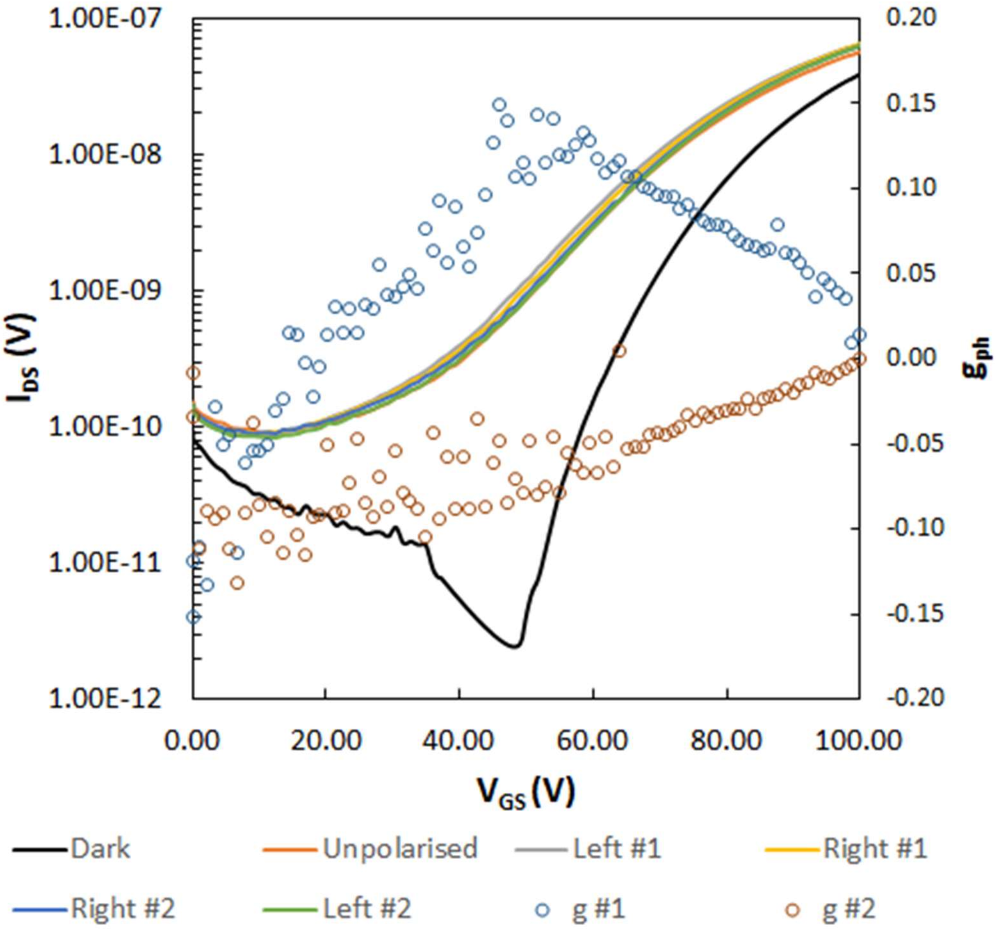

Figure S16 Transfer curves of a racemic (*anti,R/S*)16,17-bis[60]PCBM photoFET in the absence of light and under L-CPL, R-CPL and unpolarised illumination at an intensity of 3.4 mW cm^{-2}. This device has been measured under each handedness of CPL twice in the order: Left #1, Right #1, Right #2 and Left #2. g #1 and g #2 correspond to the photocurrent dissymmetry of the racemic devices using the photocurrents of Left #1 and Right #1, and Left #2 and Right #2, respectively.

In enantiopure devices, the observed photocurrent dissymmetry is thus a combination of both the "true" g_{ph} and an artefact g_{ph} which is solely a result of device photodegradation over time, the sign of which is determined by the order of exposure of a given device to L-CPL and R-CPL.

Where present, the artefactual g_{ph}, is minimised by reducing the intensity of the illuminating CPL (thus reducing photodegradation) whilst also ensuring that the photoresponse of the device remains measurable. Evidence of this procedure in action is shown in **Figure S17**. For our (*anti,R*)16,17-bis[60]PCBM device, this photodegradation leads to a difference in dissymmetry of approximately 0.1 under reversal of CPL exposure. To account for degradative g_{ph}, to

first approximation, we report the average of g #1 and g #2 as our device photocurrent dissymmetries and half of the difference of g #1 and g #2 as the uncertainty for this quantity.

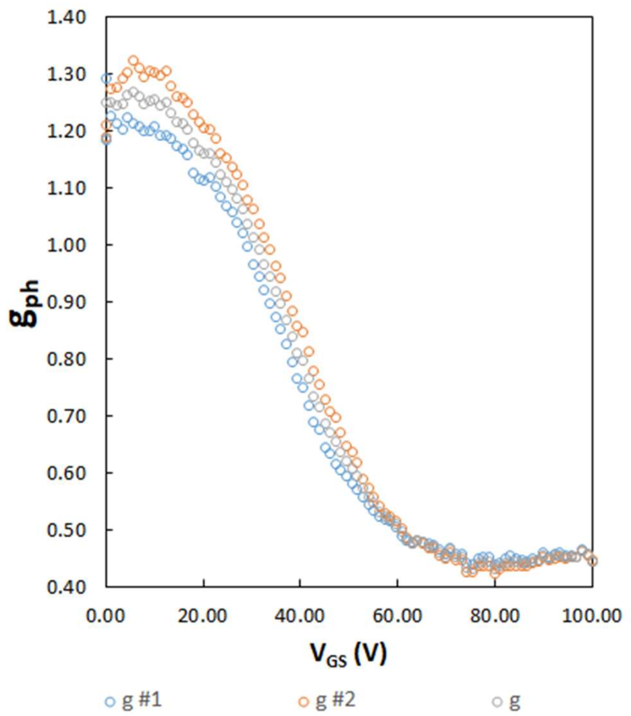

Figure S18 Photocurrent dissymmetry of as a function of gate voltage for our (anti,R)16,17-bis[60]PCBM device for both orders of CPL exposure: L-CPL followed by R-CPL (g #1) and R-CPL followed by L-CPL (g #2). Here g is the average of g #1 and g #2 and is the value reported in the main text.

Inversion of g_{ph} Relative to g_{abs}

In our enantiomerically pure devices, we observe that the photoFET g_{ph} values take the opposite sign to the g_{abs} values of their corresponding films when illuminated at 405 nm. Similar behaviour has already been documented in the literature in prior CP photodetector studies, where devices containing thick active layers were found to exhibit a preferential photoresponse to the opposite handedness of CPL to which they preferentially absorb [21]. This is a result of the "filtering out" of the preferentially absorbed handedness of CPL by the CP selective layer before reaching the photoactive region (the donor-acceptor interface in the case of a photodiode or the channel in the case of a transistor). The consequence of this is that the weakly absorbed handedness has a higher intensity at the photoactive layer and dominates the CP selective response of the device. This leads to the observed inversion of g_{abs} relative to g_{ph}.

References

[1] M. Lenes, G.-J. A. H. Wetzelaer, F. B. Kooistra, S. C. Veenstra, J. C. Hummelen, P. W. M. Blom, *Advanced Materials* **2008**, *20*, 2116.

[2] T. Liu, I. Abrahams, T. J. S. Dennis, *Journal of Physical Chemistry A* **2018**, *122*, 4138.

[3] W. Shi, X. Hou, T. Liu, X. Zhao, A. B. Sieval, J. C. Hummelen, T. J. S. Dennis, *Chemical Communications* **2017**, *53*, 975.

[4] M. J. Frisch, G. W. Trucks, H. B. Schlegel, M. A. R. G. E. Scuseria, J. R. Cheeseman, *Gaussian 16, Revision C.01*, Gaussian, Inc., , Wallingford CT, **2016**.

[5] S. Mai, F. Plasser, J. Dorn, M. Fumanal, C. Daniel, L. González, *Coordination Chemistry Reviews* **2018**, *361*, 74.

[6] S. A. Mewes, J. M. Mewes, A. Dreuw, F. Plasser, *Physical Chemistry Chemical Physics* **2016**, *18*, 2548.

[7] F. Plasser, *Journal of Chemical Physics* **2020**, *152*, 084108.

[8] H. A. Favre, W. H. Powell, *Nomenclature of Organic Chemistry*, Royal Society Of Chemistry, **2013**.

[9] A. Rassat, P. W. Fowler, B. de la Vaissière, *Chemistry – A European Journal* **2001**, *7*, 3985.

[10] C. Thilgen, A. Herrmann, F. Diederich, *Helvetica Chimica Acta* **1997**, *80*, 183.

[11] T. Bruhn, A. Schaumlöffel, Y. Hemberger, G. Bringmann, *Chirality* **2013**, *25*, 243.

[12] F. Plasser, M. Wormit, A. Dreuw, *Journal of Chemical Physics* **2014**, *141*, 024106.

[13] S. Eder, D. Yoo, W. Nogala, M. Pletzer, A. Santana Bonilla, A. J. P. White, K. E. Jelfs, M. Heeney, J. W. Choi, F. Glöcklhofer, *Angewandte Chemie International Edition* **2020**, *59*, 12958.

[14] Z. Chen, J. I. Wu, C. Corminboeuf, J. Bohmann, X. Lu, A. Hirsch, P. V. R. Schleyer, *Physical Chemistry Chemical Physics* **2012**, *14*, 14886.

[15] M. P. Johansson, J. Jusélius, D. Sundholm, *Angewandte Chemie International Edition* **2005**, *44*, 1843.

[16] T. Woller, P. Geerlings, F. de Proft, B. Champagne, M. Alonso, *Journal of Physical Chemistry C* **2019**, *123*, 7318.

[17] M. Garcia-Borràs, S. Osuna, J. M. Luis, M. Swart, M. Solà, *Chemical Society Reviews* **2014**, *43*, 5089.

[18] A. Hirsch, Z. Chen, H. Jiao, *Angewandte Chemie - International Edition* **2000**, *39*, 3915.

[19] T. P. I. Saragi, J. Londenberg, J. Salbeck, *Journal of Applied Physics* **2007**, *102*, 046104.

[20] K. J. Baeg, M. Binda, D. Natali, M. Caironi, Y. Y. Noh, *Advanced Materials* **2013**, *25*, 4267.

[21] J. Gilot, R. Abbel, G. Lakhwani, E. W. Meijer, A. P. H. J. Schenning, S. C. J. Meskers, *Advanced Materials* **2010**, *22*, E131.
